# Trait preference trade-offs among maize farmers in western Kenya

**DOI:** 10.1016/j.heliyon.2021.e06389

**Published:** 2021-03-12

**Authors:** Paswel P. Marenya, Rosina Wanyama, Solomon Alemu, Vincent Woyengo

**Affiliations:** a International Maize and Wheat Improvement Center (CIMMYT), Nairobi, Kenya; b CIMMYT, Nairobi, Kenya; c Alliance Bioversity-CIAT, Addis Ababa, Ethiopia; d Kenya Agricultural and Livestock Research Organisation, Kakamega, Kenya

**Keywords:** Choice experiment, Maize, Mixed logit, Trait preference

## Abstract

Farmers inevitably make trade-offs when choosing which crop varieties to grow based on each variety's unique strengths (and weaknesses). This study uses choice experiment data from 1288 maize farmers from western Kenya and applies a mixed logit model to estimate willingness to sacrifice yield as an experimental devise to measure such trade-offs when farmers are called upon to chose varieties with varying levels of desirable agronomic or consumption traits. We find that men and women respondents had similar preferences for maize traits, but differed in the rate of tradeoffs between traits. Women respondents appeared to make larger yield sacrifices for tolerance to drought, *Striga* weed and good storability than men. Men showed higher willingness to sacrifice yield for closed tip. Implications for gender-sensitive maize breeding and seed market development are drawn.

## 1 Introduction

### 1.1 Background and motivation

Maize remains the single most important staple for calorie nutrition and, in many cases, a source of cash income for many families in Kenya. Maize provides more than 30% of total dietary calorie intake and nearly 70% of daily pa capita cereal consumption. Moreover 85% of the population consumes at least some maize, confirming its ubiquitous consumption in the country (see Kariuki et al., 2020). By some estimates (FAO, 2008; Schroeder et al., 2013) the crop accounts for 20% of total agricultural production and 25% of employment. As a result, the maize crop provides a key barometer for food security in the country. The per capita annual consumption is variously estimated to be between 88 -103 kg (Short et al. 2012; Abate et al., 2015). In terms of aggregate consumption, there are recurring deficits in supply that have to be met by imports (Kariuki et al., 2020). As reported by Kariuki et al. (2020), maize deficits in Kenya are caused by low productivity growth (about 2%) compared to population growth of 3.5%. Moreover four million vulnerable Kenyans typically need food aid every year (with an estimated maize consumption of 114 kg/yr) as per a recent government publication (Republic of Kenya, 2019). For strategic food security reasons, the policy ambition is to reduce (or eliminate these imports) through increased productivity, with a stated potential of 157% yield increase (Republic of Kenya, 2019).

Unfortunately, the productivity of maize in the country has been on a declining trend over time. Currently, maize yield potential stands at 6 t/ha, yet only an average of 1.8 t/ha is realized (Njagi et al., 2017; Ouma et al., 2006). The decline in production is attributed to several factors, including low and inconsistent use of the most improved varieties, poor agronomic practices, and inappropriate fertilizer application. In efforts to ensure national maize supplies meet rising demand, a steady pipeline of new varieties is needed (Marechera et al., 2016). The aim is to maintain productivity through consistent genetic gains and to respond to emerging climatic and environmental stressors (Cairns et al., 2013). The Kenya national plant registry suggests that there are about 366 varieties registered in the country as of 2019 (KEPHIS, 2019). A study by Abate et al. (2015) estimated that the number of commercialized varieties in 13 African countries (including Kenya) was 500, suggesting an average of 38 commercialized varieties in each of the 13 countries. The apparent disjuncture between the large number of registered varieties and those that are commercially available suggests that while the breeding pipelines are robust, the commercialization and delivery parts of the seed systems are still left somewhat wanting. One reason can be that the development of new varieties is not sufficiently client-focused; therefore, many varieties fail to gain market share (Witcombe and Yadavendra, 2014).

Three critical considerations should guide variety development: a) What varieties do farmers currently prefer, and what attributes/traits do these varieties possess? b) What new traits are being demanded by farmers (and other value chain actors) but are currently not available in the existing varieties? c) What trade-offs are farmers willing to make when confronted with a bundle of traits, given that no single variety can possess all desirable traits? Addressing these questions can guide improvement of the selection criteria by maize breeders to help them design and optimize market-driven and farmer-centered breeding programs. Products from such programs will likely have higher chances of being commercialized and gaining market share. This study was designed to contribute to the understanding of the key trade-offs farmers are willing to make in choosing varieties. This information is essential in informing breeding strategies regarding the minimum traits required in future varieties currently under development.

The motivation for this research was to provide a farmers’ lens to variety attribute prioritization. There are large investments by governments and development partners in maize breeding programs in Africa. At the same time, there are many on-shelf varieties with limited market share (as shown by the large numbers of registered varieties with only a fraction of those being actively commercialised). One of the problems may be lack of conformity of the new varieties with farmers’ priorities. Yet this type of preference prioritization is often lacking. Moreover, these preferences need to be studied empirically if the necessary tradeoffs (prioritization) are to be identified. To the best of our knowledge ours is the first paper to conduct this empirical prioritization in the mid-altitude agro-ecozones of smallholder maize farming systems of western Kenya. Our paper attempts to fill this gap by conducting a multi-criteria choice experiment, typically used in market analysis to understand farmers priorities in chosing maize varieties. The aim is to contribute information that can help future breeding programs to produce new varieties that are consistent with the farmers’ stated preferences and with better commercial potential, thereby avoiding the scenario where finished varieties stay on-shelf for many years.

### 1.2 Literature overview

Several studies have previously explained farmers’ preferences for attributes in different crop varieties. In Zimbabwe, Kassie et al. (2017) examined farmers’ preferences and willingness to pay for drought tolerance in maize using choice experimental data. Their results showed that drought tolerance, grain yield, covered cob-tip, cob size, and semi-flint texture were the most preferred traits. For instance, farmers were willing to pay a premium for drought tolerance that was about three to seven times higher than all other attributes. Also, using choice experiment data, Asrat et al. (2010) found that farmers in Ethiopia were willing to forgo some income or yield to obtain more stable and environmentally adaptable crop varieties. Ward et al. (2013) reported that although farmers in India were willing to pay more for high yielding rice than local varieties in all conditions, they were willing to pay significant amounts for seeds that outperformed the local varieties under drought stress conditions even without yield advantages under normal circumstances. These findings emphasize that although the yield is an overarching attribute, in some instances, non-yield traits are equally or even more important to farmers when presented with a bundle of both yield and non-yield traits.

To this end, most studies looking at trait preference and the associated willingness to pay using choice experimental data have used seed prices to assess the demand for selected traits (Kassie et al., 2017; Ward et al., 2013; Asrat et al., 2010). In the Kenyan market, maize seed prices are almost dictated by a single player—the Kenya Seed Company—which has about 80% market share (Smale and Olwande, 2011). In western Kenyan seed markets, different seed companies charge different prices but have a uniform price for all of the company’s seed products. Various products from a seed company have specific trait combinations and unique strengths. However, seed pricing does not seem to be based on particular traits, but perhaps on inter-company market competition (Smale and Olwande, 2011).

This paper, therefore, makes the following contributions to the literature on farmer-centered maize seed systems. First, we use a novel approach to explicitly determine the value farmers place on various attributes, using yield as a “price” variable. This is important due to the minimal seed price differentiation mentioned above. Given that seed prices are not based on trait variations, they do not accurately reflect the attribute choices that farmers make. Secondly, we use a gender-disaggregated data set that elicits choice responses from individual men and women within the household. It is well known that intra-household differences in resources, ability to make choices (agency), and similar factors constitute the bulk of gender issues in agricultural development. Thirdly, we combine stated choice and bid auction methods to improve the data on maize variety choices by supplementing the choice experiment data with an auction method that is more incentive compatible. This study improves on existing studies that tend to rely only on preference voting (ranking), typical in participatory variety evaluation but devoid of means to enable farmers to make trade-offs in the choice process. To the best of our knowledge, ours is the only paper that uses the two methods in maize variety choice.

The rest of this paper is organized as follows. The next section outlines the data sources and the methods used in this study. The methods section includes descriptions of the choice experiments and the auction procedures. The methods section ends with a discussion of the econometric strategies. The methods section is followed by a presentation of the key results, discussed from multiple angles. A final section concludes by summarizing this study with key take-away messages.

## 2 Materials and methods

### 2.1 Sampling and data

The data used in this paper came from personalized interviews involving the administration of a choice experiment (CE) and auction bids, as explained in the next sub-section. Moreover, a structured questionnaire was administered to each participant to collect data on demographic and farm characteristics. The selection of the households was done using a three-stage sampling technique, combining purposive and random sampling. The first stage involved the selection of counties and sub-counties where a CGIAR/CIMMYT-led consortium implemented the Stress Tolerant Maize for Africa project from 2016–2020 (and later the Accelerating Genetic Gains in Maize and Wheat project from May 2020 onwards). Both projects were meant to inrease the supply of stress-tolerant maize varieties for resource-limited smallholder maize-growing regions of west, eastern and southern Africa. For the purporses of this study, the locations chosen were those dominated by small-scale farmers in mid-altitude ecological zones in western Kenya. The counties chosen were Busia (Matayos and Butula sub-counties), Kakamega (Mumias East and Butula sub-counties) and Siaya (Gem and Ugenya sub-counties). The second stage procedure involved the selection of villages within these project zones. The number of villages were selected using a sampling design that made explicit use of the population—in particular, “the probability proportional to size (PPS)” sample design^1^. Therefore, the larger sub-counties was assigned two to three villages, while the rest were assigned one village each. In each sub-county, the next administrative unit was the ward. One ward was randomly selected based on the predominance of maize in those wards. The selection of villages to include in the study was also done randomly from the entire list of villages in a county. Randomization was also used in the final stage to select 40 households from each randomly selected village. Based on this procedure, a total of 719 households (40 households per village) were selected from the three counties, six sub-counties, and 18 villages. In each household, efforts were made to administer the CE to two adult members of the household, typically the husband (or typically a man, with 53% of households being male-headed). Therefore, a total of 1288 respondents participated in the survey: 53% being household heads, 33% being spouses, and 14% being other household members. Sampled counties, sub-counties, wards, as well as the number of villages and households are presented in Table 1 below. The structured questionnaire elicited information from respondents on aspects such as household demographics, education, household occupations, marital status, relationship to the household head, ownership of assets, and intra-household maize variety decision making (see Table 2).

**Table 1:** Sampled Counties, Sub-counties, villages, and households in western Kenya, 2017.

County	Sub-county	Wards	Number of villages	Number of households
Kakamega	Mumias East	Malaha-Isongo-Makunga	3	119
	Butere	Marama Central	1	40
		Marama South	2	80
Siaya	Gem	Gem South	2	80
		North Gem	1	40
	Ugenya	North Ugenya	1	40
		Ukwala	1	40
		East Ugenya	1	40
Busia	Matayos	Busibwabo	2	80
		Mathayos South	1	40
	Butula	Marachi East	1	40
		Elugulu	1	40
		Marachi Central	1	40
Total	**6**	**13**	**18**	**719**

**Table 2:** Sample characteristics for three counties (Busia, Kakamega, Siaya) in western Kenya.

Variable	Description	Experiment A (*n* = 319)	Experiment B *(n* = 330)	Experiment C (*n* = 320)	BDM *(n* = 319)
Respondent type	Household Head (%)^A^	52.35	51.52	51.25	55.80
	Spouse (%)	34.17	33.94	31.25	34.80
	Another adult member (%)	13.48	14.55	17.50	9.40
Sex	=1 if respondent is male (%)	44.83	44.55	43.44	45.45
Age	=1 if respondent is below 35 years	39.50	33.15	37.19	36.99
Education	=1 if respondent has post primary education (%)	23.20	26.36	29.26	27.27
Total land owned	Acres	1.52	1.70	40.00	1.98
Land under maize	Acres	1.05	1.00	1.83	1.07
Maize net buyer	=1 if respondent is a net maize buyer (%)	39.81	43.33	39.66	-
Occupation	=1 if main occupation of respondent is agriculture (%)	67.71	68.48	68.75	70.53
Distance to the nearest trading center	Km	2.89	2.14	4.00	2.98
County	Kakamega (%)	33.23	46.36	25.63	27.90
	Siaya (%)	31.03	28.48	41.88	31.03
	Busia (%)	35.74	25.15	32.50	41.07
*Number of households*		*183*	*179*	*179*	*178*

^A^The proportion of families headed by men was 67% in this sample.

### 2.2 Basic data descriptions

The descriptive statistics presented in Table 2 show that the respondents were roughly equally distributed between the three CEs. In the four experiments, 43–45% of the respondents were household heads. On average, 51–56% of the respondents were aged below 35 years, while less than 30% had post-primary education. Most households were located less than 4 km from the nearest trading centers, implying that these households are generally closer to markets. As expected, a majority of the respondents (more than 67%) rely on agriculture as their primary occupation, which is typical for most rural families in Kenya. Note that the share of household heads is lower than the 80% or higher that is typically observed in western Kenya or the eastern and southern Africa region (Laiglesia and Morrisson, 2008). When we just count the number of families headed by mean in our data it was 67%. This is because we interviewed multiple respondents within the same household. This approach (of interviewing men and women within the same household) is consistent with the most recent understanding of gender issues. Capturing data from both men and women within the household generates true intra-household sex-disaggregated data suitable for gender analysis (Doss et al., 2018; Peterman, 2011). This important because as Doss et al. (2018) shows, the vast majority of women live in dual adult households and therefore the bulk of gender issues starts with women in these (typically male-headed) households.

### 2.3 Choice experiment (CE)

A discrete CE was conducted to generate data on maize variety preferences. Specifically, the CE required participants to choose one of two varieties in ways that would compel them to make trade-offs—an improvement over other ranking methods such as contingent valuation (Fonta et al., 2018; Hynes et al., 2011; Bennett and Adamowicz, 2001). Choice experiments have been applied in diverse fields such as choice of modes of transportation (Hensher et al., 2005), health (Hole and Kolstad, 2012), marketing (Feit et al., 2010; Louviere et al., 2010; Louviere and Woodworth, 1983), and environmental economics (Veettil et al., 2011). More recently, CEs have been applied in agricultural value chains to evaluate the demand for nutritious foods and food safety (Wanyama et al., 2019; Ortega et al., 2011), preferences for crop traits (Kassie et al., 2017; Ward et al., 2013; Asrat et al., 2010), preferences for weather index insurance products (Sibiko et al., 2018), and the design of sustainability standards (Meemken et al., 2017). Others include system characteristics such as product marketing options and supply chain differentiation (Ochieng et al., 2017; Schipmann and Qaim, 2011), and input support policy preferences (Marenya et al., 2014).

### 2.4 Auction experiment using the Becker-DeGroot-Marschack (BDM) framework

While the CEs provide thought experiments that allow participants to make trade-offs in their choices, willingness to pay using hypothetical valuation mechanisms such as CEs can still result in hypothetical bias because the actual choice does not involve any real cost (Murphy et al., 2005; Little and Berrens, 2004). However, using incentive-compatible mechanisms like the Vickrey, random *n*^th^ price auction, or Becker-DeGroot-Marschack (BDM) frameworks can reduce such biases (Lusk and Shogren, 2007; Vickrey, 1961; Becker et al., 1964). For robustness check, we also conducted an auction experiment following the BDM framework. The advantage of the BDM framework is its simplicity and convenience for use in a wide range of field situations, thus increasing the external validity of estimates, albeit with some loss of accuracy compared to more complicated incentive-compatible methods (Lusk and Rousu, 2006; Noussair et al., 2004).

### 2.5 Implementation of CEs and BDM

To reduce the complexity of the CEs, we designed four separate experiments in which three were CEs, and one was an auction experiment in the manner of the BDM framework. In each household, the CEs and BDMs were conducted *separately* with *both* the household head and the spouse, unless there was only one adult responsible for maize production, in which case only one member (typically the household head) was interviewed. Each household was assigned randomly to one of the four (three CEs and one BDM) experiments using an excel random number generator. To minimize biases, the BDM procedure was further divided into two sub-categories (low-to-high and high-to-low). To generate the CE sessions, we used a statistical D-efficient design using the Ngene software (ChoiceMetrics, 2012). Table 3 shows the sample distribution among the four experiments. The traits and trait levels used for the CEs are described in Table 4 below.

**Table 3:** Distribution of respondents across experimental groups.

Experiment	Respondent categories		Sex of all respondents		Total
	Spouse	Other adults	Male	Female	
Choice experiment A	109	43	143	176	319
Choice experiment B	112	48	147	183	330
Choice experiment C	100	56	139	181	320
BDM Auctions	111	30	145	174	319
**Total sample**	**432**	**177**	**574**	**714**	**1288**

**Table 4:** Summary of maize traits used in the experiments.

Experiments	Trait††	Description	Levels	Reference
A, B, C	Yield (90 kg bags/acre)	Amount of dry grain that farmers can obtain per unit of land, usually in bags of 90 kg per acre. Results from focus group discussions showed that in Siaya it was a maximum of 28 bags/ha, and an average of 15 bags/acre, In Busia, it was average of 10 bags/acre, a low yield of 5 bags/acre and high of 18 bags/acre. In Kakamega, the yield was about 30 bags/acre at the high end and, on average, was 20 bags/acre.	6, 15, 20	As shown in the description column
A	Maturity period	The time it takes from planting to when the maize can be harvested for cooking or roasting.	Matures in 3 months or less, matures after 3 months	Matures after 3 months
	Shelf life (Storability)	This described how long dried grains can be stored before they spoil from damage by storage pests (weevil or grain borer).	Poor, good	Poor
	Grain size	The size of the individual maize grain as subjectively assessed by the eye.	Small, medium, large	Small
B	Husk cover	The cob husks cover the whole cob including the tip without any opening at the tip.	Open tip, Closed tip	Open tip
	Grain weight	Feels heavy when “weighed” in the palm. The feeling of “heaviness” when scooped in one hand and “sifted” up and down on the palm.	Light, Heavy	Light
	Top-dressing requirements	How much top-dress fertilization is required after fertilizer application?	Low, High	Low
C	Drought tolerant	When there is a mid-season dry spell, drought-tolerant maize stays green and does not lose pollen, so grain filling still occurs. The variety that was moderately drought tolerant was reported to yield only 30% of typical yields, but the common drought-tolerant varieties could yield about 46–60% of the typical yield if there was moisture stress during the mid-season. This was referred to as moderate (non-catastrophic) drought.	Not tolerant, tolerant	Not tolerant
	Lodging resistant (Sturdiness)	Lodging resistance refers to the ability of the maize plant to remain erect in windy weather or storms. This is related to how tall the variety grows. Taller varieties tend to break and fall over during wind and storms. This leads to large yield losses as the plants break and die and, depending on the stage of maturity, little or no grain can be harvested.	Not resistant, moderately resistant, completely resistant	Not resistant
	*Striga* tolerant	The variety can tolerate *Striga* infestation without too much stunting. *Striga hermonthica* (purple witchweed) is a plant parasite of maize (and other cereals). It depletes the host nutrients by inserting its roots into those of the host's root tissues. Depending on the severity of infestation and tolerance of the host variety, the result is extremely low (or no) yields.	Not tolerant, tolerant	Not tolerant

†† All the 10 traits were also used in the BDM auctions.

### 2.6 Ethics review of the research protocols

Prior to the implementation of the field surveys, this study was reviewed and cleared by the Institutional Research Ethics Committee (IREC) at the lead author’s institution. The IREC is responsible for research ethics review and compliance monitoring. The questionnaires and the choice experiment and BDM questions were sent to IREC and were deemed as being *“...regular household attitude and opinion elicitation. Nevertheless, the research is extremely benign and there is no danger whatsoever of putting the respondents in harm’s way. The proposal is clear on how they will handle the data...”* Each questionnaire was designed in a computer assisted personal interview (CAPI) program to ensure that the interview did not start until a verbal consent was given. The CAPI program was designed to bring up the consent statement by default and only after entering the respondents consent, would the skip pattern allow the interviewer to open the question modules. To safeguard the right of the respondent the consent statement made it clear that the respondent had a right to stop the interview at any stage, request the data to be expunged and they were not required to explain the reason for terminating the interview. This study was conducted in liaison with the Kenya Agricultural and Livestock Research Organization and complied with research regulations and protocols in Kenya.

### 2.7 The choice theory

Choice experiments are grounded in Lancaster’s consumer choice theory, which suggests that utility is derived from the underlying characteristics or attributes of a good (Louviere et al., 2000) and that consumer behaviour can be cast in the Random Utility (RUT) framework as in Thurstone (1927), McFadden (1973), and Adamowicz et al. (1998). In the present study, farmers were presented with two stylized maize varieties with different attribute bundles at each choice scenario. In general terms, for the farmer faced with J varieties, the utility of variety j is $U_{ij}$. By choosing j, we assume that $U_{ij}$ is the maximum among the J utilities. Hence, the statistical model is driven by the probability that variety j is chosen:

$Prob\;\left(u_{ij}>u_{ik}\right)\;$ for all j≠k;k=1,2…J

Following the random utility reasoning, farmers’ utility for a particular maize variety is modeled as follows:

(1) $$U_{ij}=V_{ij}+\varepsilon_{ij}$$

In Eq. (1), where $V_{ij}$ is the explainable component of the utility of maize variety j for farmer i, and $\varepsilon_{ij}$ is the random component of the utility function. Let $Y_{i}$ be a random variable that indicates the choice made. Given a choice between alternatives j and k, the probability that farmer i chooses alternative j is such that

$Prob\;(Y_{i}=j\;|\text{j},\text{k}=\text{Prob }\left[\left(V_{ij}+\varepsilon_{ij}\right)>\left(V_{ik}+\varepsilon_{ik}\right)\right]$ for all j≠k;k=1,2…J

The utility $U_{ij}$ therefore depends on the attributes of the variety j, and farmer $i^{\prime }s$ individual characteristics. Assuming x is a vector of farmer characteristics, and z is a vector of characteristics of the alternatives, it follows (in Eq. (2))

(2) $$V_{ij}=\beta x_{ij}+\alpha^{\prime }z_{i}$$

where β and α are the corresponding vectors of parameters to be estimated.

### 2.8 Econometric strategy: the mixed logit model

Discrete choice models have often used multinomial logit (MNL) and conditional logit (CL) models (Hensher and Greene, 2003; McFadden and Train, 2000; Hoffman and Duncan, 1988; McFadden, 1973). The basic notion is a probability distribution function of the maximum of a series of random variables (Gumbel, 1958). The MNL focuses on the individual as the unit of analysis and uses the individual’s characteristics as explanatory variables, while CL focuses on the choice alternatives, which are used as explanatory variables (Hoffman and Duncan, 1988). These models can be specified as follows:

(3) $$P_{ij}=\exp\left(X_{i}\beta_{j}\right)/\overset{J}{\underset{k=1}{\sum }}\text{exp}\left(X_{i}\beta_{k}\right)\;\text{for}\;\text{multinomial}\;\text{logit}$$

(4) $$P_{ij}=\exp\left(Z_{ij}\alpha \right)/\overset{J}{\underset{k=1}{\sum }}\text{exp}\left(Z_{ik}\alpha \right)\;\text{for}\;\text{conditional}\;\text{logit}$$

where in Eq. (3) X represents the characteristics of individual i, and in Eq. (4), $Z_{ij}$ are the characteristics of alternative j for individual i, while β and α are the corresponding vectors of parameters that represent the influence of individual and attribute characteristics. The specification of the MNL and CL models require that the unobserved effects are independently and identically distributed (IID) across the alternatives in the choice set, according to the extreme type 1 distribution. This assumption results in a more rigid property of “independence from irrelevant alternatives” (IIA) (Hoffman and Duncan, 1988; Ben-Akiva and Bierlaire, 1999). The IIA property assumes that everybody in the population has a homogeneous preference structure, and therefore restricts the $\beta^{\prime }s$ to be the same for all members of the population (Holmes and Adamowicz, 2003). That is, given $\varepsilon_{ij}$ for all i, j, the probability that a given individual i chooses alternative j within the choice set $S_{i}$ is given in Eq. (5) below as

(5) $$p\left(j|S_{i}\right)=\frac{\text{exp}\left(\mu v_{i}\right)}{\sum_{k\varepsilon S_{i}}\text{exp}\left(\mu v_{k}\right)}\;$$

In this paper, we use the mixed logit model (MIXL) (also known as the random parameters model), now common in estimating choice data (Hall et al., 2004; Hole, 2007; Hensher et al., 2005). Unlike standard logit models, MIXL allows for random taste variations, unrestricted substitution patterns, and correlations in unobserved factors that relax the IIA assumption (Campbell et al., 2006; Train, 2009; McFadden and Train, 2000). Therefore, we use the MIXL to understand farmers’ preferences for selected maize variety traits. In Eq. (6) below, the MIXL, the utility derived by farmer i from choosing maize variety j on choice occasion t is given by

(6) $$U_{ijt}=\left(\beta +\eta_{i}\right)x_{ijt}+\varepsilon_{ij}\;n=1,\ldots .N;j=1,\ldots J;t=1,\ldots T$$

where β is the vector of mean attribute utility weights in the population, and $\eta_{i}$ is the vector representing person $i^{\prime }s$ specific deviation from the mean. The random error term $\varepsilon_{ijt}$ is still assumed to be an IID extreme value. Following McFadden and Train (2000), the $\eta_{i}$ can be specified to take any distribution: normal, log-normal, or triangular. Although most applications use the multivariate normal, MVN (0, Ʃ), the price coefficient is sometimes assumed to be log-normal to impose an intuitive sign restriction (Fiebig et al., 2010; Train, 2009). Note that in our case, we replace the price variable with yield.

Assuming that most farmers prefer to maximize yield, we then look at the amount of yield that farmers may be willing to “sacrifice” to obtain other non-yield traits, where such trade-offs are warranted. Yield therefore constitutes the basic *numeraire* against which other varietal traits can be evaluated. We therefore use a fixed coefficient for yield while assuming preference heterogeneity across respondents for the non-yield attributes. The use of yield as a cost variable can also be found in Silberg et al. (2020) and their reasoning is that yield is the key measure of food security (and therefore maize choice decisions) and maize grain can constitute a form of exchange in rural communities. In the absence of a correlation between the attributes, in Eq. (7), the MIXL model takes the following form:

(7) $$Y_{ijt}=\gamma Q_{ijt}+\alpha Z_{ijt}+\varepsilon_{ijt}$$

where Y is a binary decision variable that takes the value of 1 if farmer i chooses variety j in choice scenario t, and 0 otherwise. Here Q is the yield attribute, which was used in place of the commonly-used price variable, while Z is a vector of other non-yield maize variety attributes. The non-yield traits included time to maturity, shelf-life (storability), grain size, tip cover, grain weight, top-dressing requirements, drought tolerance, lodging resistance, and *Striga* resistance. A positive coefficient for γ and α implies a positive influence of yield and non-yield variety attributes on the selection of a particular variety. Estimation of Eq. (7) gives the mean of the coefficient and its standard deviation around the mean. Preference heterogeneity is considered to be present if the standard deviation is statistically significant. Therefore, we extend Eqs. (7) and (8) below by including interaction terms to understand better the role of socioeconomic factors in influencing farmers’ preferences:

(8) $$Y_{ijt}=\gamma Q_{ijt}+\alpha Z_{ijt}+\delta \left(Z_{\mathrm{ijt}}*x_{i}\right)+\varepsilon_{ijt}$$

where x is a vector of socioeconomic characteristics including the age and education level of the respondent, size of land under maize crop, and primary occupation of the household head. Estimation of Eqs. (7) and (8) follow the simulated maximum likelihood method as described by Hole (2007).

The estimates obtained from the two equations are used to compute the willingness to pay for the selected attributes. Given that we use yield as the *numeraire* for evaluating the selected traits, we calculate the willingness to “sacrifice” yield (WTSY) as a measure of value for the other non-yield attributes. We estimate the WTSY (Eq. (9)) in a manner analogous to WTP (see Hole and Kolstad, 2012):

(9) $$WTSY=\frac{\partial Q}{\partial Z_{j}}=-\frac{\alpha_{j}}{\gamma }$$

In addition to the WTSY estimates obtained from the MIXL regressions, we also computed the WTSY using data from the BDM experiments. Mean values obtained from the difference between the starting points and the switch points (WTSY) for each attribute are presented in the results sections that follow.

## 3 Results

### 3.1 Choice experiment results

Table 5 presents results for the WTSY disaggregated by sex (with interaction terms to take care of taste heterogeneity, the essence of the MIXL model), location, age, education, and the income source of the respondent (without interactions). The patterns being that any trait related to pre or post-harvest loss such as drought tolerance, *Striga* tolerance, and storability have the largest WTSY. Among women, the WTSY for drought tolerance, *Striga* tolerance, and storability respectively were nominally 1.7, 1.9 and 3.6 times that of male participants. The disaggregated model shows that the WTSY estimates changed when demographic controls are accounted for in the MIXL model^2^. The largest increase in WTSY was for the closed tip and low top-dressing requirement in the male subsample—from 7.6 and 3.1 in the sex-disaggregated models with no interaction terms, increasing to about 61 in both cases^3^.

**Table 5:** Mean WTSY^**A**^ as disaggregated by sex, location and other demographics.

Attributes	Disaggregated by sex only with interaction terms			Disaggregated by other demographics (not sex, no interaction terms) (2)								
				County			Age		Education		Income source	
	Pooled	Male	Female	Kakamega	Siaya	Busia	≤35	>35	Post-primary	Primary	Non-Agric	Agric
Experiment A												
Matures in 3 months or less	4.84	1.73	5.60	1.33	1.74	1.36	1.84	1.30	1.04	1.82	1.47	1.46
Storability	19.66	8.80	32.06	8.75	9.62	8.13	12.12	7.42	5.91	9.76	10.55	7.91
Medium grains	-2.37	-1.28	-0.10	1.06	0.73	0.49	0.33	0.91	1.15	0.50	0.85	0.63
Large grains	0.04	1.28	-1.48	1.59	0.23	1.07	0.72	1.`29	1.68	0.92	1.93	1.03

Experiment B												
Closed tip	8.13	60.64	8.31	6.77	8.96	8.73	8.65	7.24	6.20	8.52	10.26	6.67
Heavy weight	6.15	4.31	7.04	4.44	2.63	3.76	4.69	3.27	3.28	4.06	3.74	4.17
Low top-dressing requirement	5.54	60.91	3.89	1.85	3.75	3.73	2.09	2.97	1.62	2.97	2.30	2.54

Experiment C												
Drought tolerant	10.27	7.63	13.04	9.88	9.76	9.65	21.15	7.67	10.73	9.71	11.36	9.82
Lodging resistant	4.25	3.27	4.32	7.01	2.29	5.73	6.74	3.85	4.66	4.14	3.82	4.77
*Striga* resistant	8.12	6.26	12.19	9.49	6.41	10.41	16.87	6.66	9.41	8.03	9.20	8.26

^**A**^ Recall from Eq. (9) that $WTSY=\frac{\partial Q}{\partial Z_{j}}=-\frac{\alpha_{j}}{\gamma }$ is unitless, measuring the “rate of trade” between the traits by a respondent as explained by her preferences.

### 3.2 Results from the BDM experiment

The BDM results (Table 6) show that the WTSY estimates among female and male participants were statistically indistinguishable. This is unsurprising because the basic notions of a good variety such as drought tolerance, high yield and good grain qualities should be universally desirable among men and women. The CE results (having accounted for heterogeneity from the MIXL model) show noticeable differences between men and women (Table 7). Therefore, while both men and women want the same things in their maize varieties, women seem to be willing to make slightly larger yield sacrifices in favor of tolerance to drought and *Striga* as well as for good storability^4^.

**Table 6:** Comparison of WTSY for between male and female respondents—BDM data.

Trait	Male	Female	difference	St-Err	t-value	p-value
Drought tolerant	5.74	5.931	-.191	.413	-.45	.645
*Striga* resistance	6.878	6.32	.559	.423	1.3	.188
Low fertilizer requirement	5.474	5.208	.266	.44	.6	.546
Lodging resistant	6.122	5.806	.316	.425	.75	.458
Early maturity	5.052	4.646	.406	.433	.95	.348
Closed tip	6.503	5.979	.523	.426	1.25	.22
Storability	6.676	6.542	.135	.423	.3	.751
Heavy grains	5.59	5.333	.257	.418	.6	.54
Large grains	4.925	4.417	.508	.426	1.2	.234

**Table 7:** Pairwise comparisons of WTSY between traits and based on the sex of respondent, Kenya choice experiment data 2017.

Traits	Observ 1	Observ 2	Mean 1	Mean 2	difference	St-Err	t-value
**Experiment A**							
Early maturity–Good for storage	325	-	4.776	19.954	-15.178∗∗∗	.24	-63.1
Early maturity–Medium size grains	325	-	4.776	-2.367	7.143∗∗∗	.204	35.05
Early maturity–Large grains	325	-	4.776	.036	4.74∗∗∗	.203	23.3
Good for storage–Medium grains	325	-	19.954	-2.367	22.321∗∗∗	.12	187.15
Good for storage–Large grains	325	-	19.954	.036	19.918∗∗∗	.12	166.85

	**Male vs Female WTSY pairwise tests**						
			Male	Female			

Early maturity (male–female)	143	182	1.754	5.431	-3.676∗∗∗	.325	-11.3
Storability (male–female)	143	182	8.73	31.581	-22.851∗∗∗	.905	-25.25
Medium size grains (male–female)	143	182	-1.351	-.041	-1.311∗∗∗	.054	-24.3
Large grains (male–female)	143	182	1.314	-1.502	2.816∗∗∗	.079	35.7

**Experiment B**							
Closed tip–Heavy grains	330	-	8.472	6.096	2.376∗∗∗	.455	5.2
Closed tip–Low top-dressing	330	-	8.472	5.382	3.09∗∗∗	.431	7.15
Heavy grain–Low top-dressing	330	-	6.096	5.382	.715∗∗	.324	2.2

	**Male vs Female WTSY pairwise tests**						
			Male	Female			

Closed tip (male–female)	147	183	60.501	7.973	52.528∗∗∗	1.216	43.2
Heavy grains (male–female)	147	183	6.099	6.959	-.861	1.185	-.75
Low top-dressing (male–female)	147	183	64.91	3.909	61.001∗∗∗	2.385	25.6

**Experiment C**							
Drought tolerant–Lodging resistant	320	-	10.2	4.258	5.942∗∗∗	.304	19.55
Drought tolerant–*Striga* resistant	320	-	10.2	8.326	1.873∗∗∗	.469	4
Lodging resistant–*Striga* resistant	320	-	4.258	8.326	-4.068∗∗∗	.321	-12.7

	**Male vs Female WTSY pairwise tests**						
			Male	Female			

Drought tolerant (male–female)	139	181	7.357	13.008	-5.651∗∗∗	.419	-13.45
Lodging resistant (male–female)	139	181	3.33	4.262	-.931∗∗∗	.307	-3.05
*Striga* resistant (male–female)	139	181	6.582	12.784	-6.202∗∗∗	.631	-9.85

The asterixes ∗∗∗, ∗∗ and ∗ means that the differences are (respectively) significant at: p < 0.01, p < 0.05, and p < 0.1.

### 3.3 Pairwise comparison tests from the CE results on WTSY

We carried out pairwise tests for the WTSY differences from the CE results. This was to test if the implied relative WTSY between traits were significant (Table 7). The pairwise comparisons show that the differences in WTSY between men and women were nearly all significant at the 5% confidence level. The only exception was the difference in WTSY for large grain sizes, where the difference between men and women was not significant. While these within-trait comparisons can only be made within experimental groups, they show that farmers placed a higher value on storability when compared to grain size or early maturity. The closed tip had a significantly higher WTSY than heavy grain or high nitrogen use efficiency (as perceived by low top-dressing requirement). The preference for heavy grains was slightly higher than that for nitrogen use efficiency. When compared with lodging resistance, *Striga* tolerance had a higher WTSY. Drought tolerance had a higher WTSY than lodging or *Striga* resistance.

In summary, taken together, the WTSY results in Tables 5, 6, and 7 show that among both men and women, WTSY for storability was nominally higher than that of drought tolerance. We say nominal because no pairwise comparison is possible, having included the two traits in two different experiments. Compared to men, women’s willingness to sacrifice yield for storability was three times higher. Women also valued storability—about six times more than 90-day maturity. Men seemed to place a higher value on the closed tip (seven times) than women. On the contrary, women valued drought tolerance and *Striga* resistance twice more than male farmers.

## 4 Conclusions

### 4.1 Implications for maize breeding programs, development, and policy

This study used an innovative measure of willingness to pay for maize traits—willingness to sacrifice yield (WTSY). By using demographic and choice experiment data from 1,288 respondents in mid-altitude maize growing areas of western Kenya and applying a mixed logit model to these data, the findings show that stress tolerance traits (against drought and *Striga* weed) have large WTSY and that characteristics related to storability also had comparable (and sometimes higher) WTSY. The results suggest that maize varieties with good storability (which is related to the flint type of gain) are highly valued. The WTSY for storability was nominally higher than that of drought tolerance, suggesting that grain characteristics that impart storability are prioritized by farmers. However, female farmers placed a higher value on storability (3.6 times) than males. Female farmers also valued storability about five times more than 90-day maturity. Male farmers, however, placed a much higher value on the closed tip—10 times more than female farmers. On the contrary, female farmers valued drought tolerance and *Striga* resistance twice more than male farmers. Among male farmers, closed tip and low top-dressing requirements were evaluated nearly equally. Low-top dressing requirement was valued about 20 times higher among male farmers than females.

For breeding research programs, these results suggest that maize genetic improvement programs should continue to focus on breeding for drought and *Striga* tolerance and, by implication, other biotic and abiotic stressors. Additionally, traits that relate to grain characteristics that impart better storability should be key in breeding research programs. These results imply that unless the risks of storage or pre-harvest losses are reduced or eliminated, the value of high yielding varieties can be diminished if they are susceptible to production stresses or the grain characteristics make them susceptible to storage pests. Therefore, for value chains development, the high value placed on storability suggests that investing in post-harvest technologies is an important development aspect. In future, stress tolerant and high yielding but somewhat low-storabilty varieties can still contribute to food security because they could still be acceptable to farmers if adavanced grain storage technologies are readily available. For policy departments responsible for maize breeding, we suggest that multi-criteria evaluations of new varieties be used to ensure that complementary stress tolerant traits and storability concerns are given optimal weighting in variety release criteria. Additionally, this information should be fed back to breeding programs in national institutes responsible for maize genetic improvement.

## Declarations

### Author contribution statement

Paswel Marenya: Conceived and designed the experiments; Performed the experiments; Analyzed and interpreted the data; Wrote the paper.

Rosina Wanyama: Analyzed and interpreted the data; Wrote the paper.

Solomon Alemu: Performed the experiments; Analyzed and interpreted the data; Wrote the paper.

Vincent Woyengo: Conceived and designed the experiments; Wrote the paper.

### Funding statement

This work was supported by the Bill & Melinda Gates Foundation [Grant INV 003439 and OPP1134248] and the United States Agency for International Development [Grant MTO 069033] through the Accelerating Genetic Gains for Maize and Wheat and Stress Tolerant Maize for Africa Projects.

### Data availability statement

Data will be made available on request.

### Declaration of interests statement

The authors declare no conflict of interest.

### Additional information

No additional information is available for this paper.

## Appendix A Supplementary data

Supplementary Online Materials.docx For the interested reader, we present supplementary material accompanying this paper in which we present more detailed descriptions, tables, and data to support the findings in this paper. Part 1 of the material describes the BDM process in more detail. Part 2 presents the details of the choice experiments, including sample cards. Part 3 presents the MXL regression results, showing the coefficient estimates of the covariates used in this study. Part 4 presents pairwise comparisons of the WTSY estimates from the BDM model results. Supplementary Online Materials.docx

Revised-BDM-Sessions-KE.pdf Revised-BDM-Sessions-KE.pdf

Kenya-Choce-Experiment-C.pdf Kenya-Choce-Experiment-C.pdf

Focus Group Disucssion Questions.docx Focus Group Disucssion Questions.docx

Kenya-Choce-Experiment-A.pdf Kenya-Choce-Experiment-A.pdf

Kenya-Choce-Experiment-B.pdf Kenya-Choce-Experiment-B.pdf

## Footnotes

1 Probability proportional to size (PPS) is a method of sampling from a finite population in which a size measure is available for each population unit before sampling and where the probability of selecting a unit is proportional to its size. Its use arises in two particular contexts: (i) multi-stage sampling and (ii) single-stage sampling of establishments.

2 For example, the WTSY among female participants increased from 1.5 (in the model without interactions) to 2.5 in the model with interaction terms. The impact of interaction terms is also visible for WTSY for drought tolerance and *Striga* resistance traits where the WTSY increased in the female subsample when interaction terms are included (see Table S3 in the Supplementary material for WTSY between men and women when demographic interactions are not accounted for).

3 This large WTSY is an artefact of the very low MIXL coefficient estimates for experiment B (disaggregated by sex and with interaction terms) where the two traits were featured. We take the low yield coefficient in this model with caution as we are not able to explain the exact interaction effects that produced it. In the final analysis we judge that the lower WTSY as estimated in the other models are more conservative and therefore reliable.

4 These results are in line with the above literature to capture the preferences of women since the notion of a single preference (utility function) within the household is not defensible (Alderman et al., 1995; Browning and Chiappori 1998; Udry 1996).
