# Supplementary material for: Trait preference trade-offs among maize farmers in western Kenya
Source: Heliyon. 2021 Mar 12;7(3):e06389. doi: 10.1016/j.heliyon.2021.e06389 (PMC7970324; doi:10.1016/j.heliyon.2021.e06389)
Supplement: Supplementary Online Materials.docx — For the interested reader, we present supplementary material accompanying this paper in which we present more detailed descriptions, tables, and data to support the findings in this paper. Part 1 of the material describes the BDM process in more detail. Part 2 presents the details of the choice experiments, including sample cards. Part 3 presents the MXL regression results, showing the coefficient estimates of the covariates used in this study. Part 4 presents pairwise comparisons of the WTSY estimates from the BDM model results. [file mmc1.docx]

**SUPPLEMENTARY MATERIALS**

Below are supplementary materials for the interested reader. In the supplementary materials accompanying this paper, we present more detailed descriptions, tables, and data to support the findings in this paper. Part 1 of the materials describes the BDM process in more detail. Part 2 presents the details of the choice experiments, including sample cards. Part 3 introduces the MXL regression results showing the coefficient estimates for the covariates used in this study. Part 4 presents pairwise comparisons on the WTSY estimates from the BDM model results.

**Part 1: The BDM process**

The BDMs involved presenting a series of thought experiments (auctions) to farmers to choose between pairs of varieties. One trait was used to compare two varieties at a time. To illustrate: regarding open or closed tip variety, one variety was described as open tip and the other closed tip. The farmer was taken through a series of comparisons of the yields between the two varieties starting with first step where both varieties are deemed to yield the same amount. The yield of the variety with the desirable trait (closed tip in this case) was lowered gradually while maintaining the yield of the variety with the less desirable trait). Each time the farmer was asked to choose which variety they would prefer. The point at which the farmer chose to switch to the variety with the less desirable trait was deemed to be their maximum willingness to pay (WTP) for the desirable trait. The starting point was 15 bags of 90 Kg/acre for both varieties. If the farmer switched to the open tip variety when the yield of the closed tip variety was 13 bags/acre*,* the WTP for the closed tip variety was calculated as the difference between the starting point and the switch point. In the case the WTP was 2 Kg/acre (15 minus 13 bags). The process was made incentive compatible (giving respondents incentive to reveal their true WTP) as per the principles of Becker, DeGroote and Mershack (1964). The actual procedure for the experiment and a sample session are shown in Table S1 below.

Prior to the experiments, extensive focus group discussions were held with farmers in the survey communities. Key traits identified during the discussions included: yield, maturity, storage/shelf-life, grain size, husk or tip cover, grain weight, top-dressing requirement, drought tolerance, and lodging and striga resistance. A summary of key traits, trait level and their distribution across the four experiments are presented in Table 3 in the main document. All the traits were used for both CEs and BDM. Figure S1 below shows a sample of choice experiment. Each respondent tackled twelve choice scenarios/sessions.

***Example of BDM procedure as implemented in the field***

1. In what follows, I would like to ask you which variety you prefer
2. You will notice that one of the varieties has the desirable trait
3. The other variety does not have the desirable trait
4. We will start by comparing the two varieties when the yields are the same
5. Then after that, you will state how much the yield of the good trait variety can go down before you decide to choose the variety which doesn’t have the good trait
6. After you reach this point (after you switch from your original choice to the variety which does not have the good trait) we will draw from a secret list the yields that range from 15bags per acre to 2bags per acre,
7. If you pick a yield that is equal the yield you stated you will earn points from this yield (1 bag=1 point). At the end of the game 1 point=ksh 15
8. If you pick a yield that is lower or greater than what you stated, you will earn zero points
9. Therefore, it is in your best interest to state the accurate yield reduction that you would be willing to tolerate in real life.
   - To repeat, it is better to say what you really think is the true yeild you would sacrifice for the good variety in real life

**Example**

1. Example: I have two varieties, one produces a lot of long leaves which can be used for livestock feed after harvesting. The other produces short and few leaves.
2. Let me ask you this:
3. Suppose the variety with short and few leaves yielded 15 bags per acre and the one with long and many leaves yielded 15 bags per acre. Which one do you prefer?
4. What is the lowest level of the yield of long and many leaves can go before you decided to choose the variety with short and few leaves?
5. Ok, I can see that you don’t want to go below xx bags per acre, meaning after xx bags/acre, you would rather have the variety with few and short leaves which has a yield of 15 bags per acres.
6. Now let us pick the secret yields from this envelope, if the yield from this envelop is equal to xx you will have ksh 3 times the yield that you stated.
7. Now pick.
8. Ok you picked yy bags/acre. Because yy is equal to xx bags per acre, now you have 3 ksh times the yield that you stick for it. (for practice)
9. We will now do 9 games like above and then we will choose one of the nine randomly. Then we will draw a secret yield. If the secret yield is equal, you will get the yield that you stated times ksh 15. Please note that 1bags/acre has one point which is equivalent to ksh15.
10. For example again: if you switched on 11 bags/acre, and the secret yield you pick is 11bags/acre you will get 11 points (1 bags/acre=1 point). Each point can earn you ksh 15 which is ksh 165. Do you have any question? Ok, let us start

Table S1: Sample BDM session

| **I would like to ask you about the following two maize varieties, one the TIP OR HUSK COVER is CLOSED and the other TIP OR HUSK COVER is OPEN: BDM H-L SESSION 4** | | | |
| --- | --- | --- | --- |
| LINE | Variety 1 | Variety 2 | |
| 1 | Suppose the one that has **TIP OR HUSK COVER OPEN** yielded **15 bags/acre** | AND The one that has T**IP OR HUSK COVER CLOSED** yielded **15 bags/acre** | Which would you choose? |
| 2 | Suppose the one that has **TIP OR HUSK COVER OPEN** yielded **15 bags/acre** | AND The one that has T**IP OR HUSK COVER CLOSED** yielded **14 bags/acre** | Which would you choose? |
| 3 | Suppose the one that has **TIP OR HUSK COVER OPEN** yielded **15 bags/acre** | AND The one that has T**IP OR HUSK COVER CLOSED** yielded **13 bags/acre** | Which would you choose? |
| 4 | Suppose the one that has **TIP OR HUSK COVER OPEN** yielded **15 bags/acre** | AND The one that has T**IP OR HUSK COVER CLOSED** yielded **12 bags/acre** | Which would you choose? |
| 5 | Suppose the one that has **TIP OR HUSK COVER OPEN** yielded **15 bags/acre** | AND The one that has T**IP OR HUSK COVER CLOSED** yielded **11 bags/acre** | Which would you choose? |
| 6 | Suppose the one that has **TIP OR HUSK COVER OPEN** yielded **15 bags/acre** | AND The one that has T**IP OR HUSK COVER CLOSED** yielded **10 bags/acre** | Which would you choose? |
| 7 | Suppose the one that has **TIP OR HUSK COVER OPEN** yielded **15 bags/acre** | AND The one that has T**IP OR HUSK COVER CLOSED** yielded **9 bags/acre** | Which would you choose? |
| 8 | Suppose the one that has **TIP OR HUSK COVER OPEN** yielded **15 bags/acre** | AND The one that has T**IP OR HUSK COVER CLOSED** yielded **8 bags/acre** | Which would you choose? |
| 9 | Suppose the one that has **TIP OR HUSK COVER OPEN** yielded **15 bags/acre** | AND The one that has T**IP OR HUSK COVER CLOSED** yielded **7 bags/acre** | Which would you choose? |
| 10 | Suppose the one that has **TIP OR HUSK COVER OPEN** yielded **15 bags/acre** | AND The one that has T**IP OR HUSK COVER CLOSED** yielded **6 bags/acre** | Which would you choose? |
| 11 | Suppose the one that has **TIP OR HUSK COVER OPEN** yielded **15 bags/acre** | AND The one that has T**IP OR HUSK COVER CLOSED** yielded **5 bags/acre** | Which would you choose? |
| 12 | Suppose the one that has **TIP OR HUSK COVER OPEN** yielded **15 bags/acre** | AND The one that has T**IP OR HUSK COVER CLOSED** yielded **4 bags/acre** | Which would you choose? |
| 13 | Suppose the one that has **TIP OR HUSK COVER OPEN** yielded **15 bags/acre** | AND The one that has T**IP OR HUSK COVER CLOSED** yielded **3 bags/acre** | Which would you choose? |
| 14 | Suppose the one that has **TIP OR HUSK COVER OPEN** yielded **15 bags/acre** | AND The one that has T**IP OR HUSK COVER CLOSED** yielded **2 bags/acre** | Which would you choose? |

*Notes*: Here, the yield of the variety with the desirable trait (in this case T**IP OR HUSK COVER CLOSED**) was lowered gradually while maintaining the yield of the variety with the less desirable trait (**TIP OR HUSK COVER OPEN**). Each time the farmer was asked to choose which variety they would prefer. The point at which the farmer chose to switch to the variety with the less desirable trait was deemed to be their maximum willingness to pay (WTP) for the desirable trait.

**Part 2 Sample CE session**

After a detailed explanation of the difference between traits in the two varieties in question, the respondent is asked to choose one preferred variety i.e. either variety one (*AINA YA MAHINDI - Ya Kwanza*) or variety two (*AINA YA MAHINDI - Ya Pili*). In **Session 3** below for instance, variety one has a maximum yield of 6 bags (90kg) per acre, bigger grains, matures in more than 3 months with a longer shelf life, while variety two has a maximum yield of 10 -15 bags (90kg) per acre, smaller grains, matures in less than 3 months with a shorter shelf life. Each respondent was made to select one preferred variety in 12 such sessions. The experiment was designed using NGENE software.


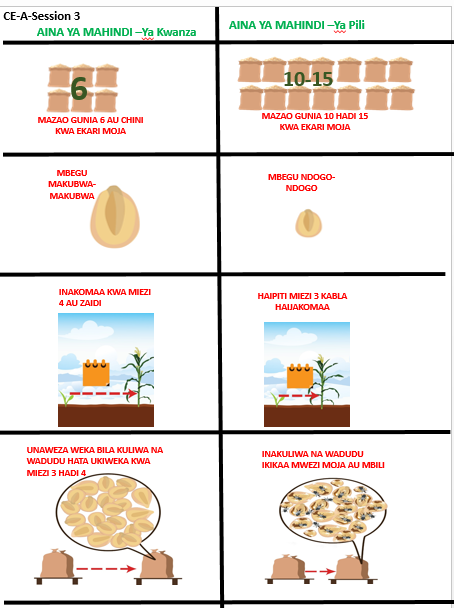


Figure S1: Sample choice card used in Experiment A, session 3

Part 3: Mixed logit regression results

***Coefficient estimates for yield compared to other traits:*** The basic mixed logit (MIXL) results are presented in Table 5. In experiment A, storability had the largest coefficient followed by early maturity. Preference for medium sized grains compared to small sized grains had the smallest coefficient. In Experiments B, closed tip had the largest coefficient. In experiment C, drought tolerance had the largest coefficient estimate followed by striga tolerance. In all the three CEs, yield as a trait did not have the largest coefficient. This does not reflect the low priority placed on yield, but that farmers evaluate the yield potential of any variety in the context of complementary traits such as storability, pre-harvest losses or tolerance to stressors. The WTSY provides a parsimonious yet intuitive measure of how farmers evaluate the competing traits.

Table 5: Mixed logit results disaggregated by sex of the respondent

| Variables | **Pooled** | |  | **Male respondent** | |  | **Female respondent** | |
| --- | --- | --- | --- | --- | --- | --- | --- | --- |
|  | **Coeff.** | **SD** |  | **Coeff** | **SD** |  | **Coeff** | **SD** |
| **Experiment A** |  |  |  |  |  |  |  |  |
| Yield | 0.479*** |  |  | 0.430*** |  |  | 0.522*** |  |
|  | (0.054) |  |  | (0.081) |  |  | (0.074) |  |
| Matures in 3 months or less | 0.737*** | 1.274*** |  | 0.758*** | 1.445*** |  | 0.790*** | 1.131*** |
|  | (0.122) | (0.161) |  | (0.188) | (0.221) |  | (0.165) | (0.218) |
| Good for storage | 4.271*** | 2.146*** |  | 4.360*** | 2.426*** |  | 4.153*** | 2.125*** |
|  | (0.238) | (0.187) |  | (0.377) | (0.250) |  | (0.279) | (0.276) |
| Medium size grain | 0.370*** | 0.339** |  | 0.589*** | 0.544** |  | 0.182 | 0.008 |
|  | (0.112) | (0.172) |  | (0.178) | (0.247) |  | (0.146) | (0.410) |
| Big grain size | 0.536*** | 1.299*** |  | 0.755*** | 1.249*** |  | 0.394* | 1.447*** |
|  | (0.168) | (0.142) |  | (0.241) | (0.243) |  | (0.231) | (0.207) |
|  |  |  |  |  |  |  |  |  |
| Observations | 7,800 | 7,800 |  | 3,432 | 3,432 |  | 4,368 | 4,368 |
| chi2 | 530.3 | 530.3 |  | 265.2 | 265.2 |  | 268.8 | 268.8 |
| Log Likelihood | -1220 | -1220 |  | -547.8 | -547.8 |  | -664.8 | -664.8 |
| **Experiment B** |  |  |  |  |  |  |  |  |
| Yield | 0.272*** |  |  | 0.307*** |  |  | 0.255*** |  |
|  | (0.027) |  |  | (0.042) |  |  | (0.036) |  |
| Closed tip | 2.142*** | 1.631*** |  | 2.323*** | 1.879*** |  | 1.942*** | 1.735*** |
|  | (0.131) | (0.141) |  | (0.207) | (0.236) |  | (0.158) | (0.184) |
| Heavy weight | 1.077*** | 1.282*** |  | 1.015*** | -1.275*** |  | 1.139*** | 1.275*** |
|  | (0.105) | (0.104) |  | (0.160) | (0.148) |  | (0.141) | (0.129) |
| Low top-dressing requirement | 0.737*** | 1.063*** |  | 0.939*** | 1.225*** |  | 0.711*** | 1.098*** |
|  | (0.083) | (0.089) |  | (0.139) | (0.143) |  | (0.112) | (0.123) |
|  |  |  |  |  |  |  |  |  |
| Observations | 7,920 | 7,920 |  | 3,528 | 3,528 |  | 4,392 | 4,392 |
| chi2 | 619.1 | 619.1 |  | 308.3 | 308.3 |  | 320.6 | 320.6 |
| Log Likelihood | -1809 | -1809 |  | -785.4 | -785.4 |  | -1016 | -1016 |
| **Experiment C** |  |  |  |  |  |  |  |  |
| Yield | 0.245*** |  |  | 0.263*** |  |  | 0.234*** |  |
|  | (0.034) |  |  | (0.050) |  |  | (0.046) |  |
| Drought tolerant | 2.406*** | 1.708*** |  | 2.244*** | 1.530*** |  | 2.618*** | 1.857*** |
|  | (0.147) | (0.130) |  | (0.220) | (0.164) |  | (0.212) | (0.191) |
| Lodging resistant | 1.099*** | 0.880*** |  | 1.064*** | -0.626*** |  | 1.168*** | -1.067*** |
|  | (0.102) | (0.149) |  | (0.140) | (0.239) |  | (0.149) | (0.179) |
| Striga resistant | 2.021*** | 1.452*** |  | 2.090*** | 1.663*** |  | 1.963*** | 1.463*** |
|  | (0.133) | (0.130) |  | (0.214) | (0.207) |  | (0.176) | (0.180) |
|  |  |  |  |  |  |  |  |  |
| Observations | 7,680 | 7,680 |  | 3,336 | 3,336 |  | 4,344 | 4,344 |
| chi2 | 479.7 | 479.7 |  | 216.3 | 216.3 |  | 274.0 | 274.0 |
| Log Likelihood | -1550 | -1550 |  | -695.5 | -695.5 |  | -844.9 | -844.9 |

Standard errors in parentheses; *** p<0.01, ** p<0.05, * p<0.1; SD, standard deviation

In Table 6, we also estimated the basic models but disaggregated by location (district or county of survey), age, education and source of income. We chose these three variables as the basis for disaggregation because they proxy for some of the most critical biophysical, demographic and economic determinants of maize variety choices (Kassie *et al*., 2017; Langyintuo and Mungoma, 2008; Salasya *et al*., 2007). The *county* of residence of the respondent is reflective of the local agro-ecology and market access, factors that will shape farmers’ preferences for specific traits such as more weight being placed on drought tolerance in areas with less (erratic) rainfall.

Table 6: Mixed logit estimates disaggregated by location, age, education and income source of the respondent

| Variables | County | | | Age | | Education | | Income source | |  |
| --- | --- | --- | --- | --- | --- | --- | --- | --- | --- | --- |
|  | Kakamega | Siaya | Busia | <=35 | >35 | Post-primary | Primary | Non-Agric | Agric |  |
| **Experiment A** |  |  |  |  |  |  |  |  |  |  |
| Yield | 0.471*** | 0.517*** | 0.513*** | 0.386*** | 0.543*** | 0.681*** | 0.432*** | 0.407*** | 0.521*** |  |
|  | (0.098) | (0.103) | (0.090) | (0.088) | (0.071) | (0.119) | (0.063) | (0.097) | (0.066) |  |
| Matures in 3 months or less | 0.624*** | 0.903*** | 0.696*** | 0.712*** | 0.704*** | 0.710** | 0.785*** | 0.598*** | 0.761*** |  |
|  | (0.188) | (0.256) | (0.230) | (0.200) | (0.156) | (0.278) | (0.134) | (0.200) | (0.149) |  |
| Good for storage | 4.120*** | 4.978*** | 4.169*** | 4.678*** | 4.028*** | 4.023*** | 4.214*** | 4.299*** | 4.121*** |  |
|  | (0.372) | (0.520) | (0.413) | (0.444) | (0.273) | (0.478) | (0.254) | (0.417) | (0.284) |  |
| Medium grains | 0.500** | 0.377 | 0.253 | 0.129 | 0.492*** | 0.784*** | 0.215 | 0.345* | 0.330** |  |
|  | (0.205) | (0.242) | (0.187) | (0.184) | (0.141) | (0.237) | (0.135) | (0.209) | (0.132) |  |
| Big grains | 0.747*** | 0.118 | 0.548* | 0.277 | 0.698*** | 1.144*** | 0.399** | 0.786*** | 0.535*** |  |
|  | (0.283) | (0.341) | (0.280) | (0.278) | (0.213) | (0.403) | (0.195) | (0.298) | (0.205) |  |
|  |  |  |  |  |  |  |  |  |  |  |
| Observations | 2,568 | 2,472 | 2,760 | 3,096 | 4,704 | 1,776 | 6,024 | 2,544 | 5,256 |  |
| chi2 | 133.6 | 202.1 | 207.3 | 214.6 | 315.0 | 132.5 | 390.6 | 167.4 | 358.7 |  |
| LL | -389.0 | -367.3 | -452.9 | -421.5 | -791.8 | -322.4 | -892.6 | -377.9 | -841.2 |  |
| **Experiment B** |  |  |  |  |  |  |  |  |  |  |
| Yield | 0.321*** | 0.225*** | 0.263*** | 0.270*** | 0.289*** | 0.353*** | 0.252*** | 0.293*** | 0.270*** |  |
|  | (0.041) | (0.048) | (0.057) | (0.049) | (0.034) | (0.054) | (0.032) | (0.055) | (0.032) |  |
| Closed tip | 2.174*** | 2.013*** | 2.300*** | 2.339*** | 2.090*** | 2.188*** | 2.151*** | 3.011*** | 1.799*** |  |
|  | (0.190) | (0.236) | (0.259) | (0.281) | (0.148) | (0.246) | (0.156) | (0.324) | (0.133) |  |
| Heavy weight | 1.425*** | 0.591*** | 0.990*** | 1.267*** | 0.943*** | 1.158*** | 1.025*** | 1.098*** | 1.125*** |  |
|  | (0.166) | (0.149) | (0.225) | (0.183) | (0.122) | (0.203) | (0.118) | (0.201) | (0.124) |  |
| Low top-dressing requirement | 0.595*** | 0.843*** | 0.983*** | 0.566*** | 0.857*** | 0.570*** | 0.749*** | 0.675*** | 0.685*** |  |
|  | (0.117) | (0.160) | (0.183) | (0.146) | (0.107) | (0.166) | (0.097) | (0.184) | (0.091) |  |
|  |  |  |  |  |  |  |  |  |  |  |
| Observations | 3,672 | 2,256 | 1,992 | 2,784 | 5,136 | 2,088 | 5,832 | 2,496 | 5,424 |  |
| chi2 | 277.7 | 186.5 | 172.1 | 270.6 | 389.3 | 143.0 | 503.7 | 264.4 | 377.7 |  |
| LL | -829.3 | -528.8 | -427.7 | -612.6 | -1172 | -466.3 | -1325 | -502.1 | -1288 |  |
| **Experiment C** |  |  |  |  |  |  |  |  |  |  |
| Yield | 0.166*** | 0.306*** | 0.282*** | 0.124** | 0.305*** | 0.232*** | 0.253*** | 0.241*** | 0.239*** |  |
|  | (0.054) | (0.056) | (0.068) | (0.061) | (0.042) | (0.063) | (0.040) | (0.065) | (0.039) |  |
| Drought tolerant | 1.640*** | 2.989*** | 2.718*** | 2.632*** | 2.342*** | 2.487*** | 2.456*** | 2.742*** | 2.348*** |  |
|  | (0.197) | (0.282) | (0.315) | (0.287) | (0.180) | (0.282) | (0.185) | (0.326) | (0.176) |  |
| Lodging resistant | 1.163*** | 0.701*** | 1.614*** | 0.839*** | 1.174*** | 1.080*** | 1.047*** | 0.921*** | 1.140*** |  |
|  | (0.167) | (0.150) | (0.220) | (0.149) | (0.129) | (0.180) | (0.118) | (0.184) | (0.113) |  |
| Striga resistant | 1.576*** | 1.962*** | 2.932*** | 2.099*** | 2.034*** | 2.181*** | 2.031*** | 2.221*** | 1.976*** |  |
|  | (0.201) | (0.228) | (0.329) | (0.230) | (0.172) | (0.246) | (0.163) | (0.289) | (0.155) |  |
|  |  |  |  |  |  |  |  |  |  |  |
| Observations | 1,968 | 3,216 | 2,496 | 2,856 | 4,824 | 2,280 | 5,400 | 2,400 | 5,280 |  |
| chi2 | 67.45 | 257.9 | 163.7 | 157.5 | 318.3 | 141.8 | 342.9 | 163.4 | 320.7 |  |
| LL | -459.4 | -606.2 | -457.0 | -515.4 | -1020 | -444.1 | -1103 | -461.8 | -1083 |  |

The table shows mean values with standard errors in parentheses. Standard deviation estimates shown in Table S2 in the Supplementary material; *** p<0.01, ** p<0.05, * p<0.1

Table S2: Standard deviation values from mixed logit model disaggregated by location, age, education and income source of the respondent

| Variables | County | | | Age | | Education | | Income source | |  |
| --- | --- | --- | --- | --- | --- | --- | --- | --- | --- | --- |
|  | Kakamega | Siaya | Busia | <=35 | >35 | Post-primary | Primary | Non-Agric | Agric |  |
| ***Experiment A*** |  |  |  |  |  |  |  |  |  |  |
| Matures in 3 months or less | 0.989*** | 1.705*** | 1.579*** | 1.104*** | 1.396*** | 1.729*** | 1.082*** | -1.133*** | 1.431*** |  |
|  | (0.260) | (0.366) | (0.279) | (0.244) | (0.191) | (0.326) | (0.159) | (0.247) | (0.203) |  |
| Good for storage | 1.771*** | 2.748*** | 2.374*** | 2.671*** | 1.912*** | 2.480*** | 2.179*** | 2.234*** | 2.254*** |  |
|  | (0.210) | (0.368) | (0.317) | (0.316) | (0.215) | (0.452) | (0.235) | (0.292) | (0.204) |  |
| Medium grains | 0.530** | 0.937*** | -0.365 | 0.199 | -0.335 | 0.474 | 0.640*** | -0.616** | -0.242 |  |
|  | (0.259) | (0.270) | (0.240) | (0.274) | (0.435) | (0.376) | (0.211) | (0.300) | (0.291) |  |
| Big grains | 1.140*** | 1.560*** | 1.585*** | -1.319*** | 1.277*** | 1.384*** | 1.392*** | -1.381*** | 1.237*** |  |
|  | (0.246) | (0.280) | (0.304) | (0.280) | (0.189) | (0.343) | (0.169) | (0.260) | (0.187) |  |
|  |  |  |  |  |  |  |  |  |  |  |
| ***Experiment B*** |  |  |  |  |  |  |  |  |  |  |
| Closed tip | 1.599*** | 1.783*** | 1.679*** | 2.182*** | 1.726*** | 1.633*** | 1.850*** | 2.312*** | 1.540*** |  |
|  | (0.174) | (0.264) | (0.277) | (0.251) | (0.171) | (0.271) | (0.152) | (0.361) | (0.156) |  |
| Heavy weight | 1.489*** | 0.854*** | 1.687*** | 1.499*** | 1.359*** | 1.256*** | 1.269*** | 1.312*** | 1.313*** |  |
|  | (0.162) | (0.178) | (0.257) | (0.195) | (0.128) | (0.171) | (0.104) | (0.195) | (0.117) |  |
| Low top-dressing requirement | 0.983*** | 1.145*** | 1.131*** | 1.224*** | 1.091*** | 1.134*** | 1.128*** | 1.587*** | 0.956*** |  |
|  | (0.111) | (0.155) | (0.208) | (0.171) | (0.105) | (0.182) | (0.111) | (0.201) | (0.104) |  |
|  |  |  |  |  |  |  |  |  |  |  |
| ***Experiment C*** |  |  |  |  |  |  |  |  |  |  |
| Drought tolerant | 1.094*** | 2.136*** | 1.708*** | 1.626*** | 1.736*** | 1.642*** | 1.671*** | 1.647*** | 1.633*** |  |
|  | (0.185) | (0.228) | (0.228) | (0.232) | (0.152) | (0.213) | (0.143) | (0.212) | (0.145) |  |
| Lodging resistant | 0.598** | -0.809*** | 1.122*** | 0.630** | 0.965*** | 0.781*** | 0.895*** | 0.942*** | -0.617*** |  |
|  | (0.250) | (0.241) | (0.323) | (0.308) | (0.164) | (0.237) | (0.163) | (0.267) | (0.228) |  |
| Striga resistant | 1.067*** | 1.544*** | 1.895*** | 1.285*** | 1.563*** | 1.200*** | 1.506*** | 1.722*** | 1.445*** |  |
|  | (0.193) | (0.212) | (0.271) | (0.222) | (0.154) | (0.212) | (0.156) | (0.269) | (0.149) |  |

Standard errors in parentheses; *** p<0.01, ** p<0.05, * p<0.1; the values are standard deviation parameters obtained from mixed logit models. The mean values for the corresponding models are shown in Table 6 in the main text. Significant values show the presence of preference heterogeneity among farmers for the specific attribute.

Table S3: Mean WTSY dis aggregated by sex (basic model)

| Attributes | Pooled | Male | Female |
| --- | --- | --- | --- |
| Experiment A |  |  |  |
| Matures in 3 months or less | 1.54 | 1.76 | 1.51 |
| Good for storage | 8.92 | 10.15 | 7.95 |
| Medium grains | 0.77 | 1.37 | 0.35 |
| Large grains | 1.12 | 1.76 | 0.75 |
| Experiment B |  |  |  |
| Closed tip | 7.87 | 7.56 | 7.62 |
| Heavy weight | 3.96 | 3.30 | 4.47 |
| Low top-dressing requirement | 2.71 | 3.06 | 2.79 |
| Experiment C |  |  |  |
| Drought tolerant | 9.80 | 8.55 | 11.17 |
| Lodging resistant | 4.48 | 4.05 | 4.98 |
| Striga resistant | 8.23 | 7.96 | 8.38 |

*Notes*: WTSY, willingness to sacrifice yield; the values are computed as a ratio of the coefficient of the attribute and the cost coefficient. In our case yield was used as the cost coefficient. The coefficients for the attributes are shown in Table 5 in the main text.

Part 4: Pairwise comparisons for the BDM WTSY results

Table S4: Comparison of WTSY for pooled sample – BDM data

| Traits | Mean 1 | Mean 2 | Difference† | t-value |
| --- | --- | --- | --- | --- |
| Drought tolerance – Early maturity | 5.827 | 4.867 | .959*** | 5 |
| Drought tolerance – Lodging resistant | 5.827 | 5.978 | -.151 | -.75 |
| Drought tolerance – Closed tip | 5.827 | 6.265 | -.438** | -2.1 |
| Drought tolerance – Striga resistant | 5.827 | 6.625 | -.798*** | -4.25 |
| Drought tolerance – Good storage | 5.827 | 6.615 | -.789*** | -3.65 |
| Drought tolerance – Heavy grain | 5.827 | 5.473 | .353 | 1.65 |
| Drought tolerance – Low fertilizer requirement | 5.827 | 5.354 | .473** | 2 |
| Drought tolerance – Big grains | 5.827 | 4.694 | 1.132*** | 4.85 |
| Early maturity – Lodging resistant | 4.867 | 5.978 | -1.11*** | -5.75 |
| Early maturity – Closed tip | 4.867 | 6.265 | -1.397*** | -6.15 |
| Early maturity – Striga resistant | 4.867 | 6.625 | -1.757*** | -8.35 |
| Early maturity – Good storage | 4.867 | 6.615 | -1.748*** | -7.1 |
| Early maturity – Heavy grain | 4.867 | 5.473 | -.606** | -2.6 |
| Early maturity – Low fertilizer requirement | 4.867 | 5.354 | -.486** | -2 |
| Early maturity – Big grains | 4.867 | 4.694 | .174 | .75 |
| Closed tip – Striga resistant | 6.265 | 6.625 | -.36* | -1.95 |
| Closed tip – Good storage | 6.265 | 6.615 | -.35* | -1.7 |
| Closed tip – Heavy grain | 6.265 | 5.473 | .792*** | 4 |
| Closed tip - Low fertilizer requirement | 6.265 | 5.354 | .912*** | 4.25 |
| Closed tip – Big grains | 6.265 | 4.694 | 1.571*** | 6.95 |
| Lodging resistant – Closed tip | 5.978 | 6.625 | -.647*** | -3.15 |
| Lodging resistant - Striga resistant | 5.978 | 6.615 | -.637*** | -2.95 |
| Lodging resistant - Good storage | 5.978 | 5.473 | .505** | 2.55 |
| Lodging resistant - Heavy grain | 5.978 | 5.354 | .625*** | 3.15 |
| Lodging resistant - Low fertilizer requirement | 5.978 | 4.694 | 1.284*** | 6.2 |
| Lodging resistant – Big grains | 5.978 | 6.265 | -.287 | -1.45 |
| Striga resistant – Good storage | 6.625 | 6.615 | .009 | .05 |
| Striga resistant -Heavy grains | 6.625 | 5.473 | 1.151*** | 5.65 |
| Striga resistant - Low fertilizer requirement | 6.625 | 5.354 | 1.271*** | 5.4 |
| Striga resistant – Big grains | 6.625 | 4.694 | 1.931*** | 8.65 |
| Good storage – Heavy grains | 6.615 | 5.473 | 1.142*** | 5.7 |
| Good storage – Low fertilizer requirement | 6.615 | 5.354 | 1.262*** | 5.8 |
| Good storage – Big grains | 6.615 | 4.694 | 1.921*** | 8.6 |
| Heavy grain - Low fertilizer requirement | 5.473 | 5.354 | .12 | .6 |
| Heavy grains – Big grains | 5.473 | 4.694 | .779*** | 4.05 |
| Low fertilizer requirement – Big grains | 5.354 | 4.694 | .659*** | 3.45 |

Difference significant at: *** p<0.01, ** p<0.05, * p<0.1; WTSY, mean willingness to sacrifice yield; †, the difference between Mean 1 and Mean 2 for the two traits in consideration.
