# Supplementary material for: Trait preference trade-offs among maize farmers in western Kenya
Source: Heliyon. 2021 Mar 12;7(3):e06389. doi: 10.1016/j.heliyon.2021.e06389 (PMC7970324; doi:10.1016/j.heliyon.2021.e06389)
Supplement: Revised-BDM-Sessions-KE.pdf [file mmc2.pdf]

I would like to ask you about the following two maize varieties, one is PLAIN TASTING (NOT TASTY) when boiled or roasted and the other TASTES VERY WELL (IS SWEET) when boiled or roasted:

	BDM SESSION 1		
LINE	Variety 1	Variety 2	
1	Suppose the **plain tasting** one yielded **7** quintals per timad	AND The **sweet tasting** one yielded **7** quintals per timad	Which would you choose?
2	Suppose the **plain tasting** one yielded **7** quintals per timad	AND The **sweet tasting** one yielded **6.5** quintals per timad	Which would you choose?
3	Suppose the **plain tasting** one yielded **7** quintals per timad	AND The **sweet tasting** one yielded **6** quintals per timad	Which would you choose?
4	Suppose the **plain tasting** one yielded **7** quintals per timad	AND The **sweet tasting** one yielded **5.5** quintals per timad	Which would you choose?
5	Suppose the **plain tasting** one yielded **7** quintals per timad	AND The **sweet tasting** one yielded **5** quintals per timad	Which would you choose?
6	Suppose the **plain tasting** one yielded **7** quintals per timad	AND The **sweet tasting** one yielded **4.5** quintals per timad	Which would you choose?
7	Suppose the **plain tasting** one yielded **7** quintals per timad	AND The **sweet tasting** one yielded **4** quintals per timad	Which would you choose?
8	Suppose the **plain tasting** one yielded **7** quintals per timad	AND The **sweet tasting** one yielded **3.5** quintals per timad	Which would you choose?
9	Suppose the **plain tasting** one yielded **7** quintals per timad	AND The **sweet tasting** one yielded **3** quintals per timad	Which would you choose?
10	Suppose the **plain tasting** one yielded **7** quintals per timad	AND The **sweet tasting** one yielded **2.5** quintals per timad	Which would you choose?
11	Suppose the **plain tasting** one yielded **7** quintals per timad	AND The **sweet tasting** one yielded **2** quintals per timad	Which would you choose?
12	Suppose the **plain tasting** one yielded **7** quintals per timad	AND The **sweet tasting** one yielded **1.5** quintals per timad	Which would you choose?
13	Suppose the **plain tasting** one yielded **7** quintals per timad	AND The **sweet tasting** one yielded **1** quintals per timad	Which would you choose?
14	Suppose the **plain tasting** one yielded **7** quintals per timad	AND The **sweet tasting** one yielded **0.5** quintals per timad	Which would you choose?

I would like to ask you about the following two maize varieties, one DROUGHT TOLERANT and the other IS NOT DROUGHT TOLERANT:

		BDM SESSION 2	
LINE	**Variety 1**	**Variety 2**	
1	Suppose the one which is **NOT drought** tolerant yielded **7** quintals per timad	AND The **DROUGHT TOLERANT** one yielded **7** quintals per timad	Which would you choose?
2	Suppose the one which is **NOT drought** tolerant yielded **7** quintals per timad	AND The **DROUGHT TOLERANT** one yielded **6.5** quintals per timad	Which would you choose?
3	Suppose the one which is **NOT drought** tolerant yielded **7** quintals per timad	AND The **DROUGHT TOLERANT** one yielded **6** quintals per timad	Which would you choose?
4	Suppose the one which is **NOT drought** tolerant yielded **7** quintals per timad	AND The **DROUGHT TOLERANT** one yielded **5.5** quintals per timad	Which would you choose?
5	Suppose the one which is **NOT drought** tolerant yielded **7** quintals per timad	AND The **DROUGHT TOLERANT** one yielded **5** quintals per timad	Which would you choose?
6	Suppose the one which is **NOT drought** tolerant yielded **7** quintals per timad	AND The **DROUGHT TOLERANT** one yielded **4.5** quintals per timad	Which would you choose?
7	Suppose the one which is **NOT drought** tolerant yielded **7** quintals per timad	AND The **DROUGHT TOLERANT** one yielded **4** quintals per timad	Which would you choose?
8	Suppose the one which is **NOT drought** tolerant yielded **7** quintals per timad	AND The **DROUGHT TOLERANT** one yielded **3.5** quintals per timad	Which would you choose?
9	Suppose the one which is **NOT drought** tolerant yielded **7** quintals per timad	AND The **DROUGHT TOLERANT** one yielded **3** quintals per timad	Which would you choose?
10	Suppose the one which is **NOT drought** tolerant yielded **7** quintals per timad	AND The **DROUGHT TOLERANT** one yielded **2.5** quintals per timad	Which would you choose?
11	Suppose the one which is **NOT drought** tolerant yielded **7** quintals per timad	AND The **DROUGHT TOLERANT** one yielded **2** quintals per timad	Which would you choose?
12	Suppose the one which is **NOT drought** tolerant yielded **7** quintals per timad	AND The **DROUGHT TOLERANT** one yielded **1.5** quintals per timad	Which would you choose?
13	Suppose the one which is **NOT drought** tolerant yielded **7** quintals per timad	AND The **DROUGHT TOLERANT** one yielded **1** quintals per timad	Which would you choose?
14	Suppose the one which is **NOT drought** tolerant yielded **7** quintals per timad	AND The **DROUGHT TOLERANT** one yielded **0.5** quintals per timad	Which would you choose?

I would like to ask you about the following two maize varieties, one IS LODGING RESISTANT and the other IS NOT lodging resistant:

		BDM SESSION 3	
LINE	Variety 1	Variety 2	
1	Suppose the one **NOT Lodging resistant** yielded **7** quintals per timad	AND The **LODGING RESISTANT** one yielded **7** quintals per timad	Which would you choose?
2	Suppose the one **NOT Lodging resistant** yielded **7** quintals per timad	AND The **LODGING RESISTANT** one yielded **6.5** quintals per timad	Which would you choose?
3	Suppose the one **NOT Lodging resistant** yielded **7** quintals per timad	AND The **LODGING RESISTANT** yielded **6** quintals per timad	Which would you choose?
4	Suppose the one **NOT Lodging resistant** yielded **7** quintals per timad	AND The **LODGING RESISTANT** one yielded **5.5** quintals per timad	Which would you choose?
5	Suppose the one **NOT Lodging resistant** yielded **7** quintals per timad	AND The **LODGING RESISTANT** one yielded **5** quintals per timad	Which would you choose?
6	Suppose the one **NOT Lodging resistant** yielded **7** quintals per timad	AND The **LODGING RESISTANT** one yielded **4.5** quintals per timad	Which would you choose?
7	Suppose the one **NOT Lodging resistant** yielded **7** quintals per timad	AND The **LODGING RESISTANT** yielded **4** quintals per timad	Which would you choose?
8	Suppose the one **NOT Lodging resistant** yielded **7** quintals per timad	AND The **LODGING RESISTANT** one yielded **3.5** quintals per timad	Which would you choose?
9	Suppose the one **NOT Lodging resistant** yielded **7** quintals per timad	AND The **LODGING RESISTANT** one yielded **3** quintals per timad	Which would you choose?
10	Suppose the one **NOT Lodging resistant** yielded **7** quintals per timad	AND The **LODGING RESISTANT** one yielded **2.5** quintals per timad	Which would you choose?
11	Suppose the one **NOT Lodging resistant** yielded **7** quintals per timad	AND The **LODGING RESISTANT** one yielded **2** quintals per timad	Which would you choose?
12	Suppose the one **NOT Lodging resistant** yielded **7** quintals per timad	AND The **LODGING RESISTANT** one yielded **1.5** quintals per timad	Which would you choose?
13	Suppose the one **NOT Lodging resistant** yielded **7** quintals per timad	AND The **LODGING RESISTANT** one yielded **1** quintals per timad	Which would you choose?
14	Suppose the one **NOT Lodging resistant** yielded **7** quintals per timad	AND The **LODGING RESISTANT** one yielded **0.5** quintals per timad	Which would you choose?

I would like to ask you about the following two maize varieties, one MATURES IN 4 MONTHS and the other MATURES IN 3 MONTHS:

			BDM H-L SESSION 4
LINE	Variety 1	Variety 2	
1	Suppose the one that **MATURES IN 4 MONTHS** yielded **7** quintals per timad	AND The one that **MATURES IN 3 MONTHS** yielded **7** quintals per timad	Which would you choose?
2	Suppose the one that **MATURES IN 4 MONTHS** yielded **7** quintals per timad	AND The one that **MATURES IN 3 MONTHS** yielded **6.5** quintals per timad	Which would you choose?
3	Suppose the one that **MATURES IN 4 MONTHS** yielded **7** quintals per timad	AND The one that **MATURES IN 3 MONTHS** yielded **6** quintals per timad	Which would you choose?
4	Suppose the one that **MATURES IN 4 MONTHS** yielded **7** quintals per timad	AND The one that **MATURES IN 3 MONTHS** yielded **5.5** quintals per timad	Which would you choose?
5	Suppose the one that **MATURES IN 4 MONTHS** yielded **7** quintals per timad	AND The one that **MATURES IN 3 MONTHS** yielded **5** quintals per timad	Which would you choose?
6	Suppose the one that **MATURES IN 4 MONTHS** yielded **7** quintals per timad	AND The one that **MATURES IN 3 MONTHS** yielded **4.5** quintals per timad	Which would you choose?
7	Suppose the one that **MATURES IN 4 MONTHS** yielded **7** quintals per timad	AND The one that **MATURES IN 3 MONTHS** yielded **4** quintals per timad	Which would you choose?
8	Suppose the one that **MATURES IN 4 MONTHS** yielded **7** quintals per timad	AND The one that **MATURES IN 3 MONTHS** yielded **3.5** quintals per timad	Which would you choose?
9	Suppose the one that **MATURES IN 4 MONTHS** yielded **7** quintals per timad	AND The one that **MATURES IN 3 MONTHS** yielded **3** quintals per timad	Which would you choose?
10	Suppose the one that **MATURES IN 4 MONTHS** yielded **7** quintals per timad	AND The one that **MATURES IN 3 MONTHS** yielded **2.5** quintals per timad	Which would you choose?
11	Suppose the one that **MATURES IN 4 MONTHS** yielded **7** quintals per timad	AND The one that **MATURES IN 3 MONTHS** yielded **2** quintals per timad	Which would you choose?
12	Suppose the one that **MATURES IN 4 MONTHS** yielded **7** quintals per timad	AND The one that **MATURES IN 3 MONTHS** yielded **1.5** quintals per timad	Which would you choose?
13	Suppose the one that **MATURES IN 4 MONTHS** yielded **7** quintals per timad	AND The one that **MATURES IN 3 MONTHS** yielded **1** quintals per timad	Which would you choose?
14	Suppose the one that **MATURES IN 4 MONTHS** yielded **7** quintals per timad	AND The one that **MATURES IN 3 MONTHS** yielded **0.5** quintals per timad	Which would you choose?

I would like to ask you about the following two maize varieties, one the TIP OR HUSK COVER is OPEN and the other TIP OR HUSK COVER is CLOSED:

		**BDM H-L SESSION 5**	
**LINE**	**Variety 1**	**Variety 2**	
1	Suppose the one that has **TIP OR HUSK COVER OPEN** yielded **7** quintals per timad	AND The one that has **TIP OR HUSK COVER CLOSED** yielded **7** quintals per timad	Which would you choose?
2	Suppose the one that has **TIP OR HUSK COVER OPEN** yielded **7** quintals per timad	AND The one that has **TIP OR HUSK COVER CLOSED** yielded **6.5** quintals per timad	Which would you choose?
3	Suppose the one that has **TIP OR HUSK COVER OPEN** yielded **7** quintals per timad	AND The one that has **TIP OR HUSK COVER CLOSED** yielded **6** quintals per timad	Which would you choose?
4	Suppose the one that has **TIP OR HUSK COVER OPEN** yielded **7** quintals per timad	AND The one that has **TIP OR HUSK COVER CLOSED** yielded **5.5** quintals per timad	Which would you choose?
5	Suppose the one that has **TIP OR HUSK COVER OPEN** yielded **7** quintals per timad	AND The one that has **TIP OR HUSK COVER CLOSED** yielded **5** quintals per timad	Which would you choose?
6	Suppose the one that has **TIP OR HUSK COVER OPEN** yielded **7** quintals per timad	AND The one that has **TIP OR HUSK COVER CLOSED** yielded **4.5** quintals per timad	Which would you choose?
7	Suppose the one that has **TIP OR HUSK COVER OPEN** yielded **7** quintals per timad	AND The one that has **TIP OR HUSK COVER CLOSED** yielded **4** quintals per timad	Which would you choose?
8	Suppose the one that has **TIP OR HUSK COVER OPEN** yielded **7** quintals per timad	AND The one that has **TIP OR HUSK COVER CLOSED** yielded **3.5** quintals per timad	Which would you choose?
9	Suppose the one that has **TIP OR HUSK COVER OPEN** yielded **7** quintals per timad	AND The one that has **TIP OR HUSK COVER CLOSED** yielded **3** quintals per timad	Which would you choose?
10	Suppose the one that has **TIP OR HUSK COVER OPEN** yielded **7** quintals per timad	AND The one that has **TIP OR HUSK COVER CLOSED** yielded **2.5** quintals per timad	Which would you choose?
11	Suppose the one that has **TIP OR HUSK COVER OPEN** yielded **7** quintals per timad	AND The one that has **TIP OR HUSK COVER CLOSED** yielded **2** quintals per timad	Which would you choose?
12	Suppose the one that has **TIP OR HUSK COVER OPEN** yielded **7** quintals per timad	AND The one that has **TIP OR HUSK COVER CLOSED** yielded **1.5** quintals per timad	Which would you choose?
13	Suppose the one that has **TIP OR HUSK COVER OPEN** yielded **7** quintals per timad	AND The one that has **TIP OR HUSK COVER CLOSED** yielded **1** quintals per timad	Which would you choose?
14	Suppose the one that has **TIP OR HUSK COVER OPEN** yielded **7** quintals per timad	AND The one that has **TIP OR HUSK COVER CLOSED** yielded **0.5** quintals per timad	Which would you choose?

I would like to ask you about the following two maize varieties, one IS RESISTANT to rust or the most common local maize pest and the IS NOT resistant:

		BDM H-L SESSION 6	
LINE	Variety 1	Variety 2	
1	Suppose the one that is **NOT** resistant to rust or local pest yielded **7** quintals per timad	AND The one that is **RESISTANT** to rust or local pest yielded **7** quintals per timad	Which would you choose?
2	Suppose the one that is **NOT** resistant to rust or local pest yielded **7** quintals per timad	AND The one that is **RESISTANT** to rust or local pest yielded **6.5** quintals per timad	Which would you choose?
3	Suppose the one that is **NOT** resistant to rust or local pest yielded **7** quintals per timad	AND The one that is **RESISTANT** to rust or local pest yielded **6** quintals per timad	Which would you choose?
4	Suppose the one that is **NOT** resistant to rust or local pest yielded **7** quintals per timad	AND The one that is **RESISTANT** to rust or local pest yielded **5.5** quintals per timad	Which would you choose?
5	Suppose the one that is **NOT** resistant to rust or local pest yielded **7** quintals per timad	AND The one that is **RESISTANT** to rust or local pest yielded **5** quintals per timad	Which would you choose?
6	Suppose the one that is **NOT** resistant to rust or local pest yielded **7** quintals per timad	AND The one that is **RESISTANT** to rust or local pest yielded **4.5** quintals per timad	Which would you choose?
7	Suppose the one that is **NOT** resistant to rust or local pest yielded **7** quintals per timad	AND The one that is **RESISTANT** to rust or local pest yielded **4** quintals per timad	Which would you choose?
8	Suppose the one that is **NOT** resistant to rust or local pest yielded **7** quintals per timad	AND The one that is **RESISTANT** to rust or local pest yielded **3.5** quintals per timad	Which would you choose?
9	Suppose the one that is **NOT** resistant to rust or local pest yielded **7** quintals per timad	AND The one that is **RESISTANT** to rust or local pest yielded **3** quintals per timad	Which would you choose?
10	Suppose the one that is **NOT** resistant to rust or local pest yielded **7** quintals per timad	AND The one that is **RESISTANT** to rust or local pest yielded **2.5** quintals per timad	Which would you choose?
11	Suppose the one that is **NOT** resistant to rust or local pest yielded **7** quintals per timad	AND The one that is **RESISTANT** to rust or local pest yielded **2** quintals per timad	Which would you choose?
12	Suppose the one that is **NOT** resistant to rust or local pest yielded **7** quintals per timad	AND The one that is **RESISTANT** to rust or local pest yielded **1.5** quintals per timad	Which would you choose?
13	Suppose the one that is **NOT** resistant to rust or local pest yielded **7** quintals per timad	AND The one that is **RESISTANT** to rust or local pest yielded **1** quintals per timad	Which would you choose?
14	Suppose the one that is **NOT** resistant to rust or local pest yielded **7** quintals per timad	AND The one that is **RESISTANT** to rust or local pest yielded **0.5** quintals per timad	Which would you choose?
