# Supplementary material for: Trait preference trade-offs among maize farmers in western Kenya
Source: Heliyon. 2021 Mar 12;7(3):e06389. doi: 10.1016/j.heliyon.2021.e06389 (PMC7970324; doi:10.1016/j.heliyon.2021.e06389)

CE-C-Session 1

AINA YA MAHINDI –Ya Kwanza

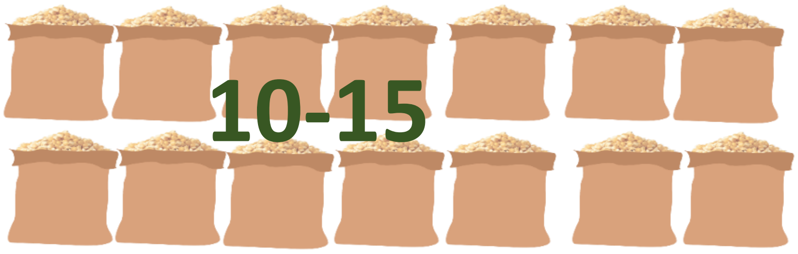

MAZAO GUNIA 10 HADI 15
KWA EKARI MOJA

INA-VUMILIA UKAME, INAZLISHA
KIASI HATA KUKITOKEA UKAME
KATI-KATI YA MSIMU

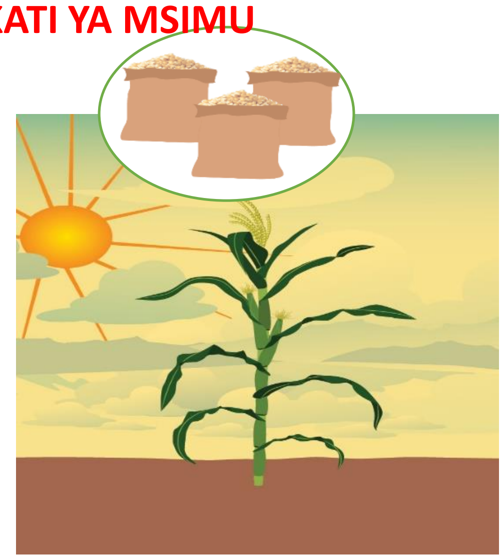

LINA-ANGUKA SANA
(KOTE KOTE) KUKITOKEA
UPEPO

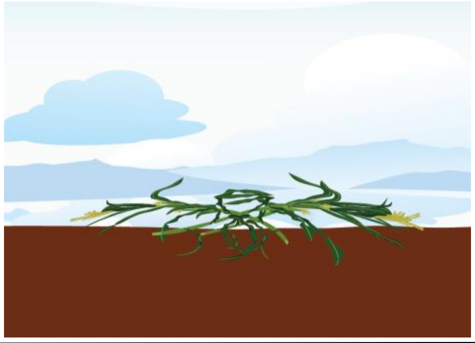

INAVUMULIA KHAYONGO,
HAIDHOOFIKI NA INZALISHA
HATA IKISHMBULIWA NA
KHAYONGO

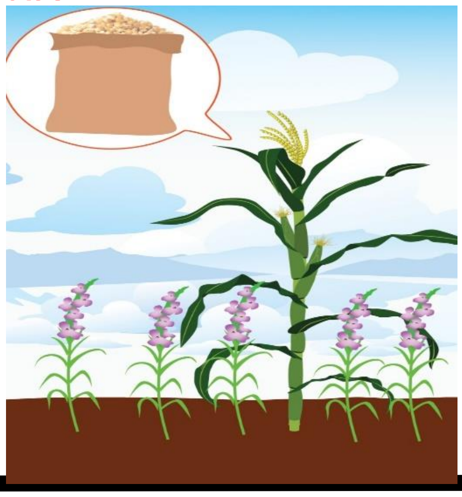

AINA YA MAHINDI –Ya Pili

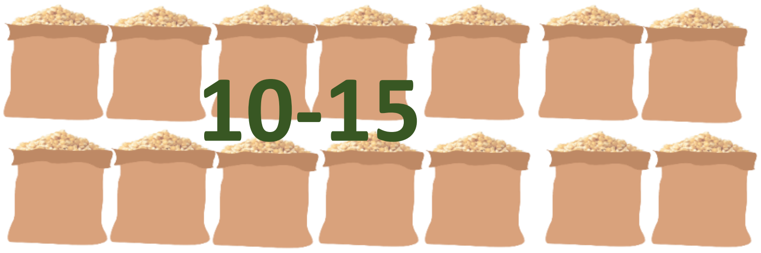

MAZAO GUNIA 10 HADI 15
KWA EKARI MOJA

HAIWEZI KUVUMILIA UKAME,
HAIZALISHI KUKITOKEA UKAME
KATI-KATI YA MSIMU

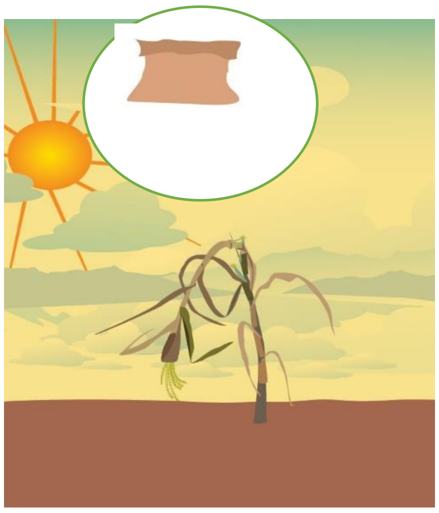

LINABAKI IKISIMAMA
HATA KUKITOKEA UPEPO
MKALI

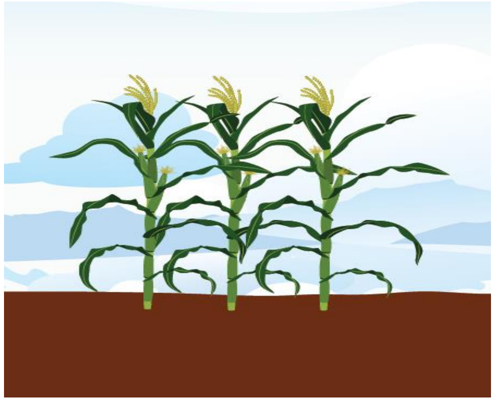

INASHINDWA NA KHAYONGO,
INADHOOFIKA NA HAIZALISHI
IKISHAMBULIWA NA
KHAYONGO

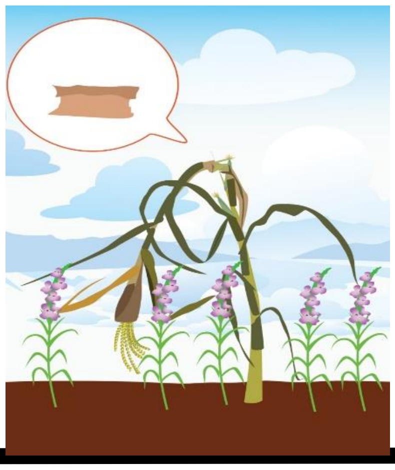

CE-C-Session 2

AINA YA MAHINDI –Ya Kwanza

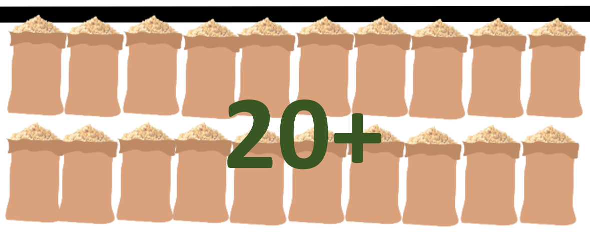

INATOA MAZAO
GUNIA 20 NA ZAIDI
KWA EKARI MOJA

AINA YA MAHINDI –Ya Pili

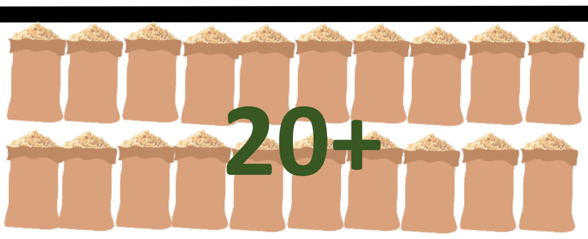

INATOA MAZAO
GUNIA 20 NA ZAIDI
KWA EKARI MOJA

HAIWEZI KUVUMILIA UKAME,
HAIZALISHI KUKITOKEA UKAME
KATI-KATI YA MSIMU

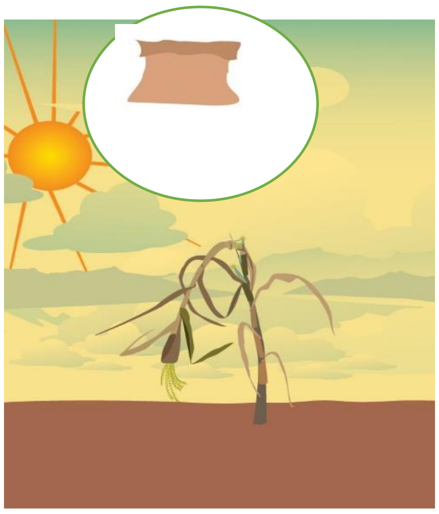

INA-VUMILIA UKAME, INAZLISHA
KIASI HATA KUKITOKEA UKAME
KATI-KATI YA MSIMU

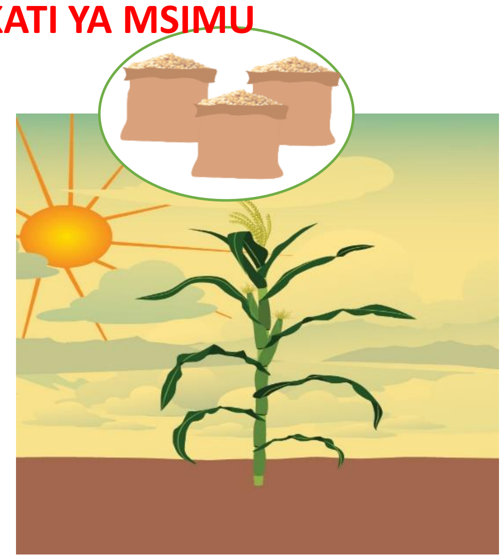

LINABAKI IKISIMAMA
HATA KUKITOKEA UPEPO
MKALI

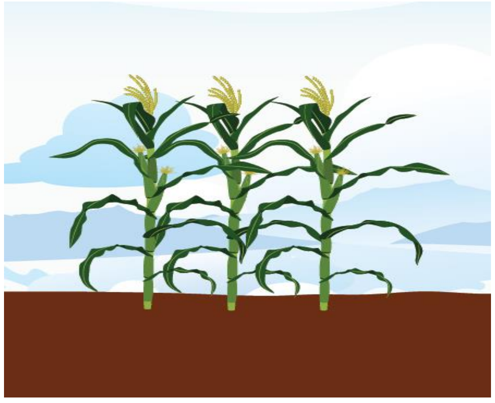

LINA-ANGUKA SANA
(KOTE KOTE) KUKITOKEA
UPEPO

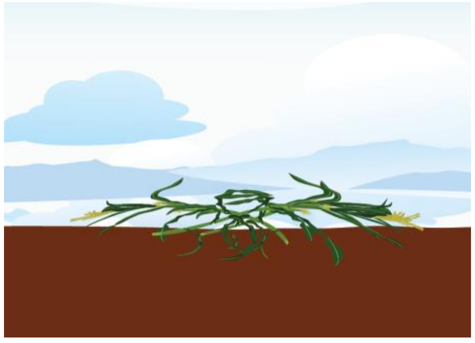

INASHINDWA NA KHAYONGO,
INADHOOFIKA NA HAIZALISHI
IKISHAMBULIWA NA
KHAYONGO

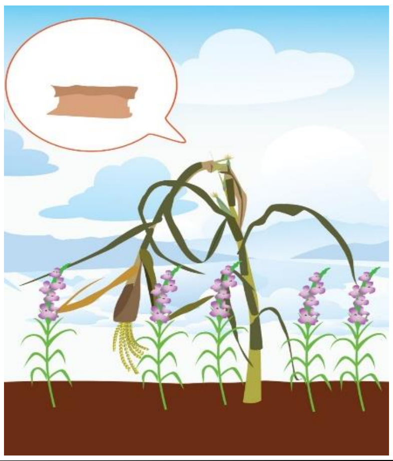

INAVUMULIA KHAYONGO,
HAIDHOOFIKI NA INZALISHA
HATA IKISHMBULIWA NA
KHAYONGO

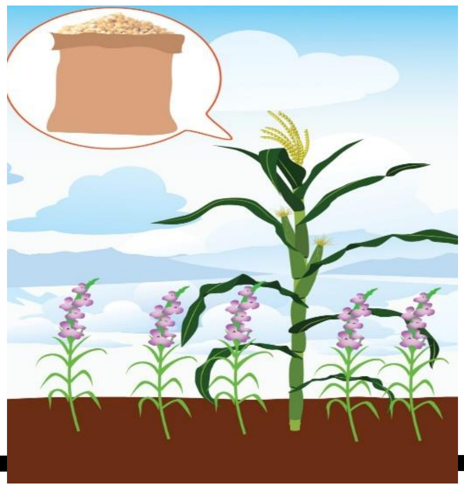

CE-C-Session 3

AINA YA MAHINDI –Ya Kwanza

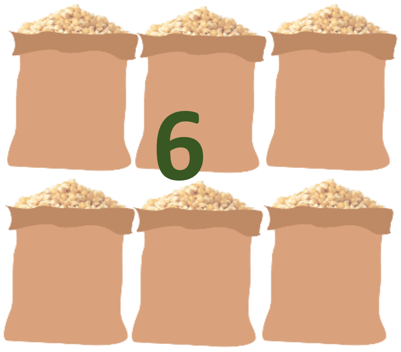

MAZAO GUNIA 6 AU CHINI KWA EKARI MOJA

INA-VUMILIA UKAME, INAZLISHA KIASI HATA KUKITOKEA UKAME KATI-KATI YA MSIMU

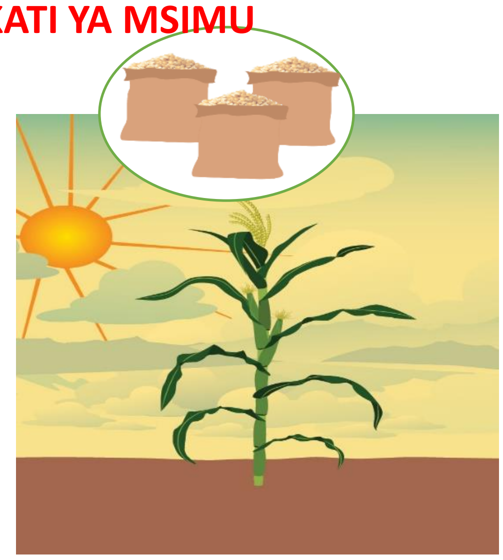

LINA-ANGUKA SANA (KOTE KOTE) KUKITOKEA UPEPO

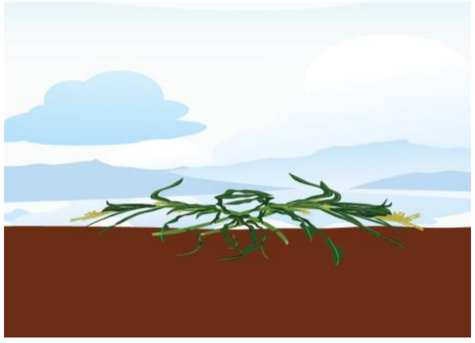

INAVUMULIA KHAYONGO, HAIDHOOFIKI NA INZALISHA HATA IKISHMBULIWA NA KHAYONGO

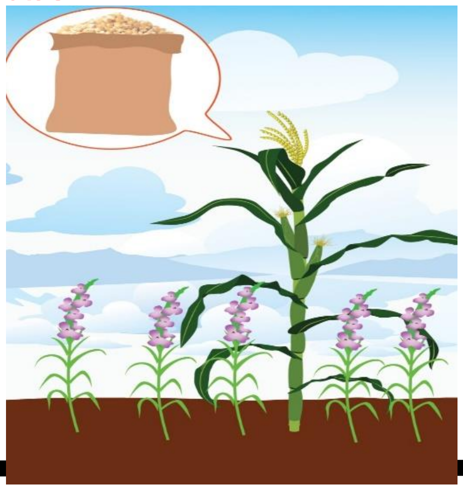

AINA YA MAHINDI –Ya Pili

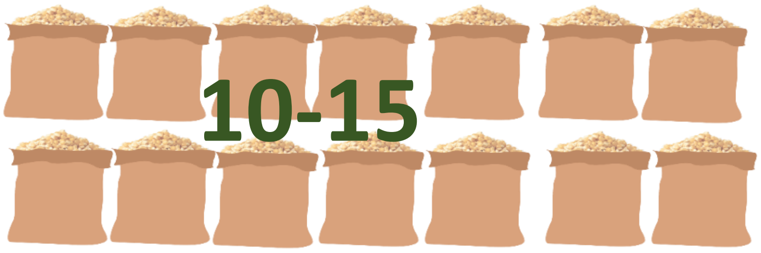

MAZAO GUNIA 10 HADI 15 KWA EKARI MOJA

HAIWEZI KUVUMILIA UKAME, HAIZALISHI KUKITOKEA UKAME KATI-KATI YA MSIMU

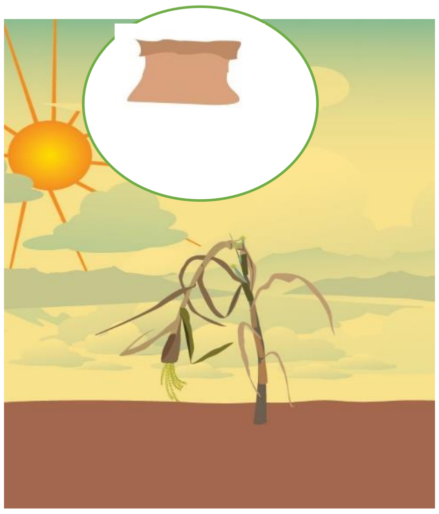

LINABAKI IKISIMAMA HATA KUKITOKEA UPEPO MKALI

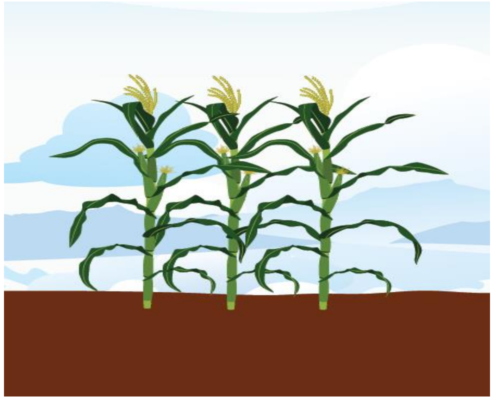

INASHINDWA NA KHAYONGO, INADHOOFIKA NA HAIZALISHI IKISHAMBULIWA NA KHAYONGO

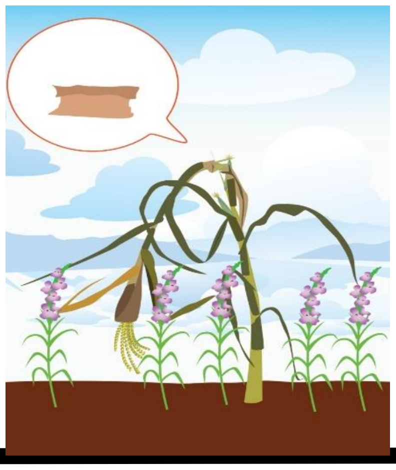

CE-C-Session 4

AINA YA MAHINDI –Ya Kwanza	AINA YA MAHINDI –Ya Pili
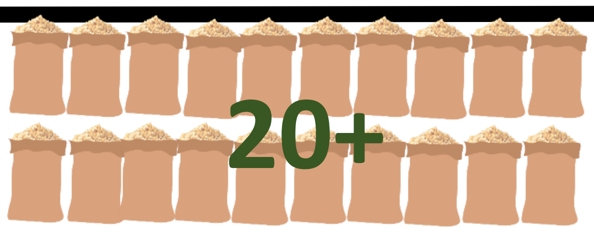 INATOA MAZAO GUNIA 20 NA ZAIDI KWA EKARI MOJA	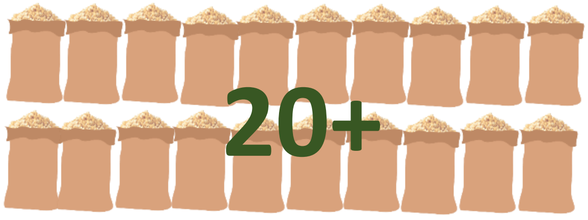 INATOA MAZAO GUNIA 20 NA ZAIDI KWA EKARI MOJA
INA-VUMILIA UKAME, INAZLISHA KIASI HATA KUKITOKEA UKAME KATI-KATI YA MSIMU 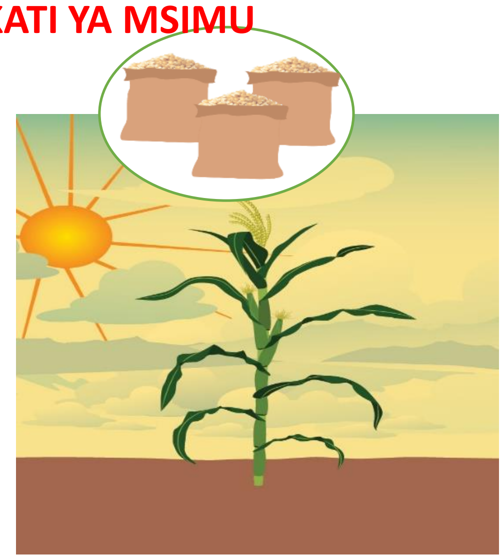	HAIWEZI KUVUMILIA UKAME, HAIZALISHI KUKITOKEA UKAME KATI-KATI YA MSIMU 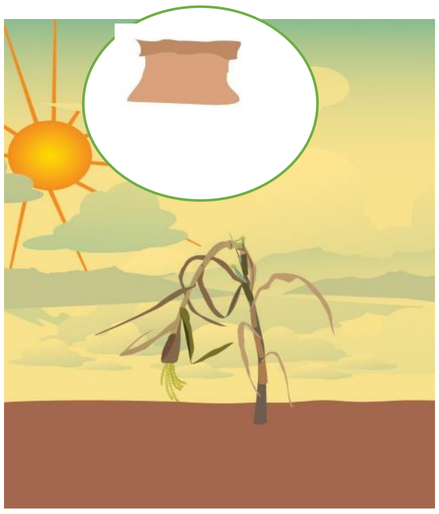
LINA-ANGUKA KADIRI KUKITOKEA MVUA YA UPEPO 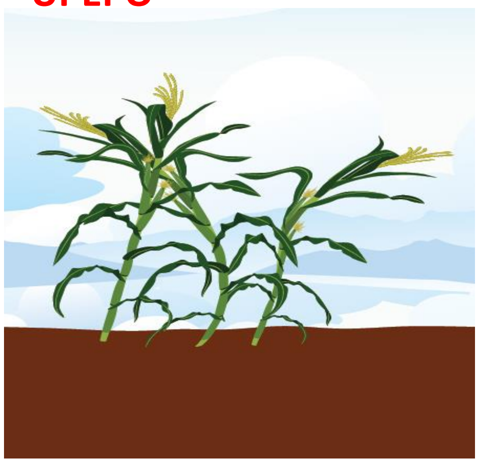	LINA-ANGUKA KADIRI KUKITOKEA MVUA YA UPEPO 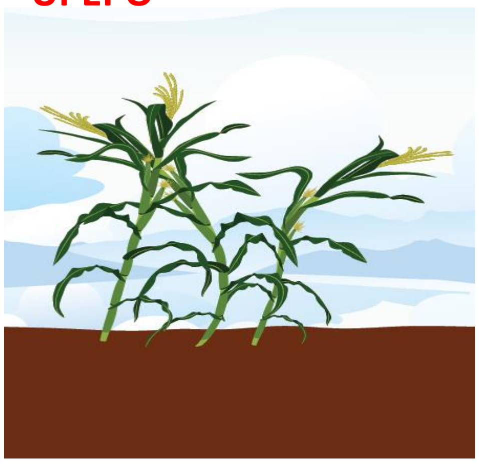
INASHINDWA NA KHAYONGO, INADHOOFIKA NA HAIZALISHI IKISHAMBULIWA NA KHAYONGO 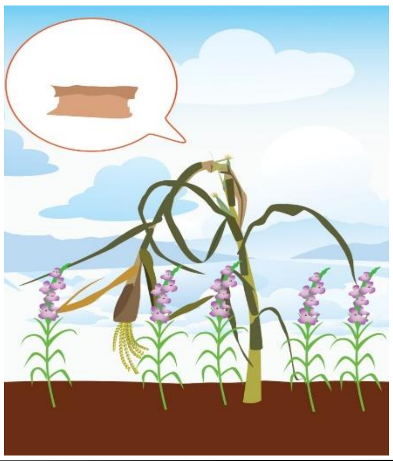	INAVUMULIA KHAYONGO, HAIDHOOFIKI NA INZALISHA HATA IKISHMBULIWA NA KHAYONGO 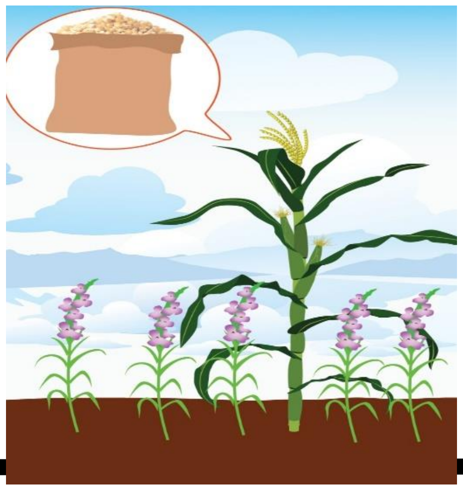

CE-C-Session 5

AINA YA MAHINDI –Ya Kwanza

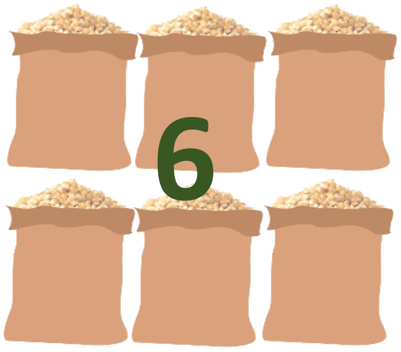

MAZAO GUNIA 6 AU CHINI KWA EKARI MOJA

INA-VUMILIA UKAME, INAZLISHA KIASI HATA KUKITOKEA UKAME KATI-KATI YA MSIMU

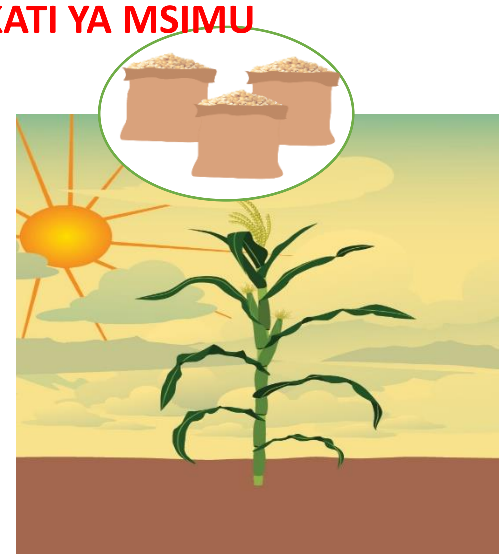

LINASIMAMA HATA KUKITOKEA UPEPO MKALI

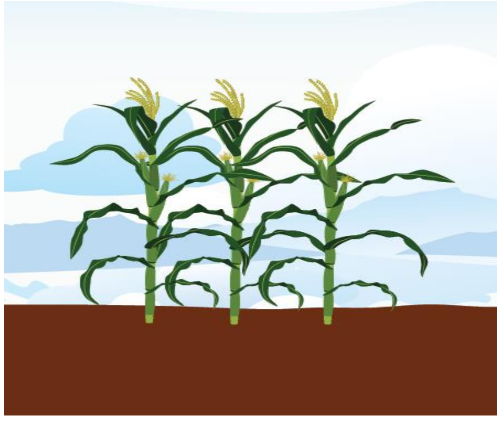

INASHINDWA NA KHAYONGO, INADHOOFIKA NA HAIZALISHI IKISHAMBULIWA NA KHAYONGO

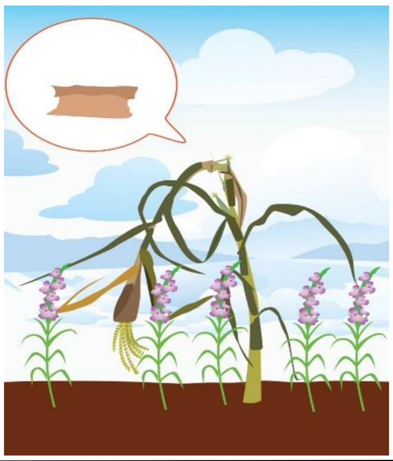

AINA YA MAHINDI –Ya Pili

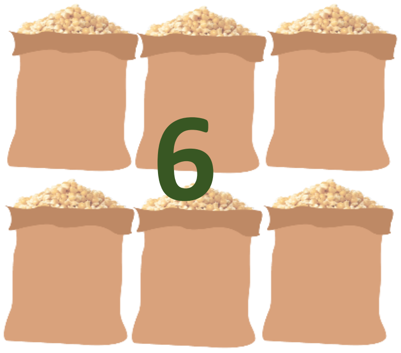

MAZAO GUNIA 6 AU CHINI KWA EKARI MOJA

HAIWEZI KUVUMILIA UKAME, HAIZALISHI KUKITOKEA UKAME KATI-KATI YA MSIMU

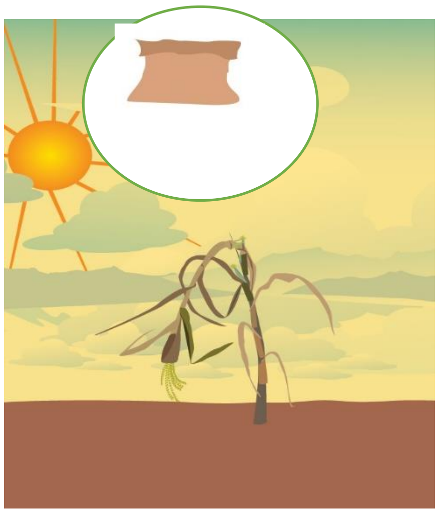

LINA-ANGUKA SANA (KOTE KOTE) KUKITOKEA UPEPO

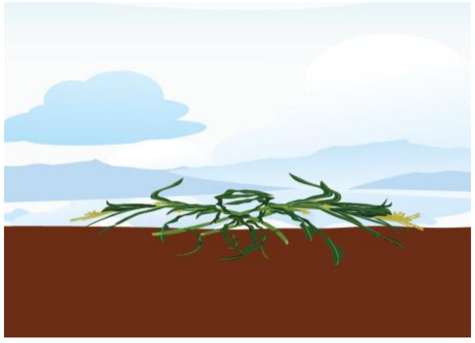

INAVUMULIA KHAYONGO, HAIDHOOFIKI NA INZALISHA HATA IKISHMBULIWA NA KHAYONGO

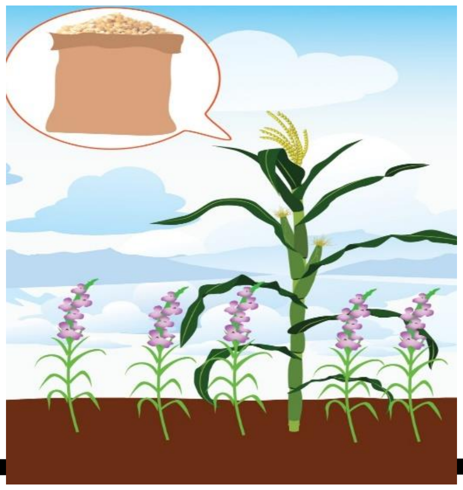

CE-C-Session 6

AINA YA MAHINDI –Ya Kwanza	AINA YA MAHINDI –Ya Pili
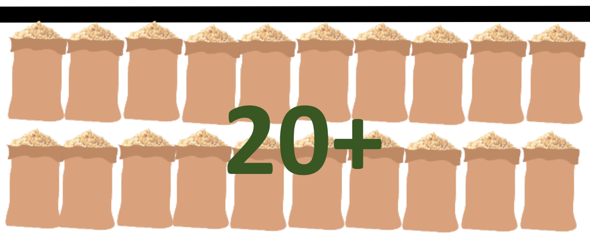 INATOA MAZAO GUNIA 20 NA ZAIDI KWA EKARI MOJA	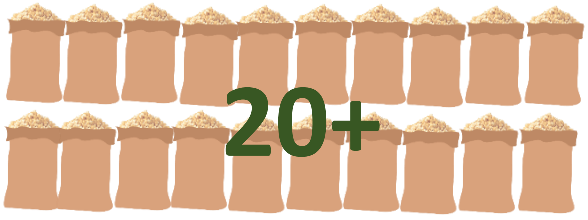 INATOA MAZAO GUNIA 20 NA ZAIDI KWA EKARI MOJA
HAIWEZI KUVUMILIA UKAME, HAIZALISHI KUKITOKEA UKAME KATI-KATI YA MSIMU 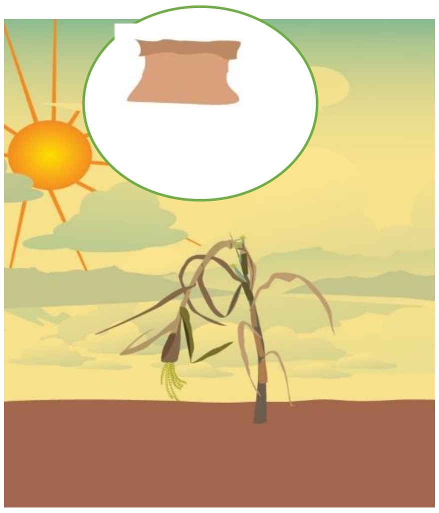	INA-VUMILIA UKAME, INAZLISHA KIASI HATA KUKITOKEA UKAME KATI-KATI YA MSIMU 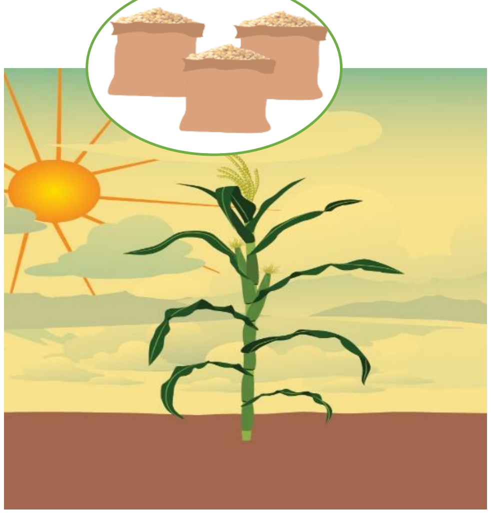
LINA-ANGUKA SANA (KOTE KOTE) KUKITOKEA UPEPO 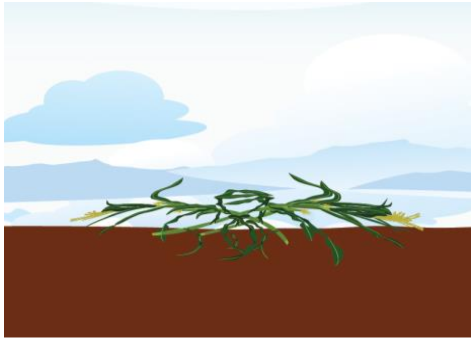	LINASIMAMA HATA KUKITOKEA UPEPO MKALI 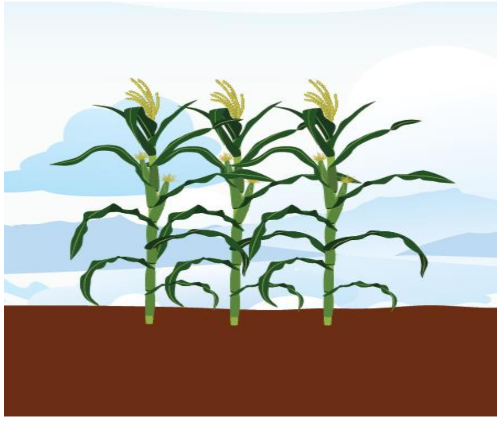
INAVUMULIA KHAYONGO, HAIDHOOFIKI NA INZALISHA HATA IKISHMBULIWA NA KHAYONGO 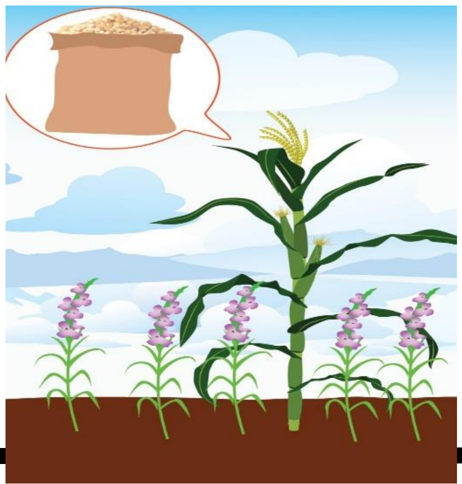	INASHINDWA NA KHAYONGO, INADHOOFIKA NA HAIZALISHI IKISHAMBULIWA NA KHAYONGO 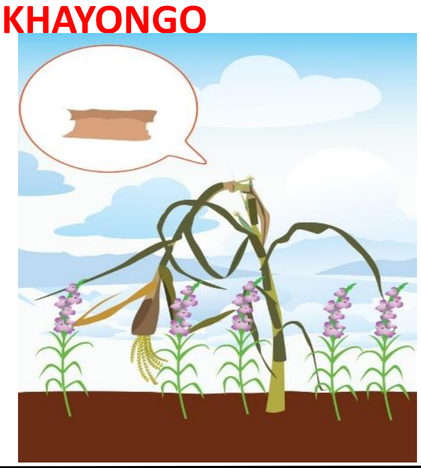

CE-C-Session 7

AINA YA MAHINDI –Ya Kwanza

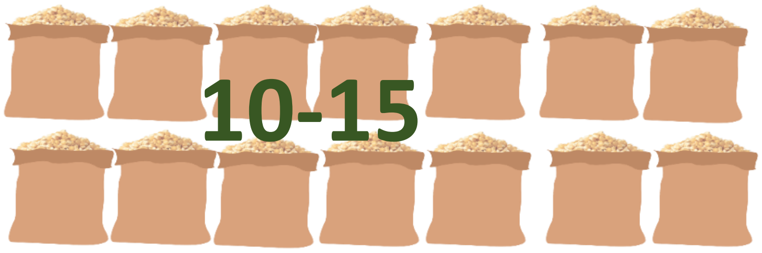

MAZAO GUNIA 10 HADI 15
KWA EKARI MOJA

HAIWEZI KUVUMILIA UKAME,
HAIZALISHI KUKITOKEA UKAME
KATI-KATI YA MSIMU

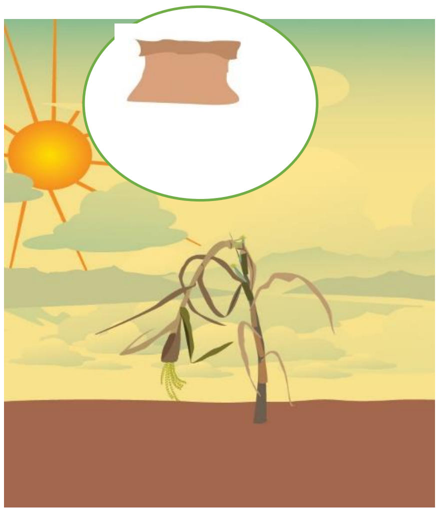

LINA-ANGUKA
KADIRI KUKITOKEA
MVUA YA UPEPO

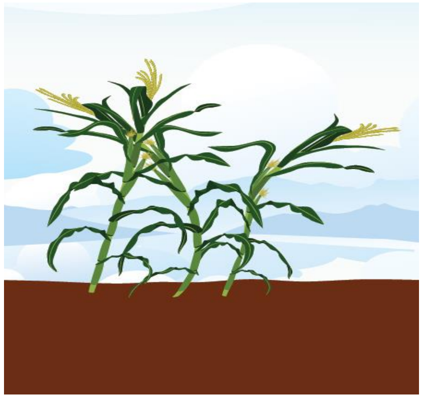

INAVUMULIA KHAYONGO,
HAIDHOOFIKI NA INZALISHA
HATA IKISHMBULIWA NA
KHAYONGO

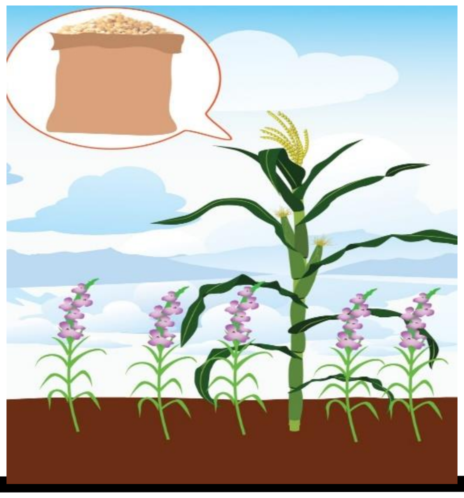

AINA YA MAHINDI –Ya Pili

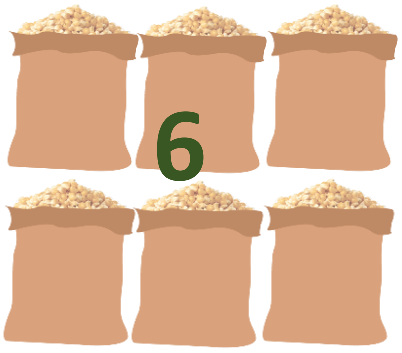

MAZAO GUNIA 6 AU CHINI KWA
EKARI MOJA

INA-VUMILIA UKAME, INAZLISHA
KIASI HATA KUKITOKEA UKAME
KATI-KATI YA MSIMU

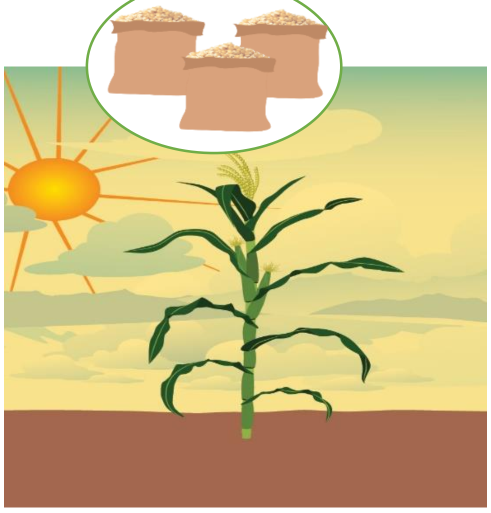

LINA-ANGUKA
KADIRI KUKITOKEA
MVUA YA UPEPO

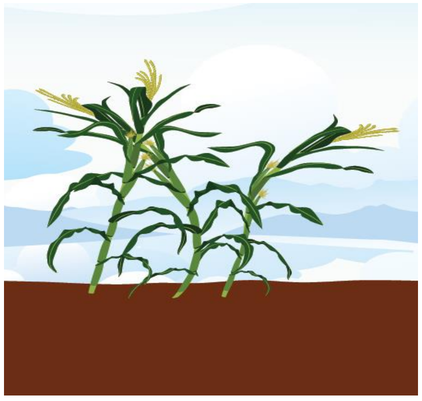

INAVUMULIA KHAYONGO,
HAIDHOOFIKI NA INZALISHA
HATA IKISHMBULIWA NA
KHAYONGO

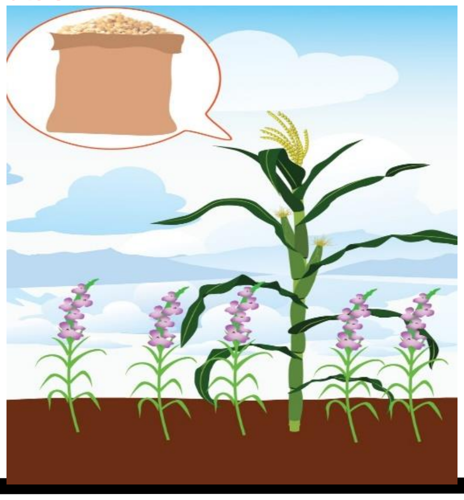

CE-C-Session 8

AINA YA MAHINDI –Ya Kwanza	AINA YA MAHINDI –Ya Pili
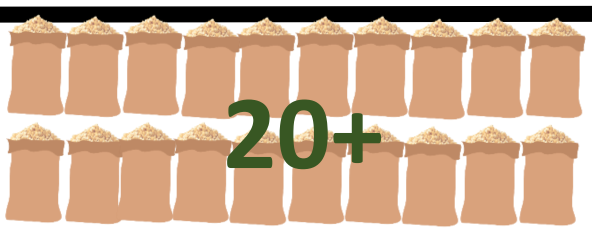	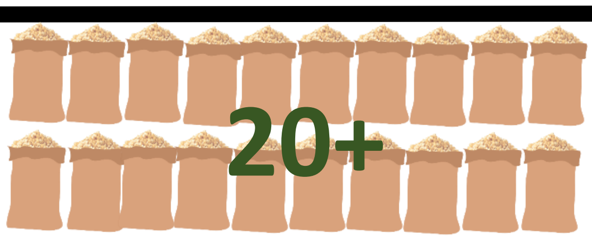
INATOA MAZAO GUNIA 20 NA ZAIDI KWA EKARI MOJA	INATOA MAZAO GUNIA 20 NA ZAIDI KWA EKARI MOJA
HAIWEZI KUVUMILIA UKAME, HAIZALISHI KUKITOKEA UKAME KATI-KATI YA MSIMU	INA-VUMILIA UKAME, INAZLISHA KIASI HATA KUKITOKEA UKAME KATI-KATI YA MSIMU
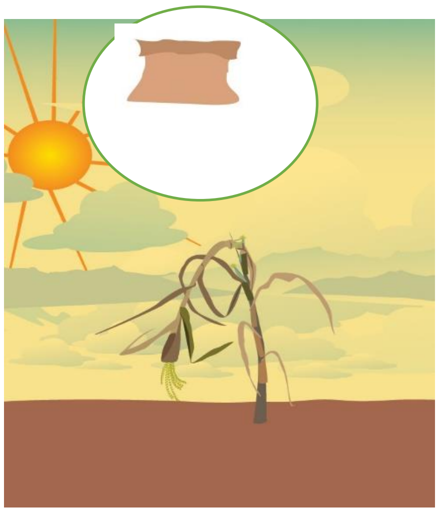	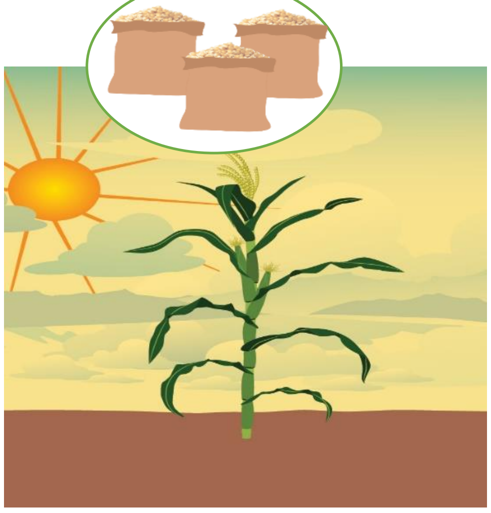
LINASIMAMA HATA KUKITOKEA UPEPO MKALI	LINA-ANGUKA SANA (KOTE KOTE) KUKITOKEA UPEPO
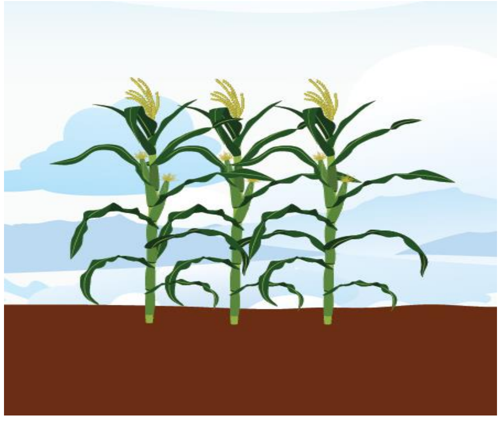	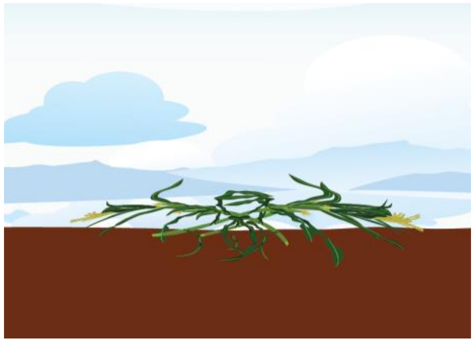
INAVUMULIA KHAYONGO, HAIDHOOFIKI NA INZALISHA HATA IKISHMBULIWA NA KHAYONGO	INASHINDWA NA KHAYONGO, INADHOOFIKA NA HAIZALISHI IKISHAMBULIWA NA KHAYONGO
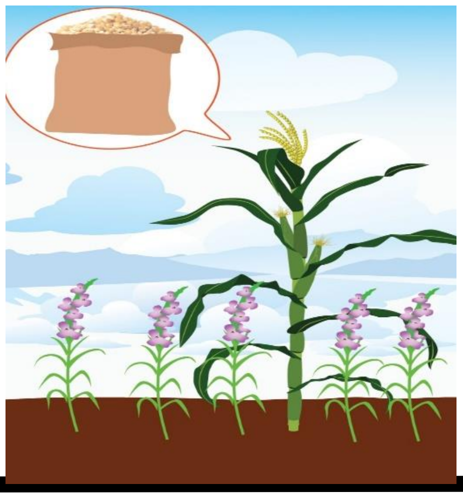	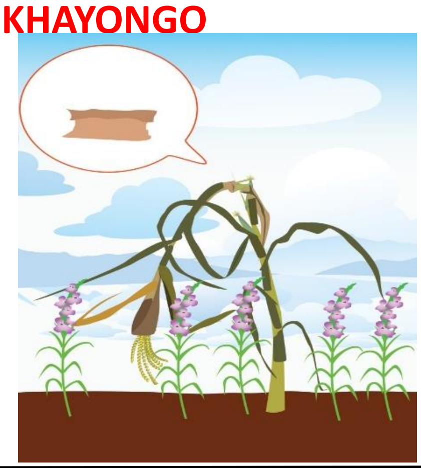

CE-C-Session 9

AINA YA MAHINDI –Ya Kwanza

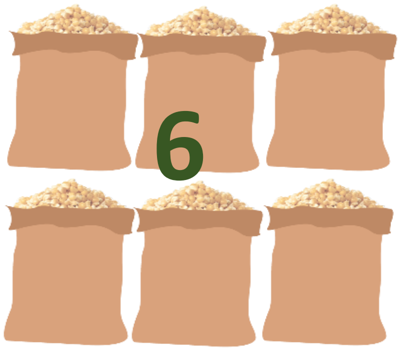

MAZAO GUNIA 6 AU CHINI KWA EKARI MOJA

INA-VUMILIA UKAME, INAZLISHA KIASI HATA KUKITOKEA UKAME KATI-KATI YA MSIMU

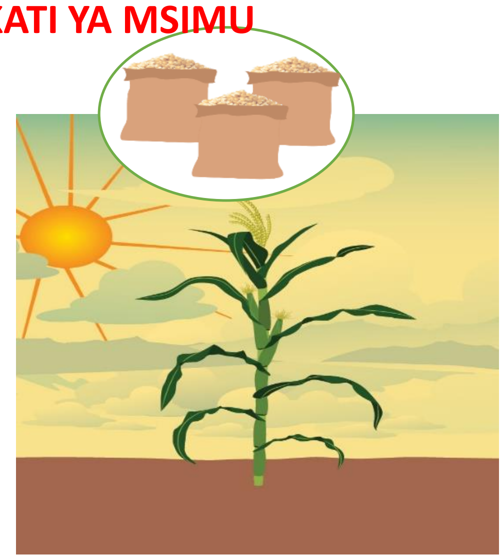

LINASIMAMA HATA KUKITOKEA UPEPO MKALI

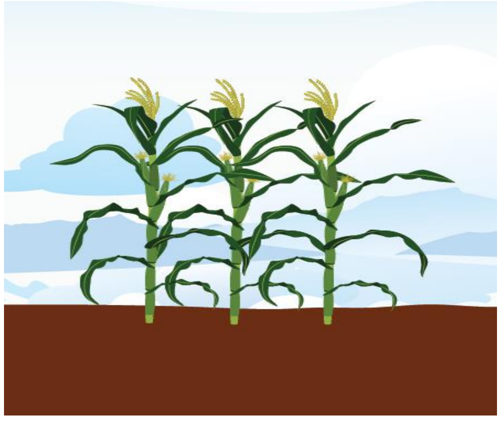

INAVUMULIA KHAYONGO, HAIDHOOFIKI NA INZALISHA HATA IKISHMBULIWA NA KHAYONGO

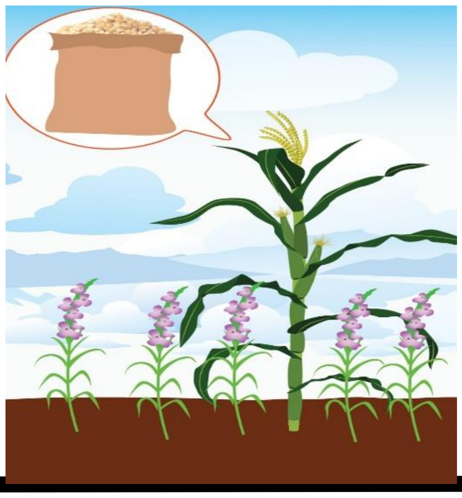

AINA YA MAHINDI –Ya Pili

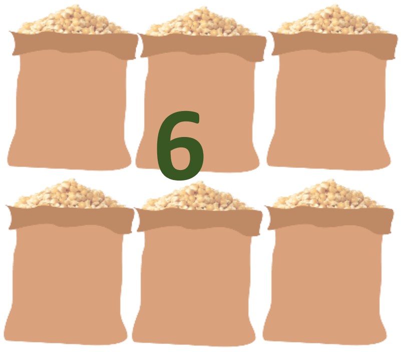

MAZAO GUNIA 6 AU CHINI KWA EKARI MOJA

HAIWEZI KUVUMILIA UKAME, HAIZALISHI KUKITOKEA UKAME KATI-KATI YA MSIMU

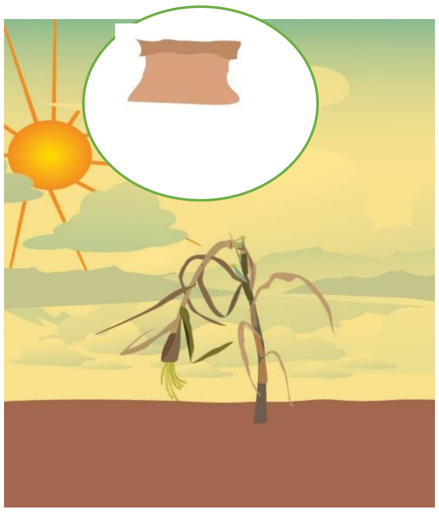

LINA-ANGUKA SANA (KOTE KOTE) KUKITOKEA UPEPO

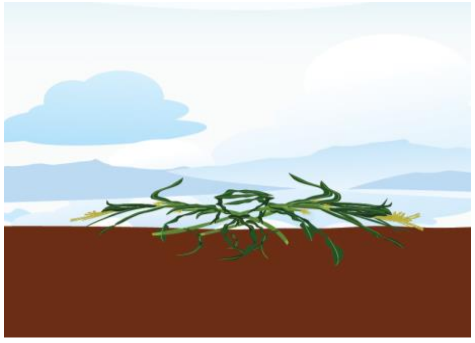

INASHINDWA NA KHAYONGO, INADHOOFIKA NA HAIZALISHI IKISHAMBULIWA NA KHAYONGO

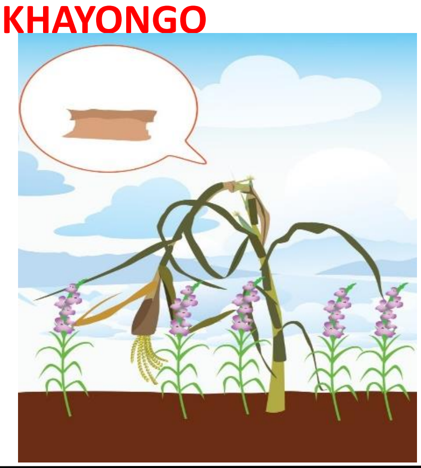

CE-C-Session 10

AINA YA MAHINDI –Ya Kwanza

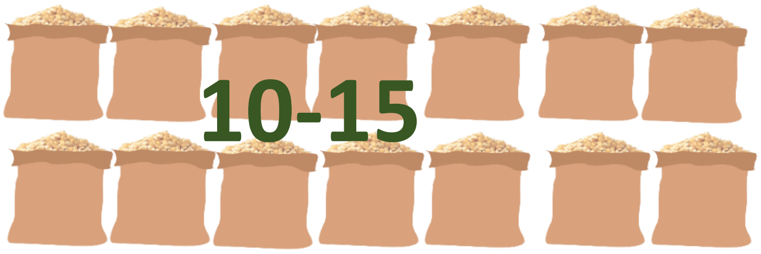

MAZAO GUNIA 10 HADI 15
KWA EKARI MOJA

HAIWEZI KUVUMILIA UKAME,
HAIZALISHI KUKITOKEA UKAME
KATI-KATI YA MSIMU

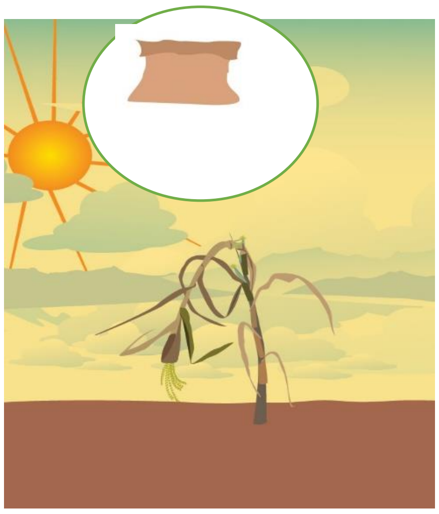

LINA-ANGUKA
KADIRI KUKITOKEA
MVUA YA UPEPO

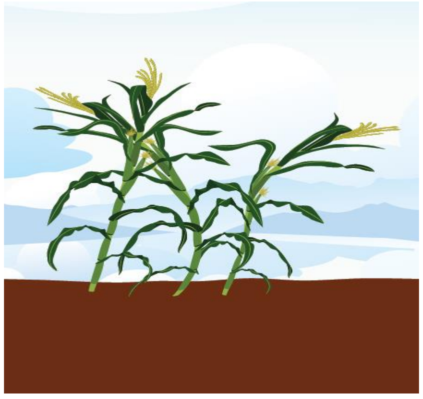

INASHINDWA NA KHAYONGO,
INADHOOFIKA NA HAIZALISHI
IKISHAMBULIWA NA KHAYONGO

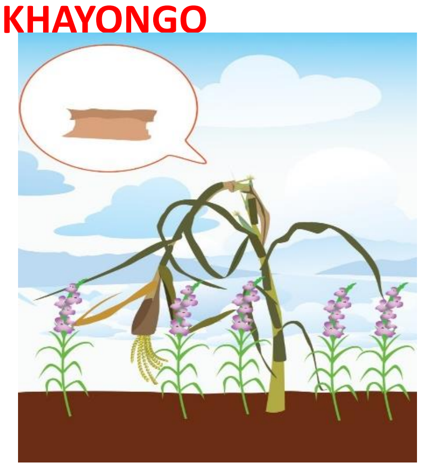

AINA YA MAHINDI –Ya Pili

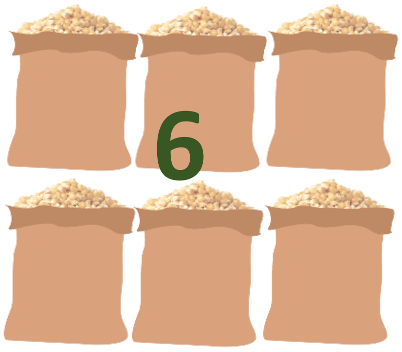

MAZAO GUNIA 6 AU CHINI KWA
EKARI MOJA

INA-VUMILIA UKAME, INAZLISHA
KIASI HATA KUKITOKEA UKAME
KATI-KATI YA MSIMU

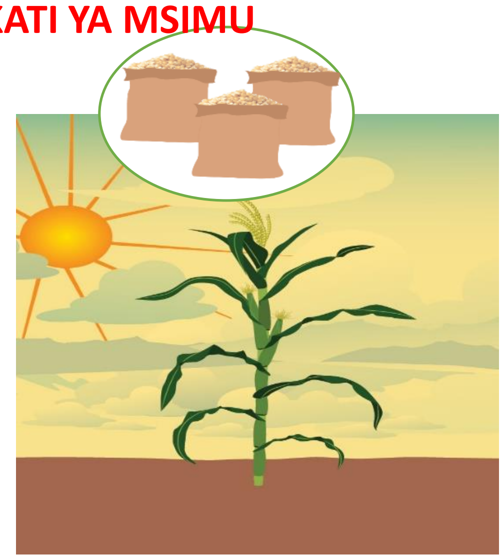

LINA-ANGUKA
KADIRI KUKITOKEA
MVUA YA UPEPO

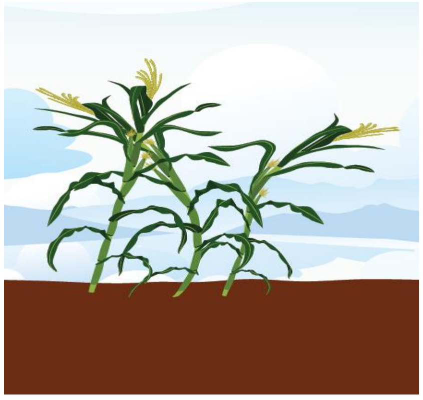

INAVUMULIA KHAYONGO,
HAIDHOOFIKI NA INZALISHA
HATA IKISHMBULIWA NA
KHAYONGO

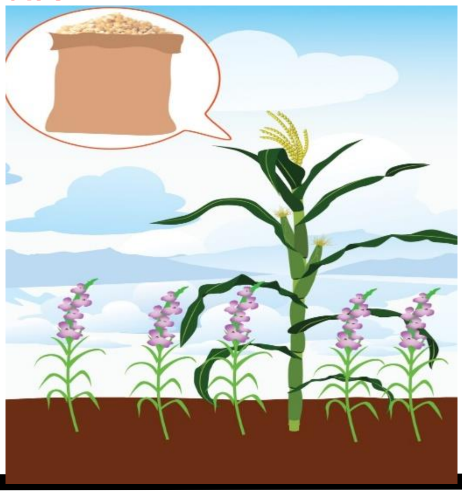

CE-C-Session 11

AINA YA MAHINDI –Ya Kwanza

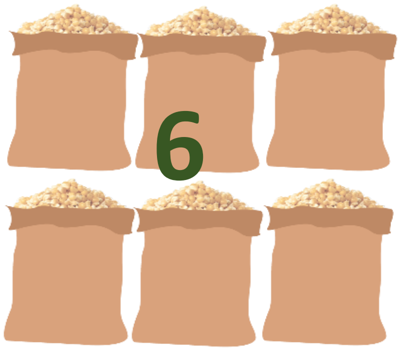

MAZAO GUNIA 6 AU CHINI KWA EKARI MOJA

INA-VUMILIA UKAME, INAZLISHA KIASI HATA KUKITOKEA UKAME KATI-KATI YA MSIMU

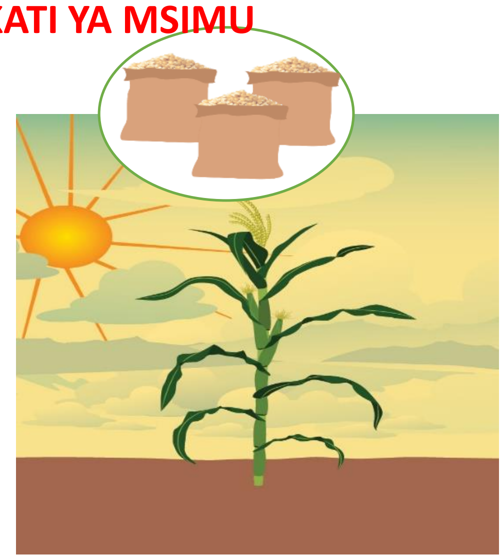

LINA-ANGUKA KADIRI KUKITOKEA MVUA YA UPEPO

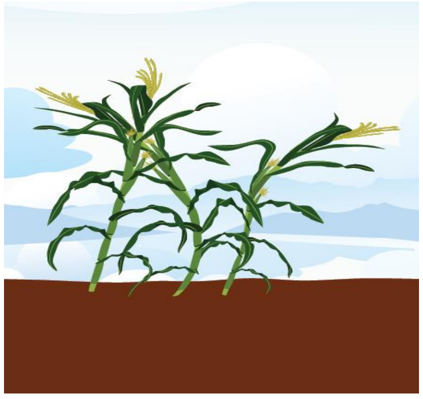

INASHINDWA NA KHAYONGO, INADHOOFIKA NA HAIZALISHI IKISHAMBULIWA NA KHAYONGO

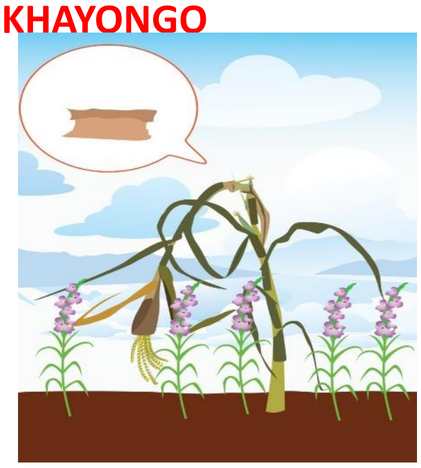

AINA YA MAHINDI –Ya Pili

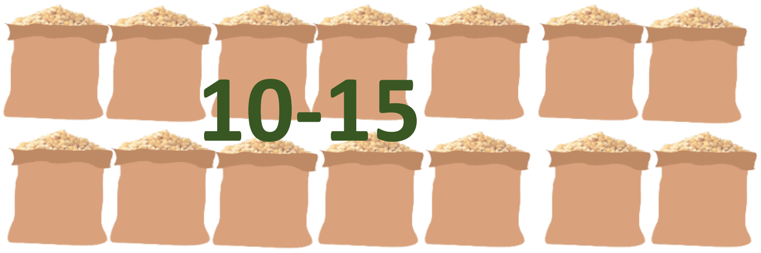

MAZAO GUNIA 10 HADI 15 KWA EKARI MOJA

HAIWEZI KUVUMILIA UKAME, HAIZALISHI KUKITOKEA UKAME KATI-KATI YA MSIMU

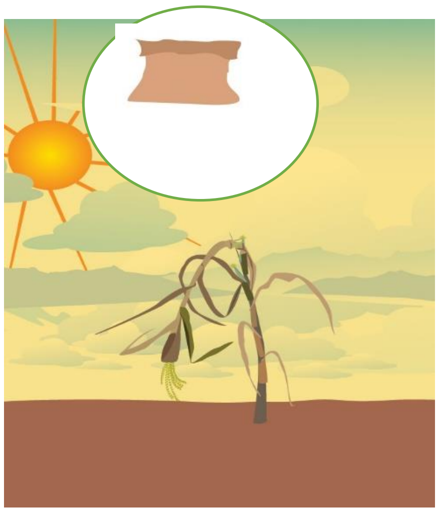

LINA-ANGUKA KADIRI KUKITOKEA MVUA YA UPEPO

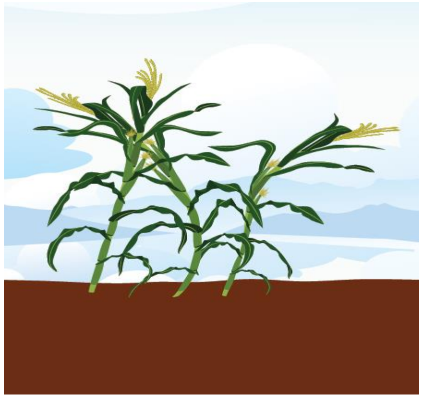

INASHINDWA NA KHAYONGO, INADHOOFIKA NA HAIZALISHI IKISHAMBULIWA NA KHAYONGO

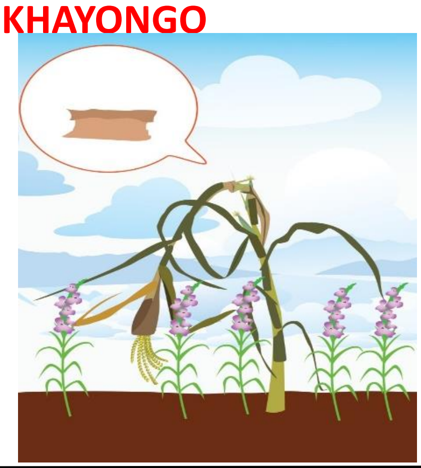

CE-C-Session 12

AINA YA MAHINDI –Ya Kwanza

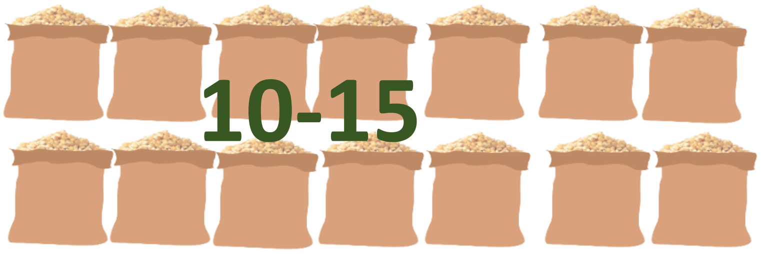

MAZAO GUNIA 10 HADI 15
KWA EKARI MOJA

HAIWEZI KUVUMILIA UKAME,
HAIZALISHI KUKITOKEA UKAME
KATI-KATI YA MSIMU

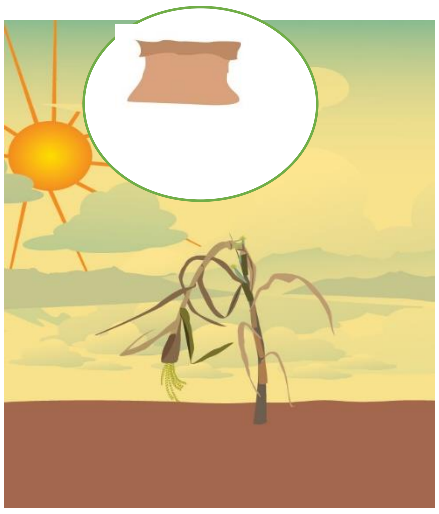

LINA-ANGUKA SANA
(KOTE KOTE) KUKITOKEA
UPEPO

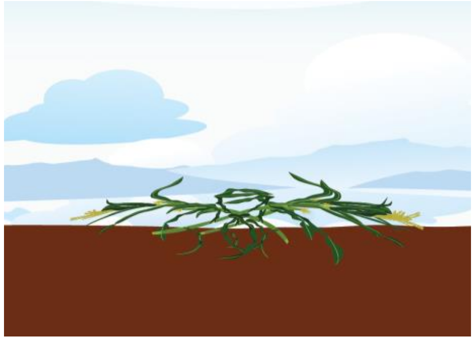

INASHINDWA NA KHAYONGO,
INADHOOFIKA NA HAIZALISHI
IKISHAMBULIWA NA KHAYONGO

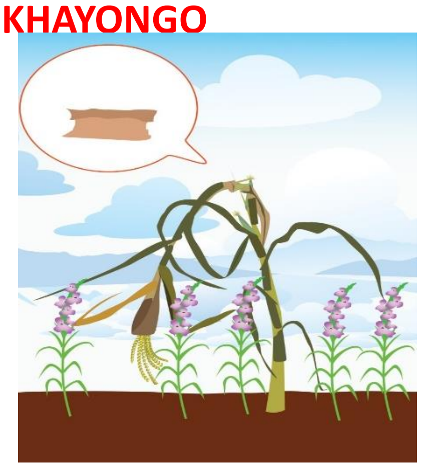

AINA YA MAHINDI –Ya Pili

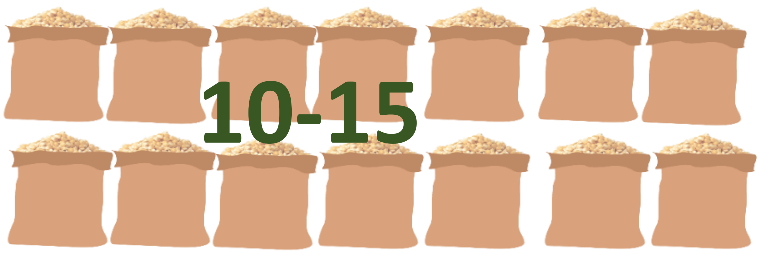

MAZAO GUNIA 10 HADI 15
KWA EKARI MOJA

INA-VUMILIA UKAME, INAZLISHA
KIASI HATA KUKITOKEA UKAME
KATI-KATI YA MSIMU

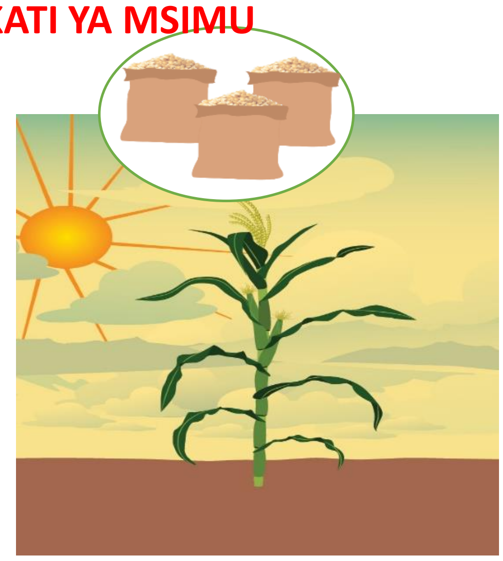

LINASIMAMA HATA
KUKITOKEA UPEPO MKALI

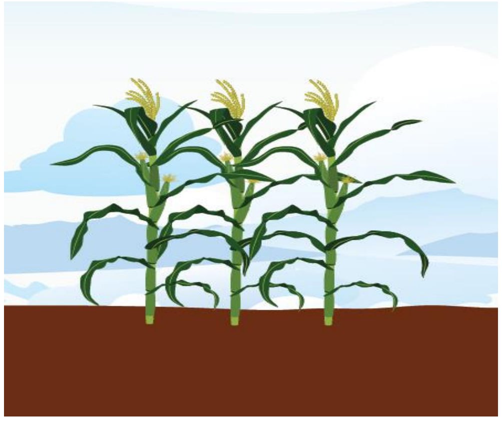

INAVUMULIA KHAYONGO,
HAIDHOOFIKI NA INZALISHA
HATA IKISHMBULIWA NA
KHAYONGO

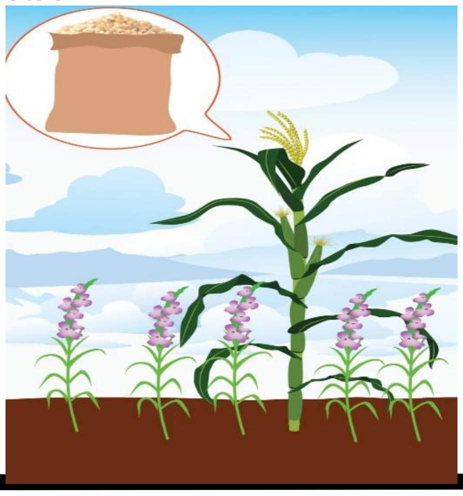

Supplement: Kenya-Choce-Experiment-C.pdf [file mmc3.pdf]
