# Supplementary material for: Trait preference trade-offs among maize farmers in western Kenya
Source: Heliyon. 2021 Mar 12;7(3):e06389. doi: 10.1016/j.heliyon.2021.e06389 (PMC7970324; doi:10.1016/j.heliyon.2021.e06389)
Supplement: Focus Group Disucssion Questions.docx [file mmc4.docx]

**Focus Group Discussion (FGD)**

1. **Objectives of the FGD**

- To outline the major attributes that affect maize variety selection by the farmers.
- To determine tradeoffs in variety attributes in variety choice
- To help seed companies target, differentiate and position their varieties

Introduction:

Good afternoon and welcome to our focus group session. Thank you for taking the time to come here.

My name is XXX XXX.

I work a team of researchers from CIMMYT and KALRO (Kakamega). I would like to get your input and I want you to share your ideas or suggestions regarding the major attributes that affects maize seed variety selection. Many people don’t realize this but maize is one of the most important staple crops and occupies the largest area of all crops in Ethiopia.

During this discussion I would like to emphasize, there are no right or wrong answers but rather various points of view. Please feel free to share your point of view even if it differs from what others have said. We will try to give each person a chance to talk so be patient as your colleague talks. Please keep in mind also that we’re interested in all types of comments such as those which some people may call negative comments or positive comments. Again there are no right or wrong comments and no comment is better than another. ,

Our session will last about an hour, we can take a bit more time if we need to so no one is rushed. But before we ask the first question, let’s find out some more about each other. Tell us your name and how long have you been working/Farming.

1. **Major topics for discussion**

**Introduction**

We are going to try to understand farmers’ choices as you understand from the point of view of seed company officials. You have interacted with farmers and sell seed to them. So you understand their seed demand patterns better than most people do. For example:

1. What are the things farmers that influence farmers’ choices of seed?
2. Which traits do you think a variety must have to be successful?
3. Which traits do you think farmers must and have and are willing to sacrifice other traits in order to have this trait?
4. What type of maize varieties are you basically producing?
   1. Is there anything in a variety that made you to stop using it? Why?
5. What type of maize varieties are you basically producing?
6. Which maize disease is most common in your area
7. What are more important for you
   1. A variety is resistant to this disease you mentions above or that a variety is resistant to drought
   2. That a variety is lodging resistant or resistant to drought
   3. That a variety is early maturing or resistant to drought
   4. That a variety is resistant to Striga or resistant to drought
8. When you go to buy maize, how do you judge that it will be good for Ugali (posho),
9. What is the most common use of Maize in your area?
10. What would a good Ugali taste like?
11. Some varieties have the quality of mid-season drought tolerance. This means that when rains fail (disappear) just before or during the flowering they stay green and eventually yield well OK? (The yield doesn’t go down too much).
    1. Then how do you measure this trait of drought tolerance?
    2. Do you have such varieties?

**NOTES**

1. **What are the things farmers that influence farmers’ choices of seed?**
2. **Which traits do you think a variety must have to be successful?**

| **No** | **Traits list** | **Description** |
| --- | --- | --- |
| **1** |  |  |
| **2** |  |  |
| **3** |  |  |
| **4** |  |  |
| **5** |  |  |
| **6** |  |  |
| **7** |  |  |
| **8** |  |  |
| **9** |  |  |
| **10** |  |  |
| **11** |  |  |
| **12** |  |  |
| **13** |  |  |
| **14** |  |  |

1. **Which traits do you think farmers must and have and are willing to sacrifice other traits in order to have this trait?**

| **No** | **Traits list** | **Description** |
| --- | --- | --- |
| **1** |  |  |
| **2** |  |  |
| **3** |  |  |
| **4** |  |  |
| **5** |  |  |
| **6** |  |  |
| **7** |  |  |
| **8** |  |  |
| **9** |  |  |
| **10** |  |  |
| **11** |  |  |
| **12** |  |  |
| **13** |  |  |
| **14** |  |  |
| **15** |  |  |
| **16** |  |  |

1. **What type of maize varieties are you basically producing?**

| **No** | **Type of maize varieties** | **Description** |
| --- | --- | --- |
| **1** |  |  |
| **2** |  |  |
| **3** |  |  |
| **4** |  |  |
| **5** |  |  |
| **6** |  |  |
| **7** |  |  |
| **8** |  |  |
| **9** |  |  |
| **10** |  |  |
| **11** |  |  |
| **12** |  |  |
| **13** |  |  |
| **14** |  |  |
| **15** |  |  |
| **16** |  |  |

1. **Is there anything in a variety that made you to stop using it? Why?**

| **No** | **Maize variety list** | **Description** |
| --- | --- | --- |
| **1** |  |  |
| **2** |  |  |
| **3** |  |  |
| **4** |  |  |
| **5** |  |  |
| **6** |  |  |
| **7** |  |  |

1. **Which maize disease is most common in your area?**
2. **Which maize weed is most common in your area?**
3. **Which maize pest is most common in your area?**
4. **What are more important for you**
   1. A variety is resistant to this disease you mentions above or that a variety is resistant to drought
   2. That a variety is lodging resistant or resistant to drought
   3. That a variety is early maturing or resistant to drought
   4. That a variety is resistant to striga or resistant to drough
5. **When you go to buy maize, how do you judge that it will be good for Ugali, Uji….**
6. **What is the most common use for Maize in this area?**
   1. Can you describe what a good tasting Ugali or Uji tates like?
7. **Some varieties have the quality of mid-season drought tolerance. This means that when rain fail (disappear) just before for during the flowering they stay green and eventually yield well OK? (The yield doesn’t go down too much).**
   1. *Then how do you measure this trait of drought tolerance? (LEVELS)*
   2. *Do you have such varieties?*
