# Supplementary material for: Trait preference trade-offs among maize farmers in western Kenya
Source: Heliyon. 2021 Mar 12;7(3):e06389. doi: 10.1016/j.heliyon.2021.e06389 (PMC7970324; doi:10.1016/j.heliyon.2021.e06389)

CE-A-Session 1

AINA YA MAHINDI –Ya Kwanza

AINA YA MAHINDI –Ya Pili

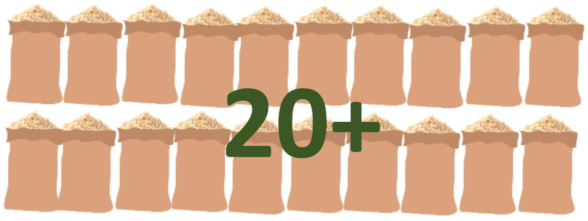

INATOA MAZAO GUNIA 20 NA ZAIDI KWA EKARI MOJA

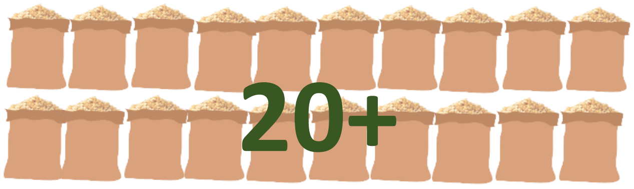

INATOA MAZAO GUNIA 20 NA ZAIDI KWA EKARI MOJA

MBEGU NDOGO-NDOGO

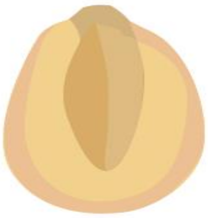

MBEGU MAKUBWA-MAKUBWA

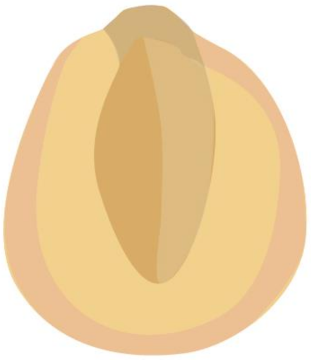

INAKOMAA KWA MIEZI 4 AU ZAIDI

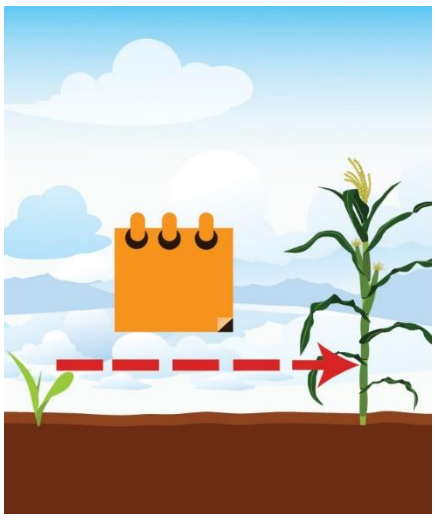

HAIPITI MIEZI 3 KABLA HAIJAKOMAA

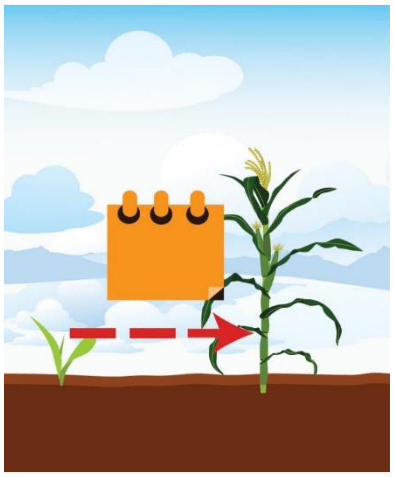

UNAWEZA WEKA BILA KULIWA NA WADUDU HATA UKIWEKA KWA MIEZI 3 HADI 4

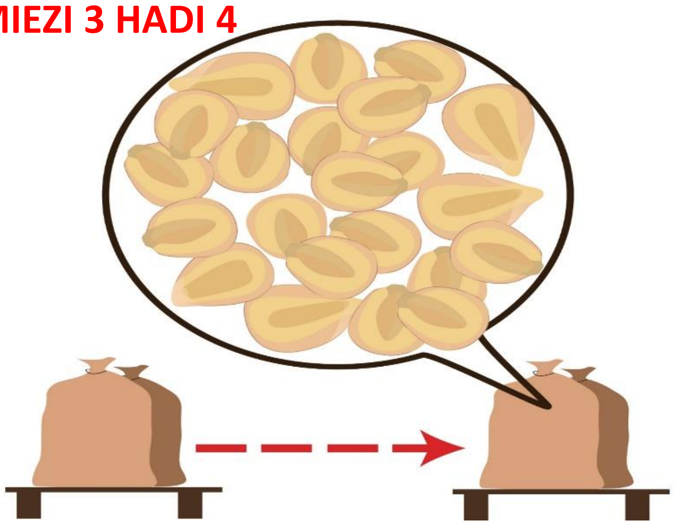

INAKULIWA NA WADUDU IKIKAA MWEZI MOJA AU MBILI

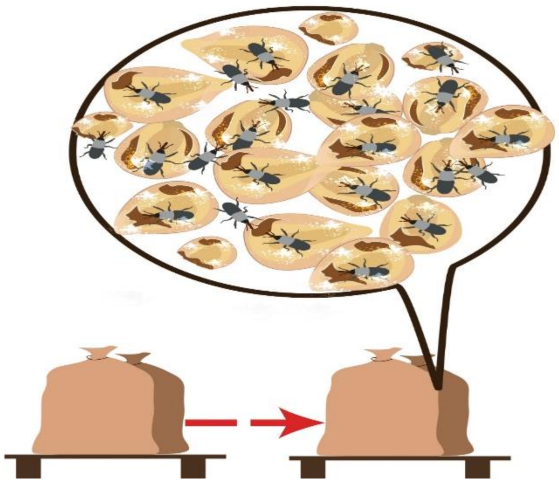

CE-A-Session 2

AINA YA MAHINDI –Ya Kwanza

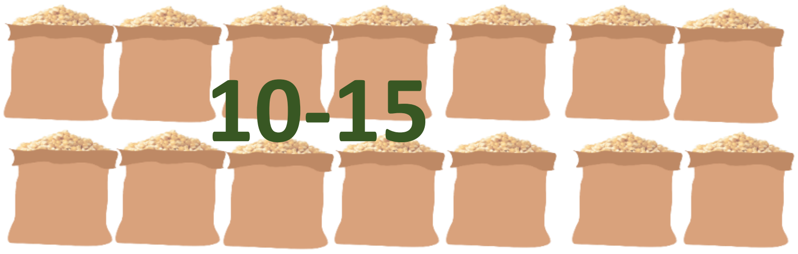

MAZAO GUNIA 10 HADI 15
KWA EKARI MOJA

AINA YA MAHINDI –Ya Pili

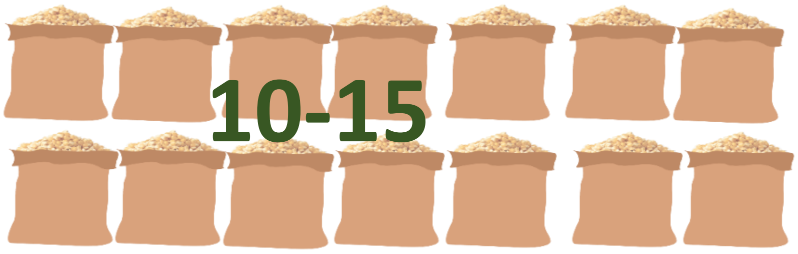

MAZAO GUNIA 10 HADI 15
KWA EKARI MOJA

MBEGU NDOGO-NDOGO

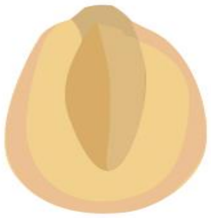

MBEGU AMBAYO NI MAKUBWA KADIRI

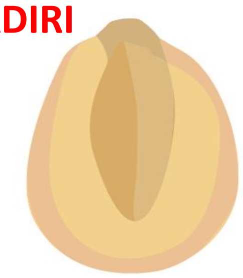

HAIPITI MIEZI 3 KABLA HAIJAKOMAA

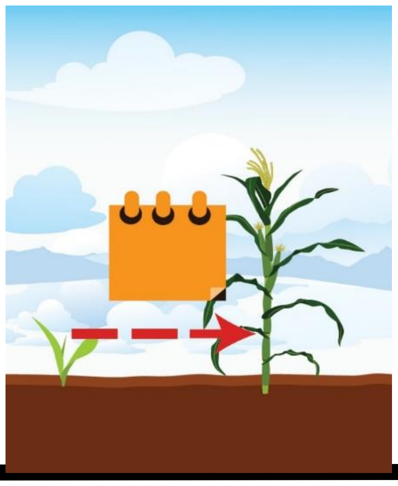

INAKOMAA KWA MIEZI 4 AU ZAIDI

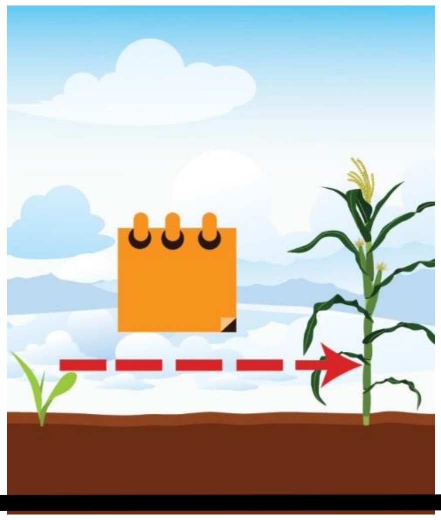

UNAWEZA WEKA BILA KULIWA NA WADUDU HATA UKIWEKA KWA MIEZI 3 HADI 4

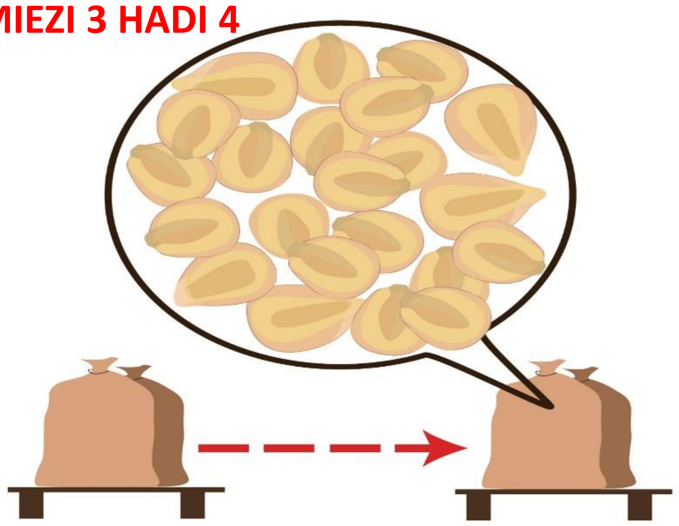

INAKULIWA NA WADUDU IKIKAA MWEZI MOJA AU MBILI

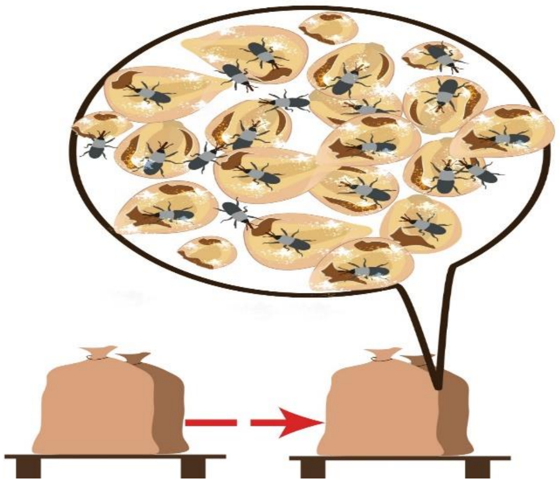

CE-A-Session 3

AINA YA MAHINDI –Ya Kwanza

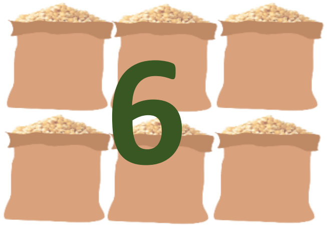

MAZAO GUNIA 6 AU CHINI
KWA EKARI MOJA

MBEGU
MAKUBWA-
MAKUBWA

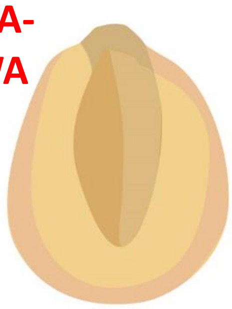

INAKOMAA KWA MIEZI
4 AU ZAIDI

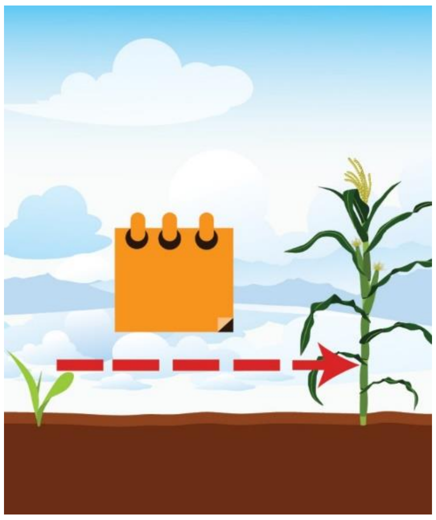

UNAWEZA WEKA BILA KULIWA NA
WADUDU HATA UKIWEKA KWA
MIEZI 3 HADI 4

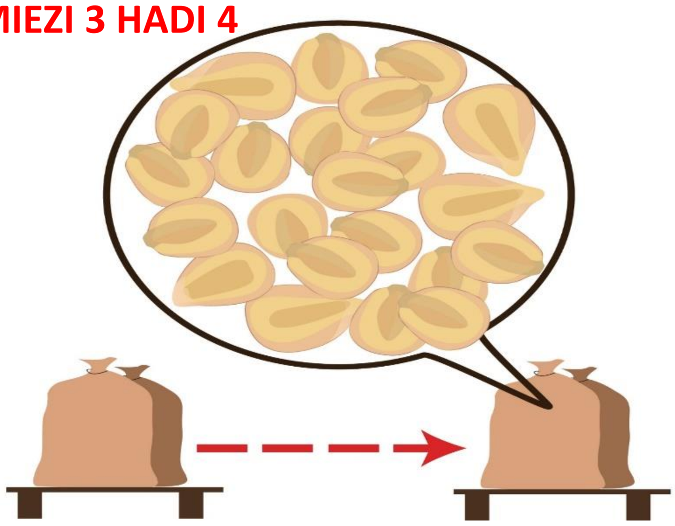

AINA YA MAHINDI –Ya Pili

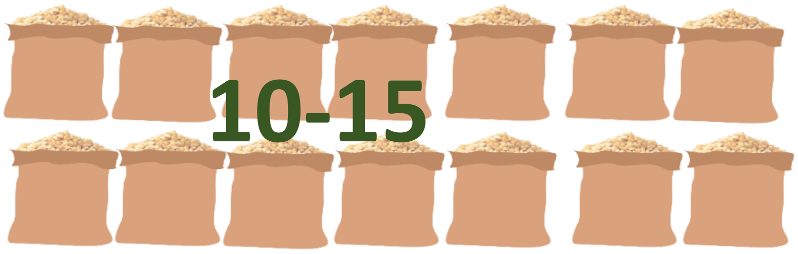

MAZAO GUNIA 10 HADI 15
KWA EKARI MOJA

MBEGU NDOGO-
NDOGO

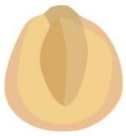

HAIPITI MIEZI 3 KABLA
HAIJAKOMAA

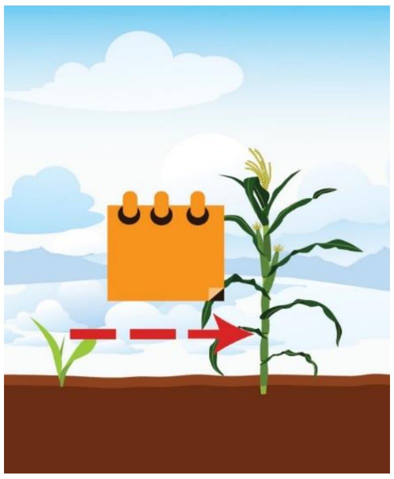

INAKULIWA NA WADUDU
IKIKAA MWEZI MOJA AU MBILI

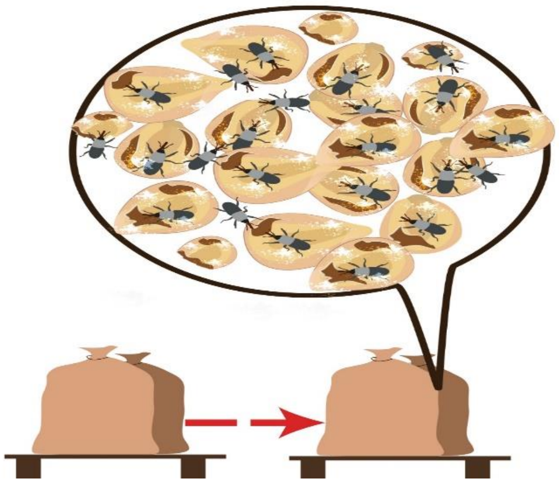

CE-A-Session 5

AINA YA MAHINDI –Ya Kwanza

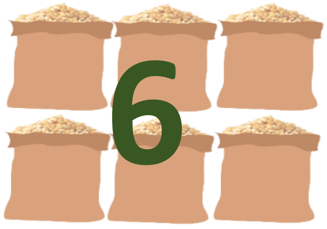

MAZAO GUNIA 6 AU CHINI
KWA EKARI MOJA

MBEGU
MAKUBWA-
MAKUBWA

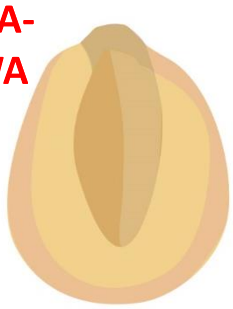

INAKOMAA KWA MIEZI
4 AU ZAIDI

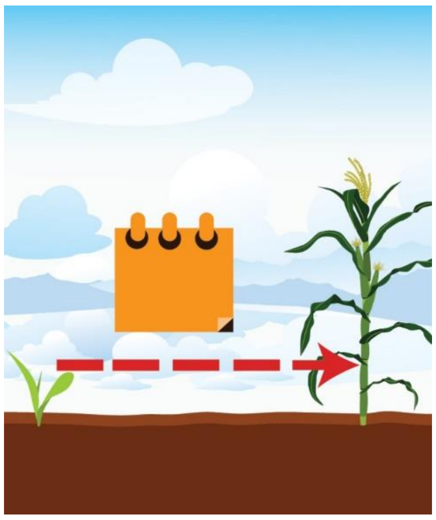

INAKULIWA NA WADUDU
IKIKAA MWEZI MOJA AU MBILI

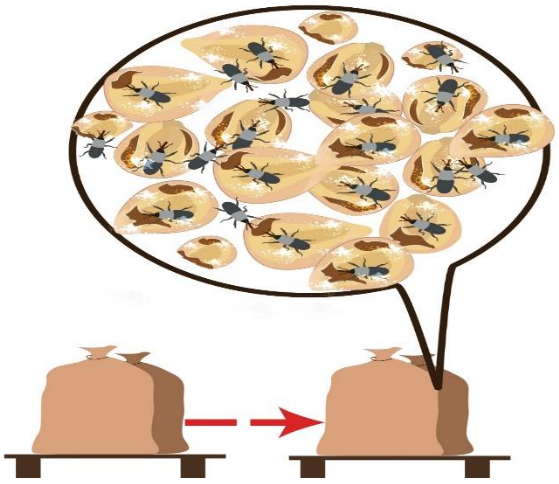

AINA YA MAHINDI –Ya Pili

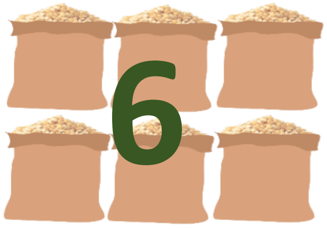

MAZAO GUNIA 6 AU CHINI
KWA EKARI MOJA

MBEGU AMBAYO
NI MAKUBWA
KADIRI

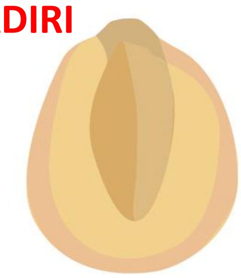

HAIPITI MIEZI 3 KABLA
HAIJAKOMAA

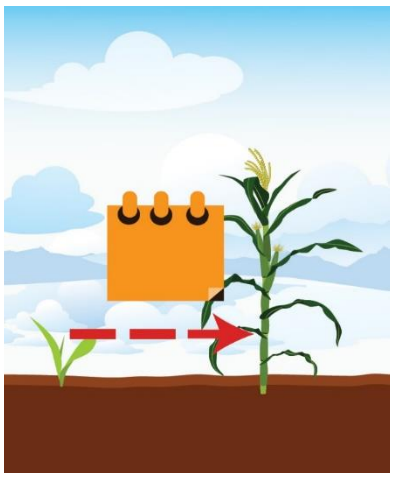

UNAWEZA WEKA BILA KULIWA NA
WADUDU HATA UKIWEKA KWA
MIEZI 3 HADI 4

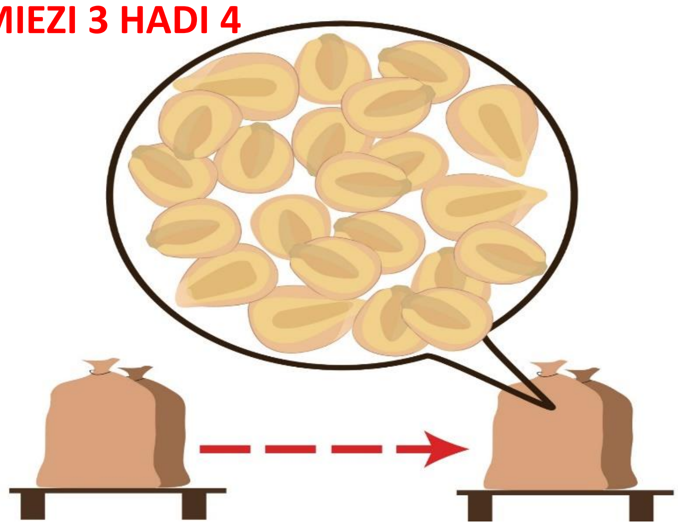

CE-A-Session 6

AINA YA MAHINDI –Ya Kwanza	AINA YA MAHINDI –Ya Pili
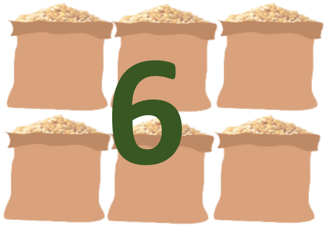 MAZAO GUNIA 6 AU CHINI KWA EKARI MOJA	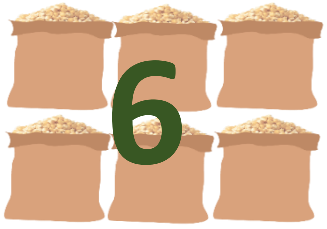 MAZAO GUNIA 6 AU CHINI KWA EKARI MOJA
MBEGU MAKUBWA-MAKUBWA 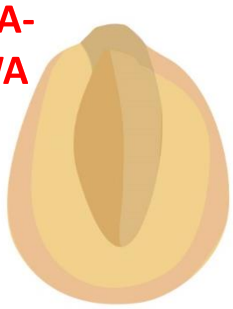	MBEGU AMBAYO NI MAKUBWA KADIRI 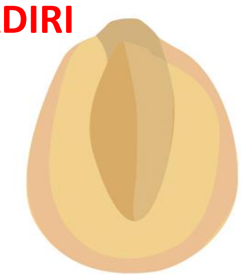
INAKOMAA KWA MIEZI 3 HADI 4 HIVI 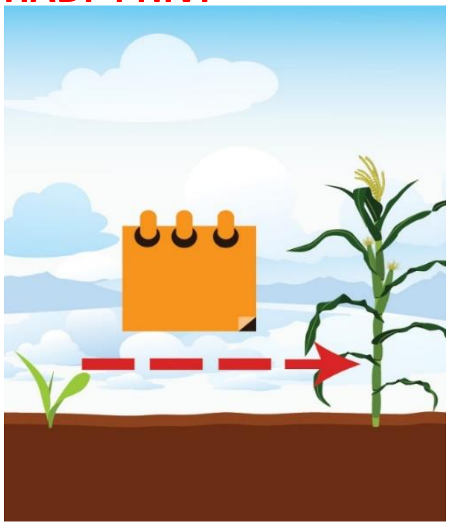	INAKOMAA KWA MIEZI 3 HADI 4 HIVI 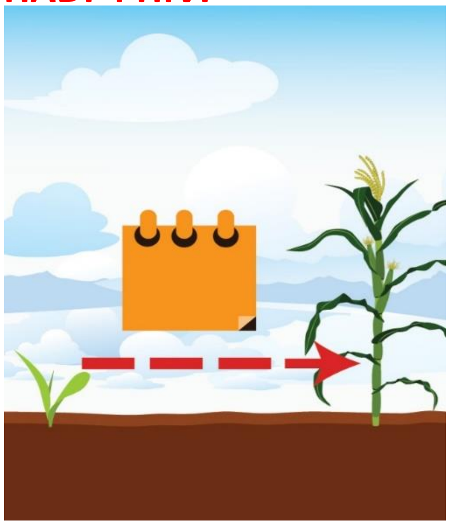
UNAWEZA WEKA BILA KULIWA NA WADUDU HATA UKIWEKA KWA MIEZI 3 HADI 4 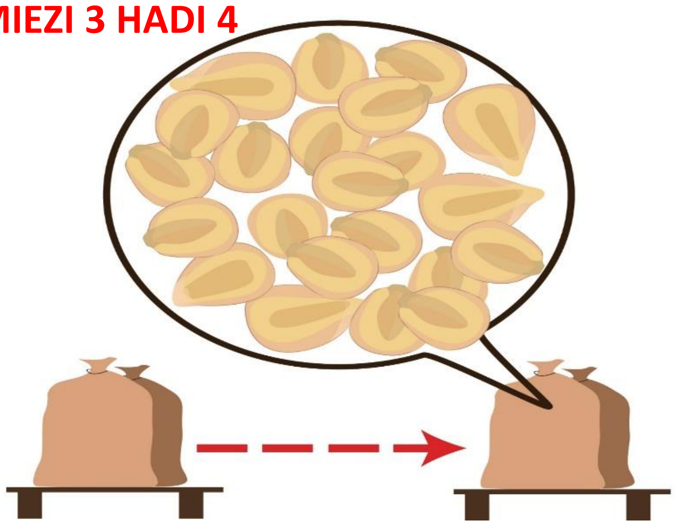	INAKULIWA NA WADUDU IKIKAA MWEZI MOJA AU MBILI 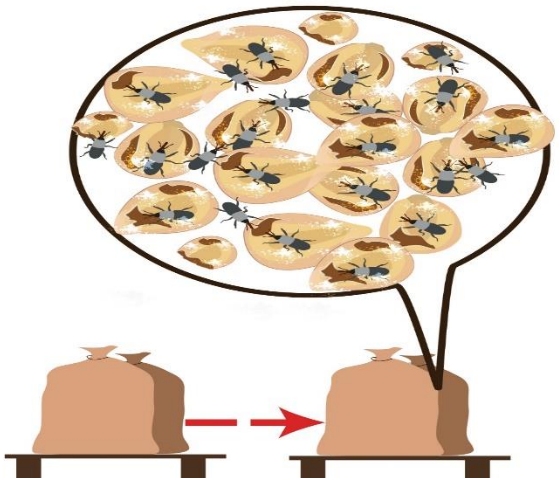

CE-A-Session 6

AINA YA MAHINDI –Ya Kwanza

AINA YA MAHINDI –Ya Pili

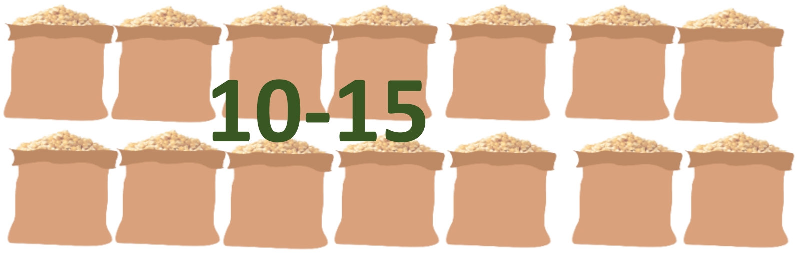

MAZAO GUNIA 10 HADI 15
KWA EKARI MOJA

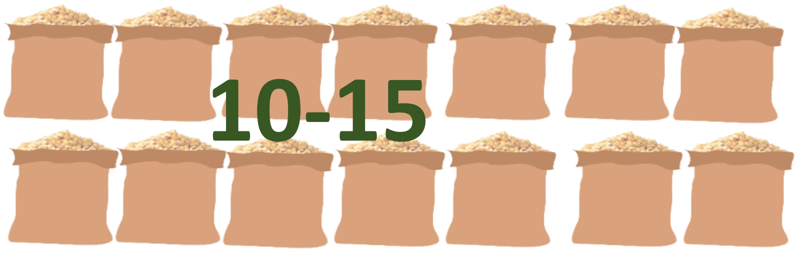

MAZAO GUNIA 10 HADI 15
KWA EKARI MOJA

MBEGU NDOGO-
NDOGO

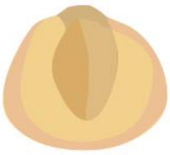

MBEGU AMBAYO
NI MAKUBWA
KADIRI

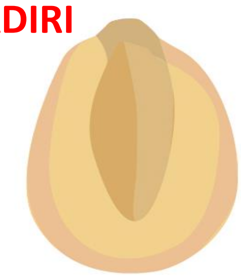

HAIPITI MIEZI 3 KABLA
HAIJAKOMAA

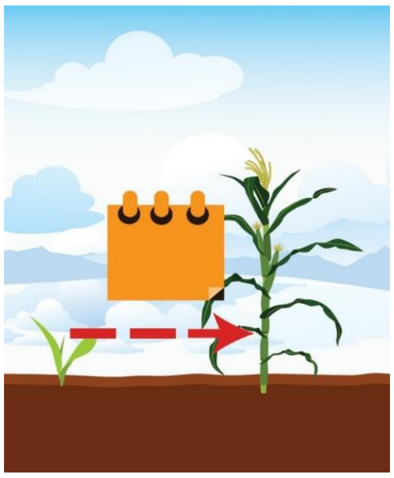

INAKOMAA KWA MIEZI
4 AU ZAIDI

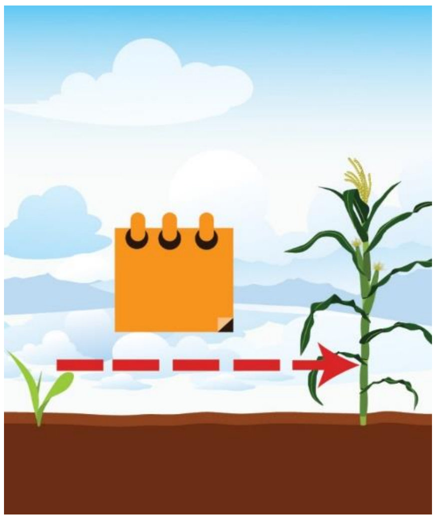

INAKULIWA NA WADUDU
IKIKAA MWEZI MOJA AU MBILI

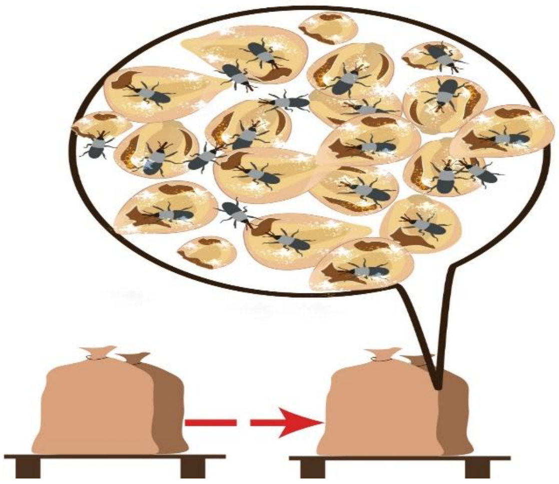

UNAWEZA WEKA BILA KULIWA NA
WADUDU HATA UKIWEKA KWA
MIEZI 3 HADI 4

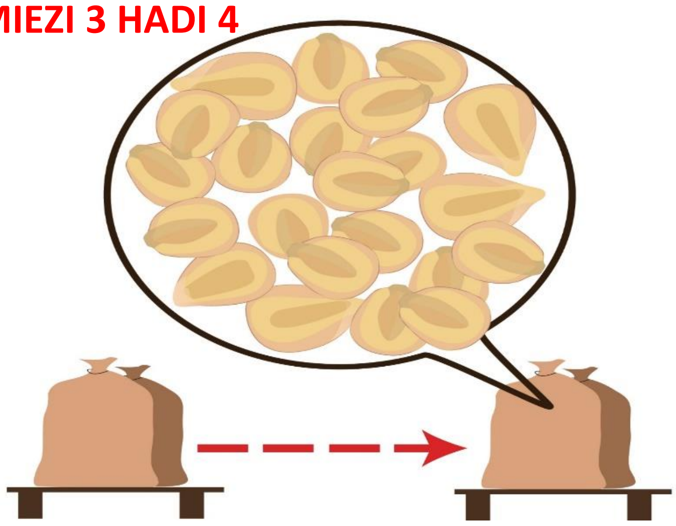

CE-A-Session 7

AINA YA MAHINDI –Ya Kwanza

AINA YA MAHINDI –Ya Pili

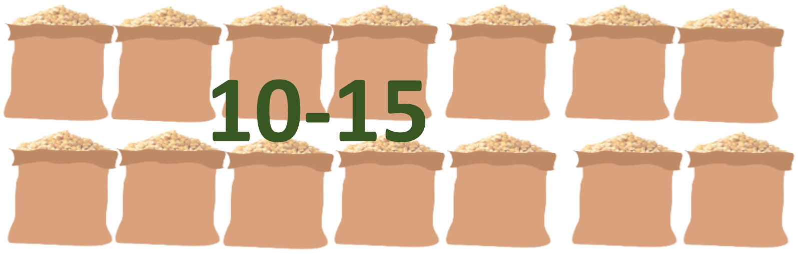

MAZAO GUNIA 10 HADI 15
KWA EKARI MOJA

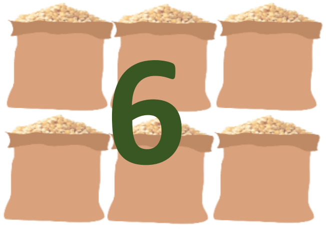

MAZAO GUNIA 6 AU CHINI
KWA EKARI MOJA

MBEGU NDOGO-NDOGO

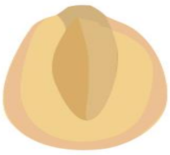

MBEGU MAKUBWA-MAKUBWA

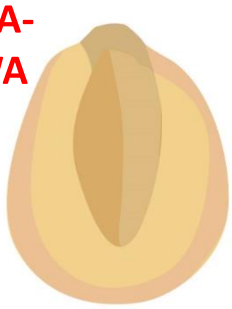

INAKOMAA KWA MIEZI 3 HADI 4 HIVI

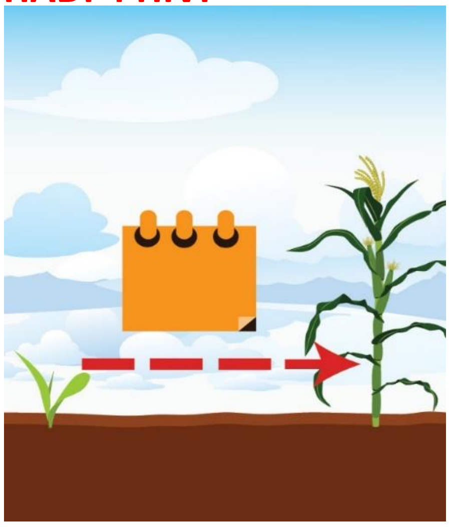

INAKOMAA KWA MIEZI 3 HADI 4 HIVI

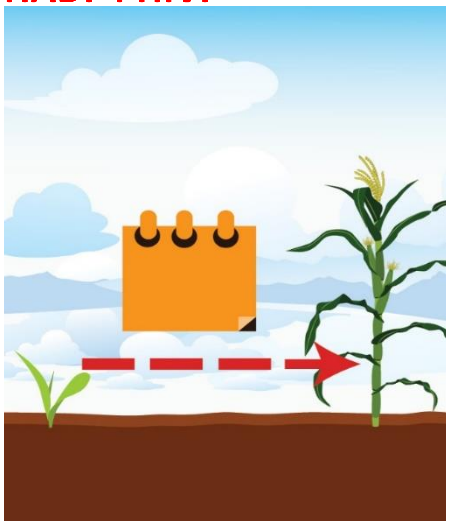

INAKULIWA NA WADUDU IKIKAA MWEZI MOJA AU MBILI

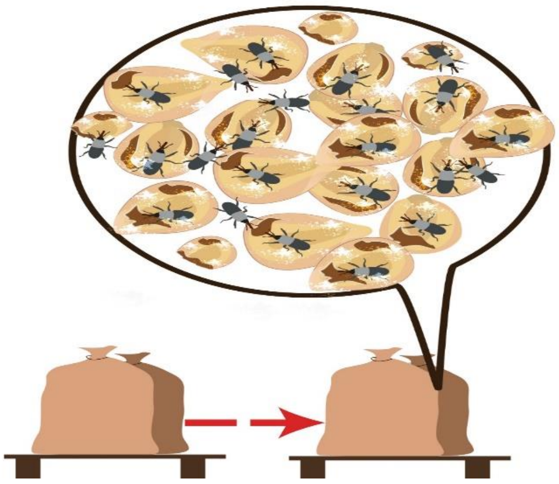

UNAWEZA WEKA BILA KULIWA NA WADUDU HATA UKIWEKA KWA MIEZI 3 HADI 4

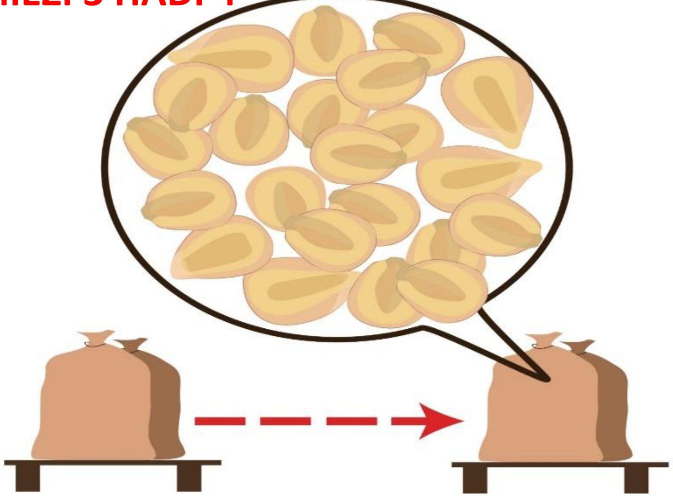

CE-A-Session 8

AINA YA MAHINDI –Ya Kwanza

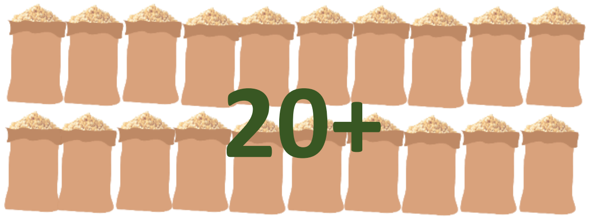

INATOA MAZAO
GUNIA 20 NA ZAIDI
KWA EKARI MOJA

MBEGU
MAKUBWA-
MAKUBWA

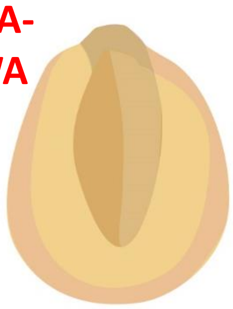

HAIPITI MIEZI 3 KABLA
HAIJAKOMAA

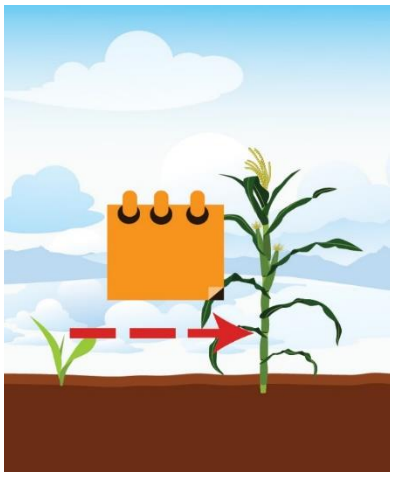

UNAWEZA WEKA BILA KULIWA NA
WADUDU HATA UKIWEKA KWA
MIEZI 3 HADI 4

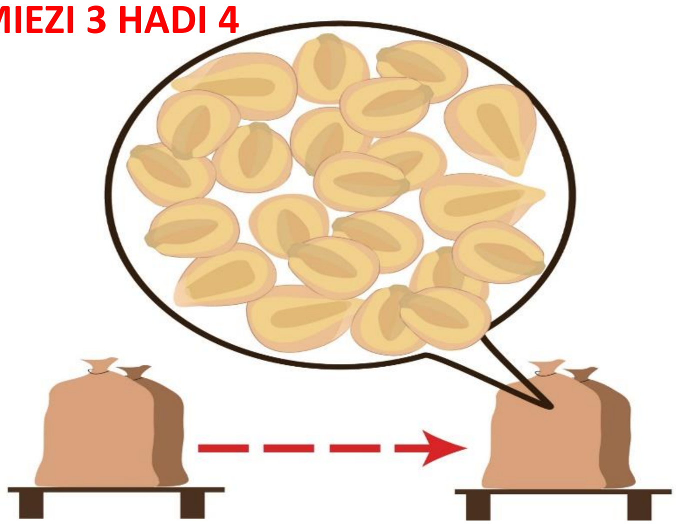

AINA YA MAHINDI –Ya Pili

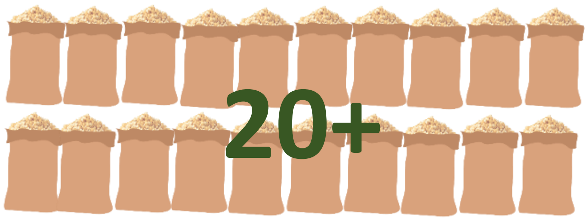

INATOA MAZAO
GUNIA 20 NA ZAIDI
KWA EKARI MOJA

MBEGU AMBAYO
NI MAKUBWA
KADIRI

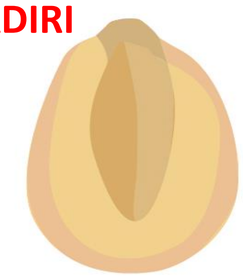

INAKOMAA KWA MIEZI
4 AU ZAIDI

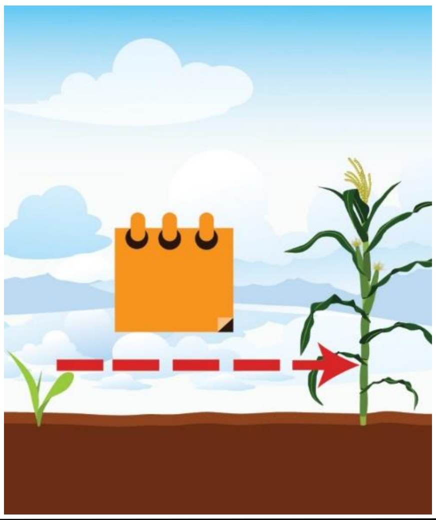

INAKULIWA NA WADUDU
IKIKAA MWEZI MOJA AU MBILI

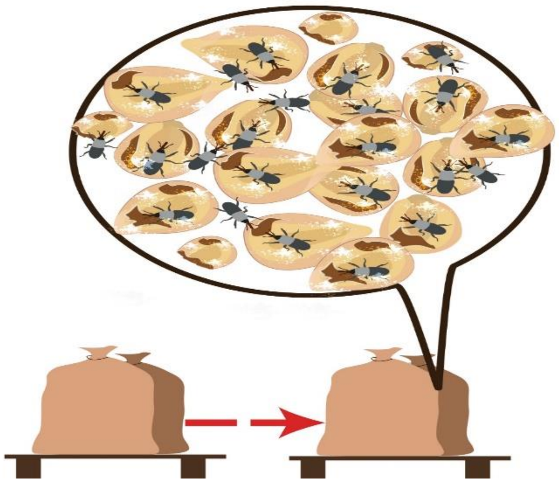

CE-A-Session 9

AINA YA MAHINDI –Ya Kwanza

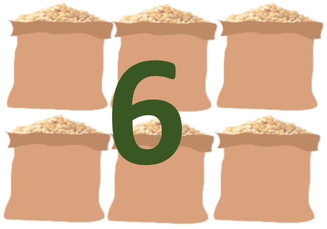

MAZAO GUNIA 6 AU CHINI KWA EKARI MOJA

MBEGU MAKUBWA-MAKUBWA

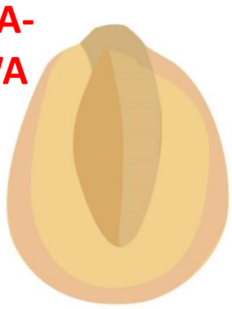

INAKOMAA KWA MIEZI 3 HADI 4 HIVI

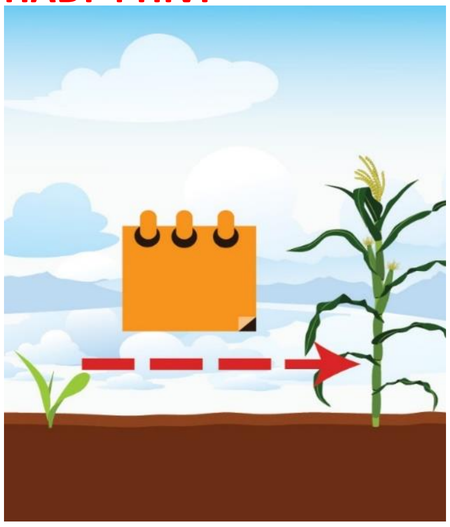

UNAWEZA WEKA BILA KULIWA NA WADUDU HATA UKIWEKA KWA MIEZI 3 HADI 4

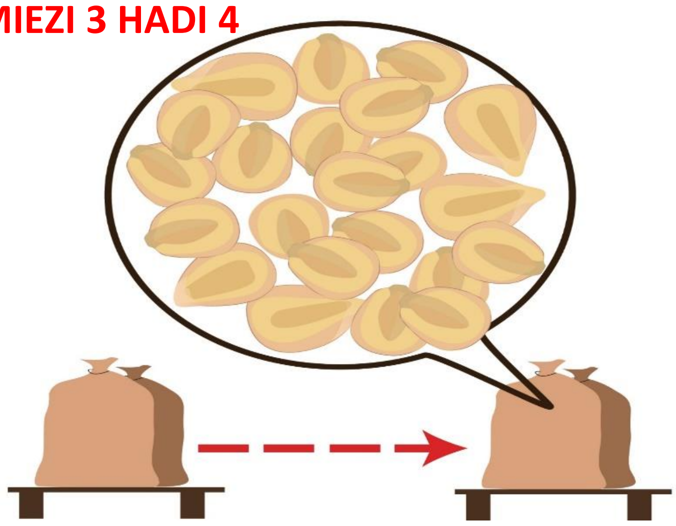

AINA YA MAHINDI –Ya Pili

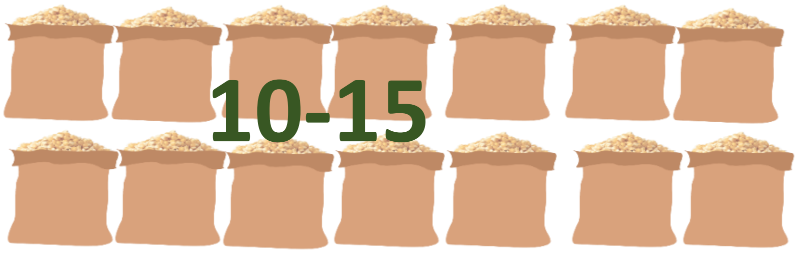

MAZAO GUNIA 10 HADI 15 KWA EKARI MOJA

MBEGU NDOGO-NDOGO

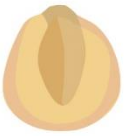

INAKOMAA KWA MIEZI 3 HADI 4 HIVI

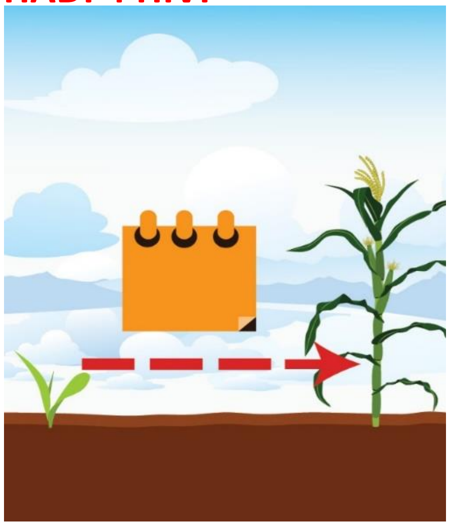

INAKULIWA NA WADUDU IKIKAA MWEZI MOJA AU MBILI

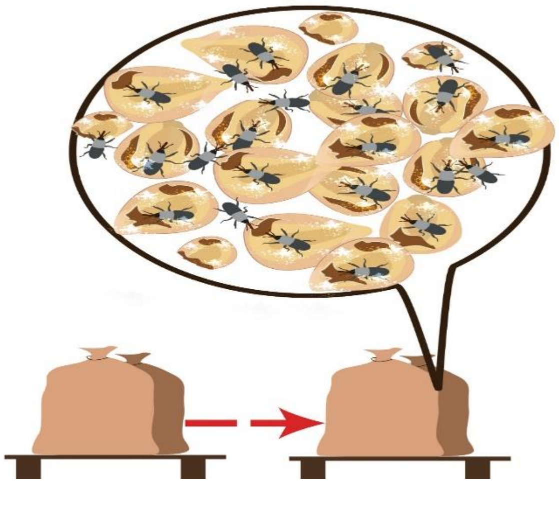

CE-A-Session 10

AINA YA MAHINDI –Ya Kwanza

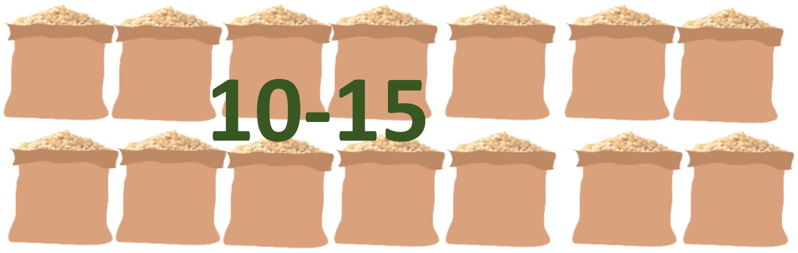

MAZAO GUNIA 10 HADI 15
KWA EKARI MOJA

MBEGU AMBAYO
NI MAKUBWA
KADIRI

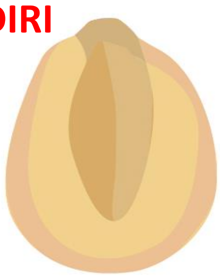

INAKOMAA KWA MIEZI 3
HADI 4 HIVI

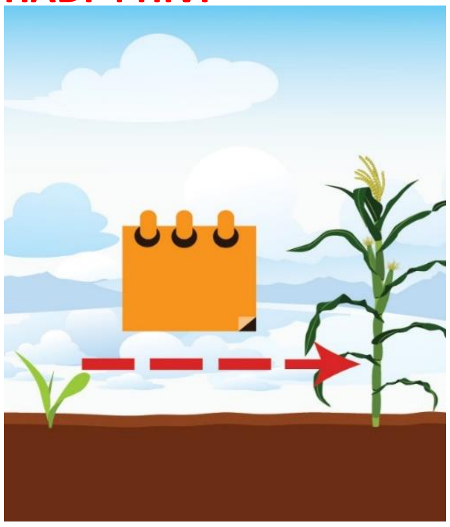

INAKULIWA NA WADUDU
IKIKAA MWEZI MOJA AU MBILI

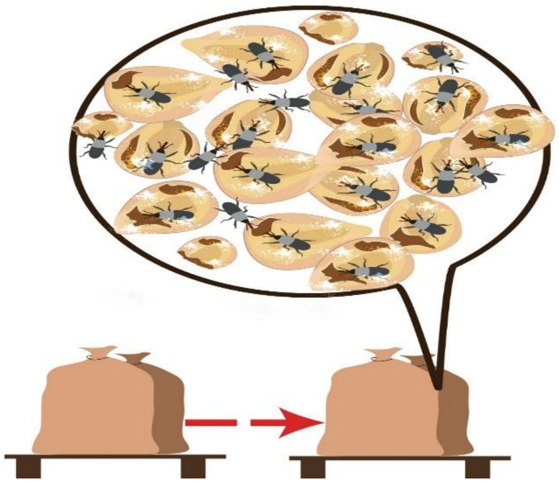

AINA YA MAHINDI –Ya Pili

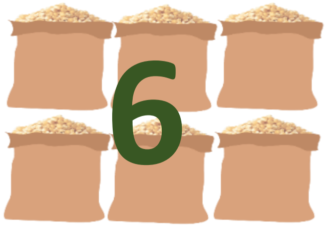

MAZAO GUNIA 6 AU CHINI
KWA EKARI MOJA

MBEGU
MAKUBWA-
MAKUBWA

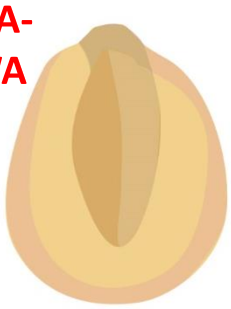

INAKOMAA KWA MIEZI 3
HADI 4 HIVI

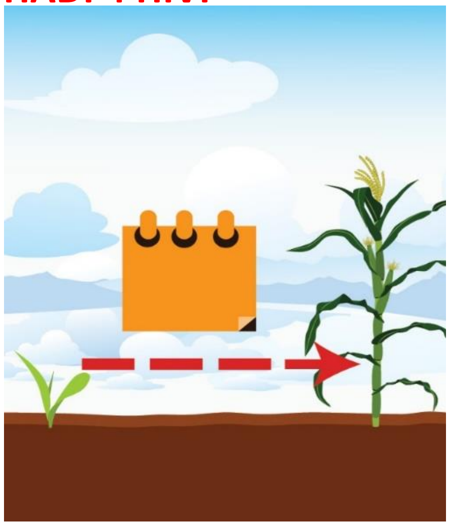

UNAWEZA WEKA BILA KULIWA NA
WADUDU HATA UKIWEKA KWA
MIEZI 3 HADI 4

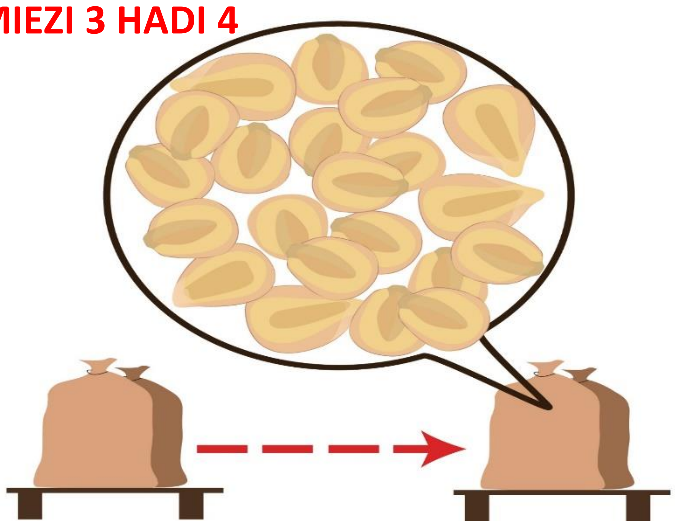

CE-A-Session 11

AINA YA MAHINDI –Ya Kwanza

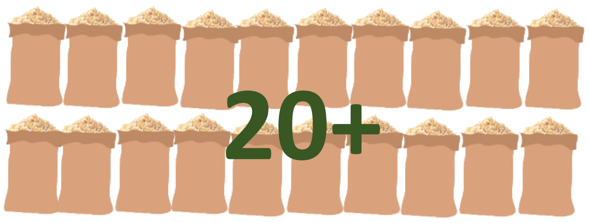

INATOA MAZAO
GUNIA 20 NA ZAIDI
KWA EKARI MOJA

MBEGU AMBAYO
NI MAKUBWA
KADIRI

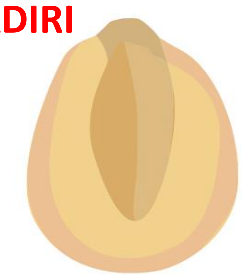

HAIPITI MIEZI 3 KABLA
HAIJAKOMAA

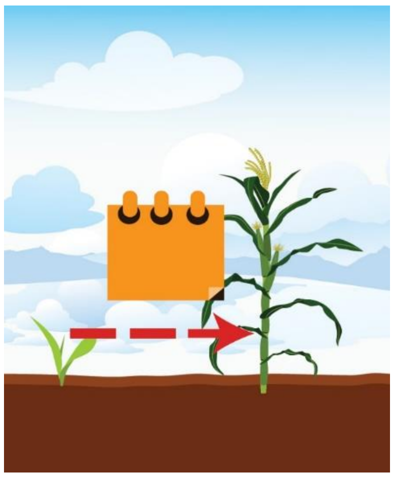

INAKULIWA NA WADUDU
IKIKAA MWEZI MOJA AU MBILI

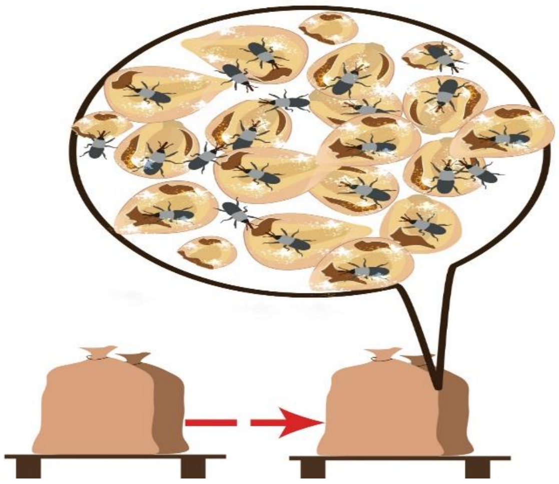

AINA YA MAHINDI –Ya Pili

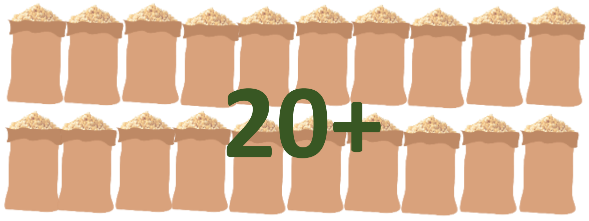

INATOA MAZAO
GUNIA 20 NA ZAIDI
KWA EKARI MOJA

MBEGU NDOGO-
NDOGO

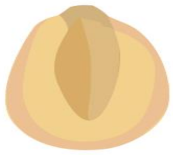

INAKOMAA KWA MIEZI
4 AU ZAIDI

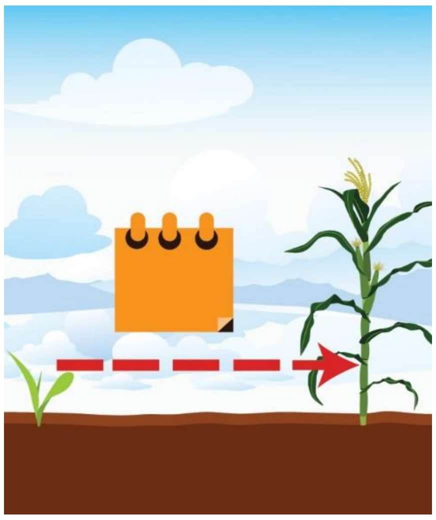

UNAWEZA WEKA BILA KULIWA NA
WADUDU HATA UKIWEKA KWA
MIEZI 3 HADI 4

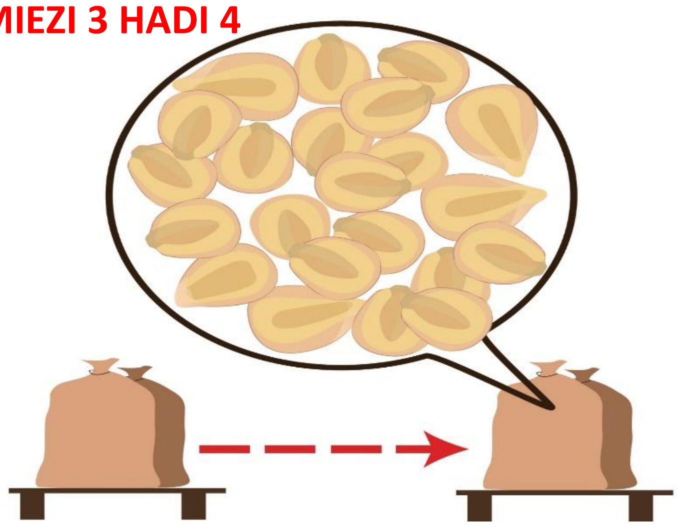

CE-A-Session 11

AINA YA MAHINDI –Ya Kwanza

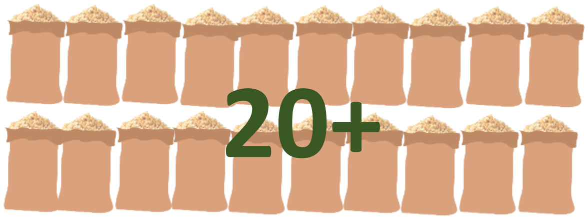

INATOA MAZAO
GUNIA 20 NA ZAIDI
KWA EKARI MOJA

MBEGU AMBAYO
NI MAKUBWA
KADIRI

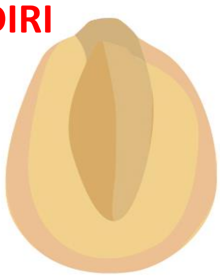

INAKOMAA KWA MIEZI
4 AU ZAIDI

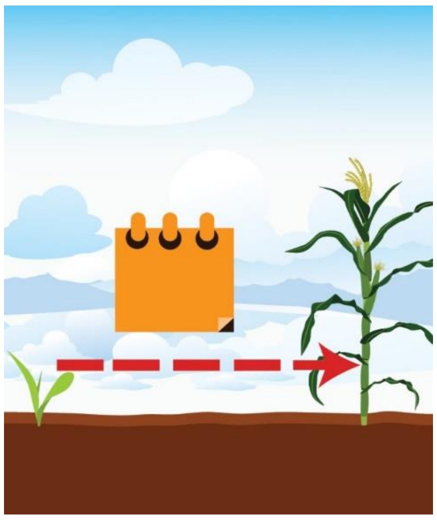

INAKULIWA NA WADUDU
IKIKAA MWEZI MOJA AU MBILI

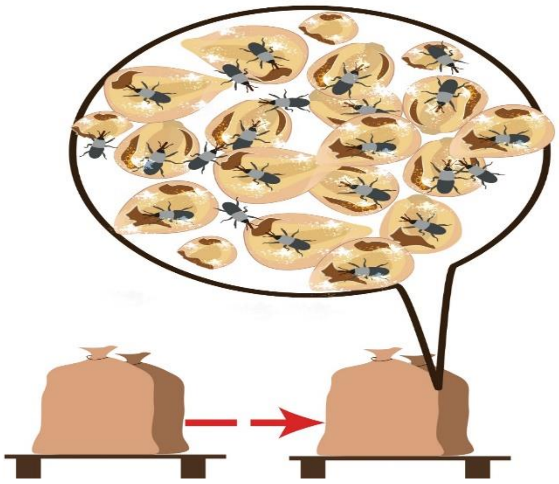

AINA YA MAHINDI –Ya Pili

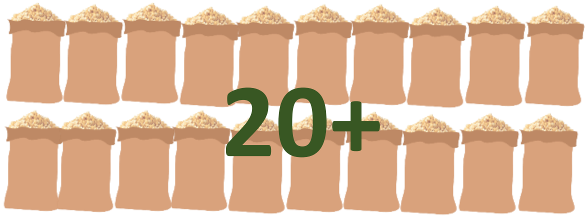

INATOA MAZAO
GUNIA 20 NA ZAIDI
KWA EKARI MOJA

MBEGU NDOGO-
NDOGO

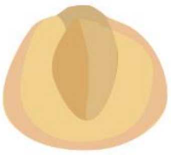

HAIPITI MIEZI 3 KABLA
HAIJAKOMAA

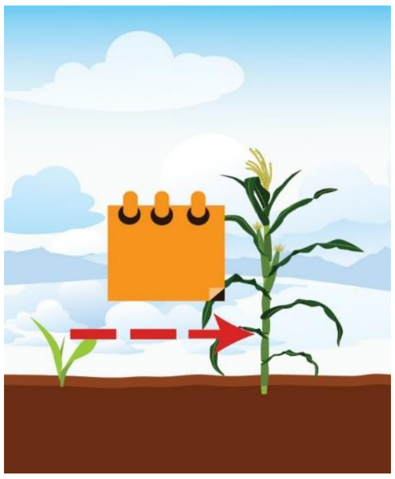

UNAWEZA WEKA BILA KULIWA NA
WADUDU HATA UKIWEKA KWA
MIEZI 3 HADI 4

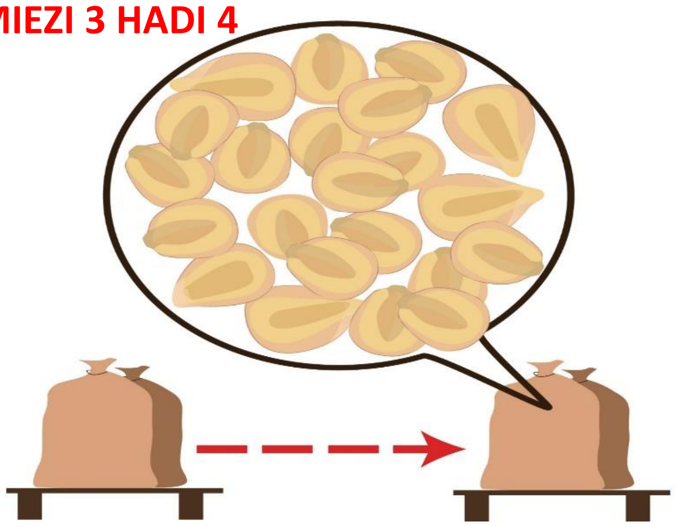

Supplement: Kenya-Choce-Experiment-A.pdf [file mmc5.pdf]
