# Supplementary material for: Trait preference trade-offs among maize farmers in western Kenya
Source: Heliyon. 2021 Mar 12;7(3):e06389. doi: 10.1016/j.heliyon.2021.e06389 (PMC7970324; doi:10.1016/j.heliyon.2021.e06389)
Supplement: Kenya-Choce-Experiment-B.pdf [file mmc6.pdf]

CE-B-Session 1

AINA YA MAHINDI –Ya Kwanza

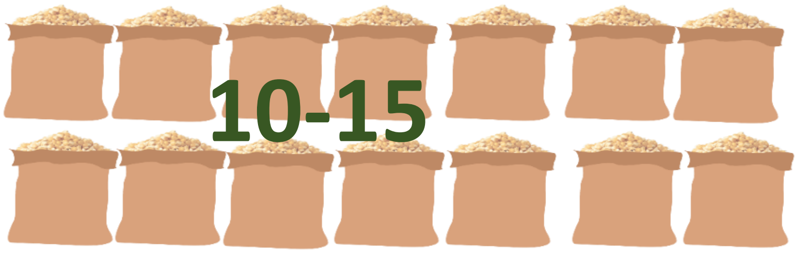

MAZAO GUNIA 10 HADI 15
KWA EKARI MOJA

MBEGU
NYEPESI-NYEPESI

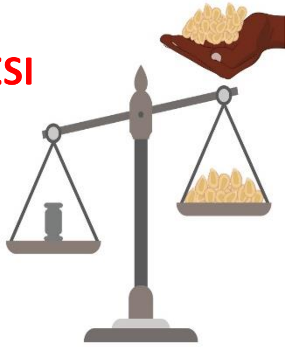

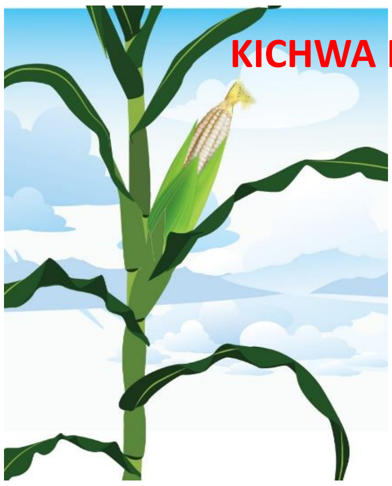

KICHWA KIKO WAZI

KINAHITAJI MIFUKO MINGI YA MBOLEA YA TOP DRESSING

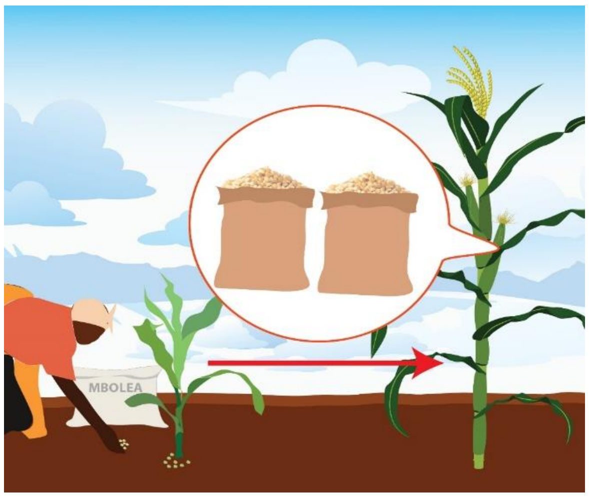

AINA YA MAHINDI –Ya Pili

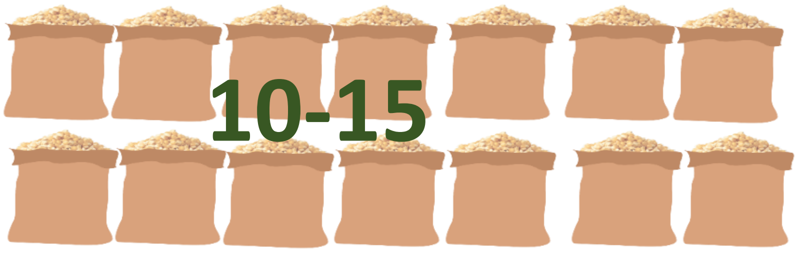

MAZAO GUNIA 10 HADI 15
KWA EKARI MOJA

MBEGU
NZITO-NZITO

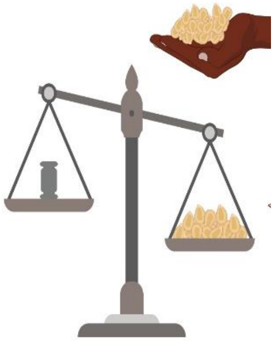

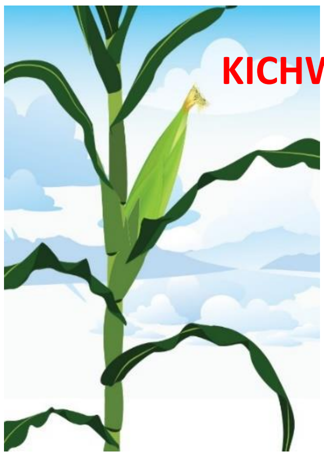

KICHWA KIMEFUNGA

KINATOSHEKA NA MIFUKO CHACHE YA MBOLEA YA TOP DRESSING

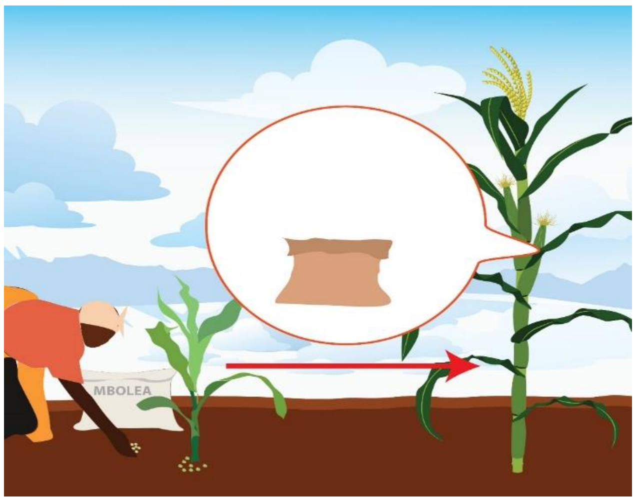

CE-B-Session 1

AINA YA MAHINDI –Ya Kwanza

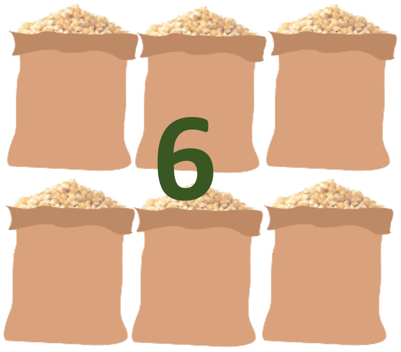

MAZAO GUNIA 6 AU CHINI KWA EKARI MOJA

MBEGU
NYEPESI-NYEPESI

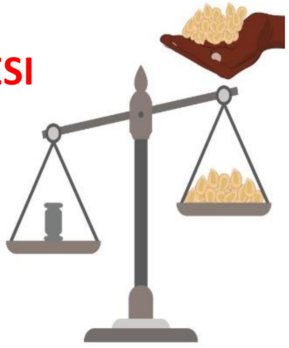

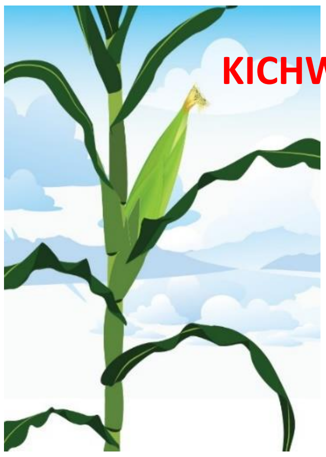

KICHWA KIMEFUNGA

KINAHITAJI MIFUKO MINGI YA MBOLEA YA TOP DRESSING

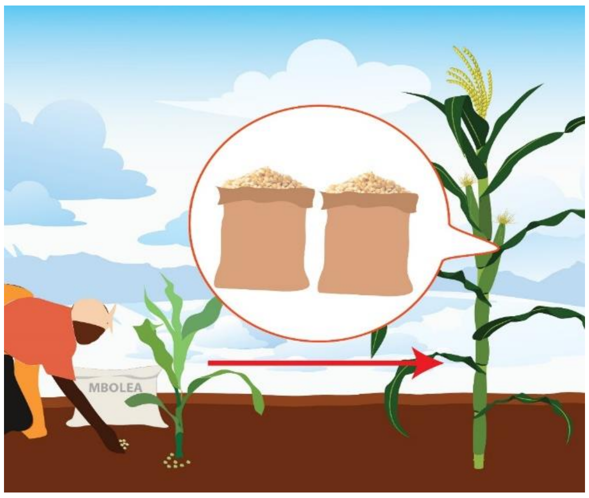

AINA YA MAHINDI –Ya Pili

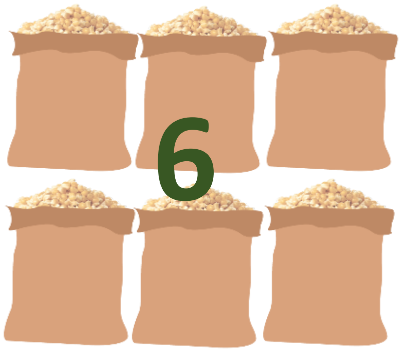

MAZAO GUNIA 6 AU CHINI KWA EKARI MOJA

MBEGU
NZITO-NZITO

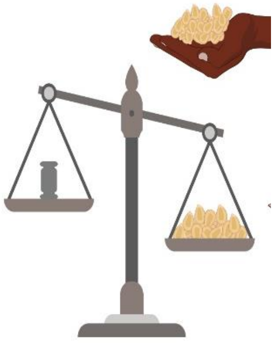

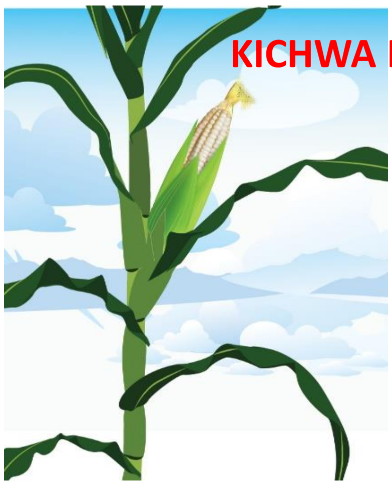

KICHWA KIKO WAZI

KINATOSHEKA NA MIFUKO CHACHE YA MBOLEA YA TOP DRESSING

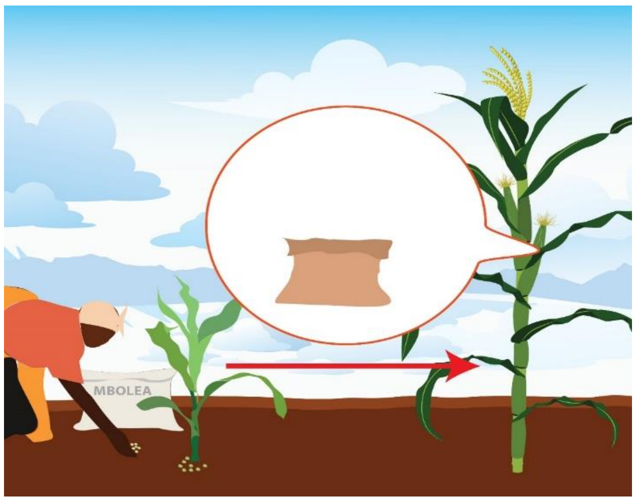

CE-B-Session 3

AINA YA MAHINDI –Ya Kwanza

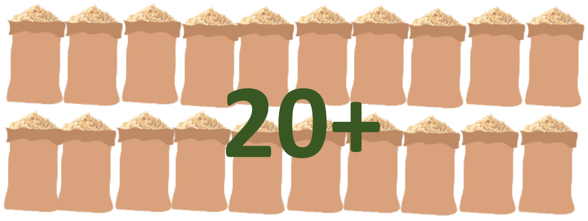

INATOA MAZAO
GUNIA 20 NA ZAIDI
KWA EKARI MOJA

MBEGU
NYEPESI-NYEPESI

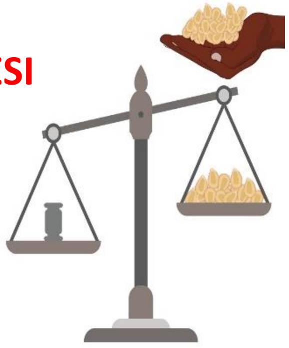

KICHWA KIKO WAZI

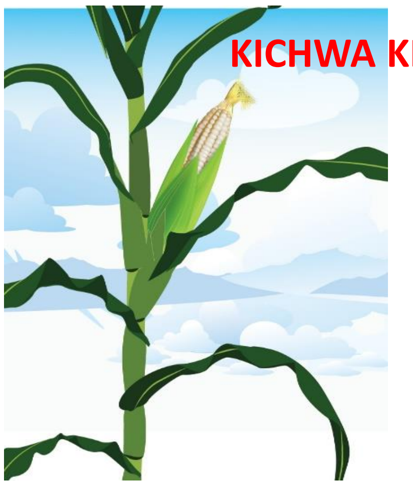

KINATOSHEKA NA MIFUKO CHACHE YA MBOLEA YA TOP DRESSING

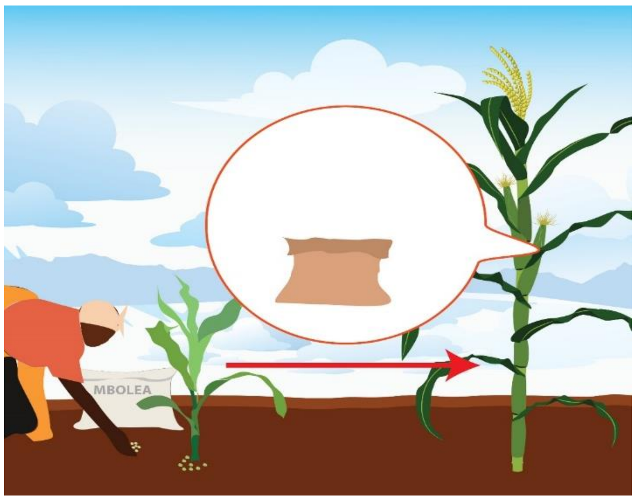

AINA YA MAHINDI –Ya Pili

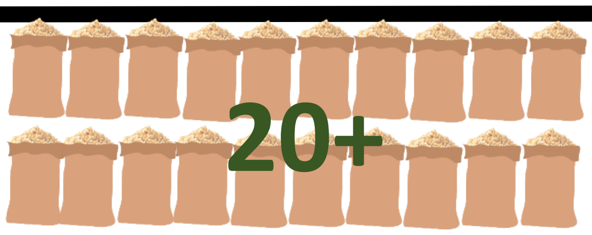

INATOA MAZAO
GUNIA 20 NA ZAIDI
KWA EKARI MOJA

MBEGU
NZITO-NZITO

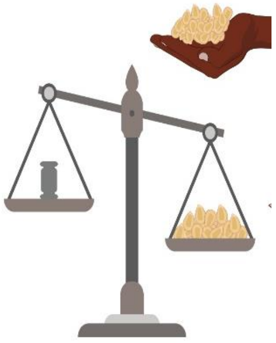

KICHWA KIMEFUNGA

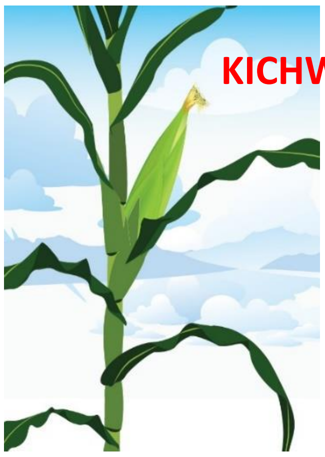

KINAHITAJI MIFUKO MINGI YA MBOLEA YA TOP DRESSING

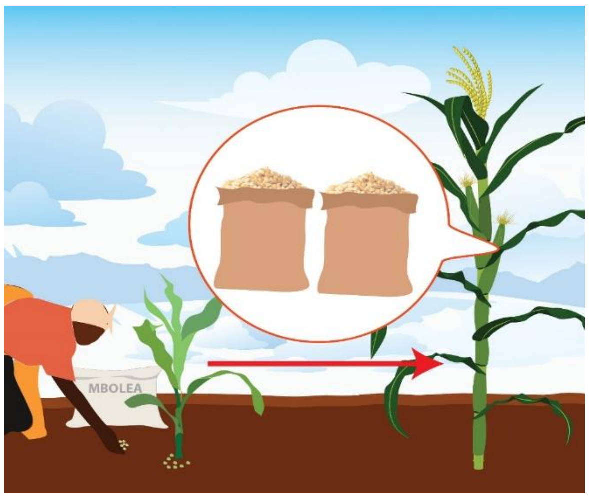

CE-B-Session 4

AINA YA MAHINDI –Ya Kwanza

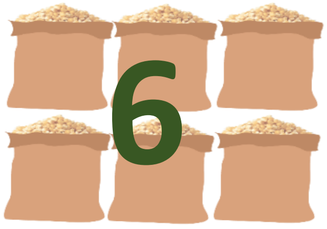

MAZAO GUNIA 6 AU CHINI
KWA EKARI MOJA

MBEGU
NZITO-NZITO

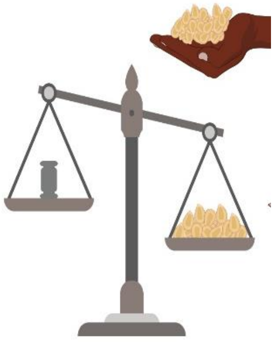

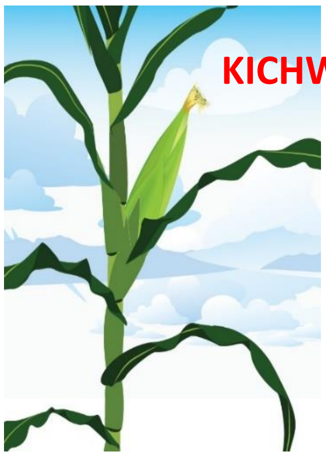

KICHWA KIMEFUNGA

KINAHITAJI MIFUKO MINGI YA MBOLEA YA TOP DRESSING

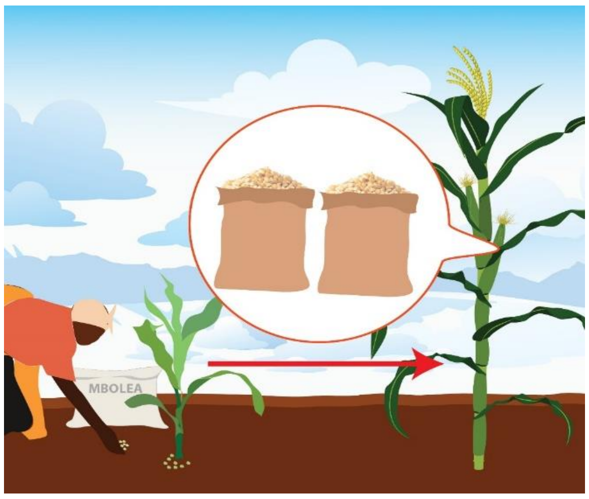

AINA YA MAHINDI –Ya Pili

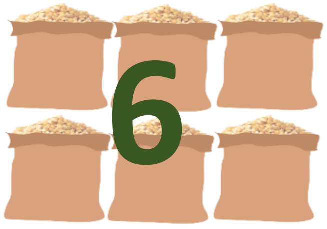

MAZAO GUNIA 6 AU CHINI
KWA EKARI MOJA

MBEGU
NYEPESI-NYEPESI

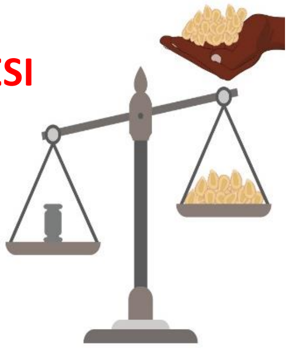

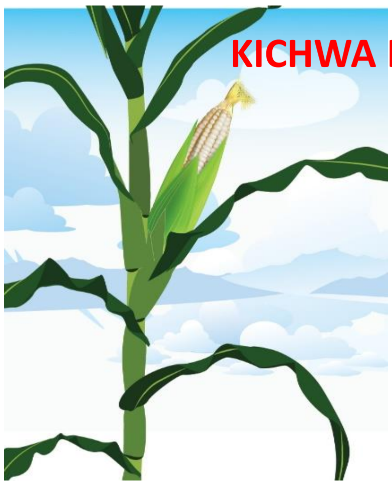

KICHWA KIKO WAZI

KINATOSHEKA NA MIFUKO CHACHE YA MBOLEA YA TOP DRESSING

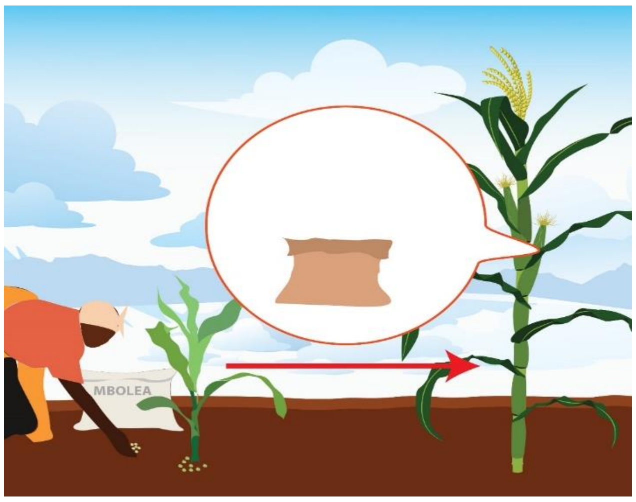

CE-B-Session 5

AINA YA MAHINDI –Ya Kwanza

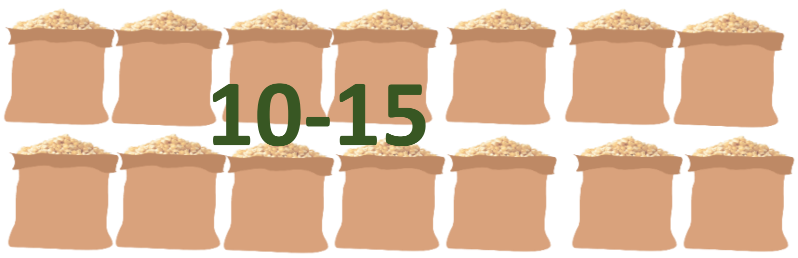

MAZAO GUNIA 10 HADI 15
KWA EKARI MOJA

MBEGU
NZITO-NZITO

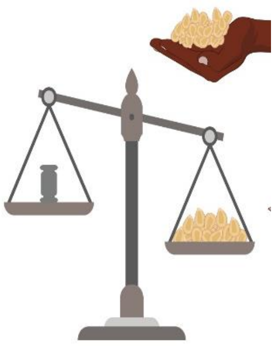

KICHWA KIKO WAZI

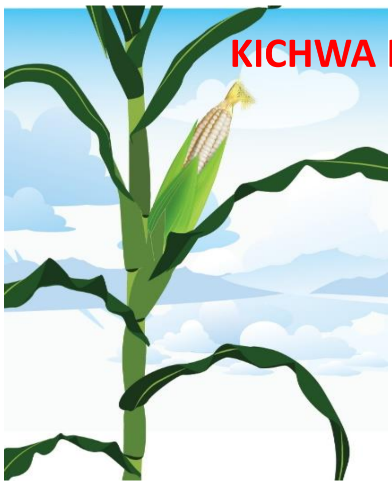

KINATOSHEKA NA MIFUKO CHACHE YA MBOLEA YA TOP DRESSING

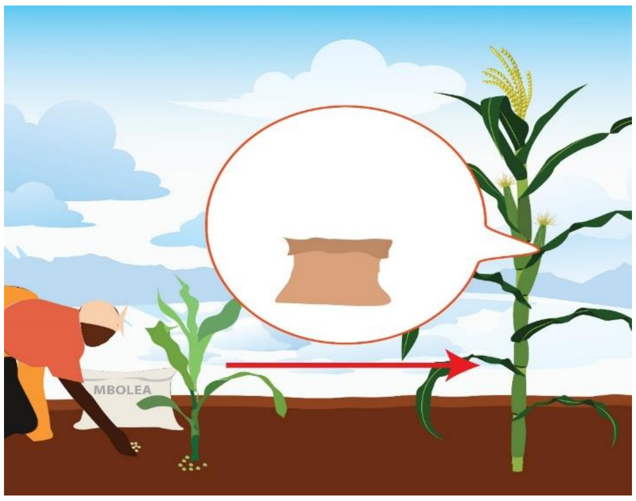

AINA YA MAHINDI –Ya Pili

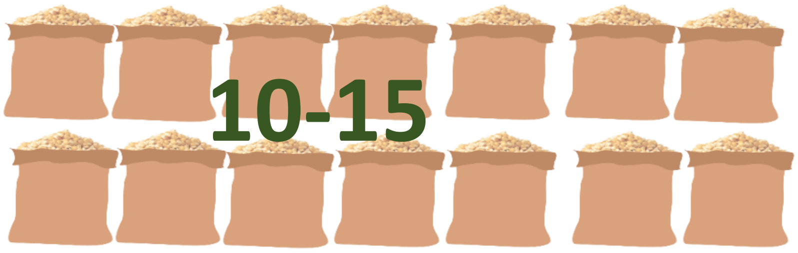

MAZAO GUNIA 10 HADI 15
KWA EKARI MOJA

MBEGU
NYEPESI-NYEPESI

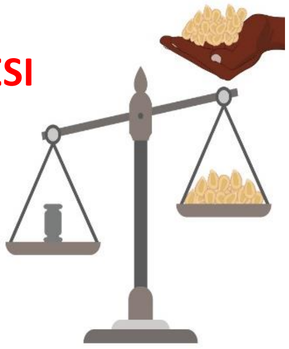

KICHWA KIMEFUNGA

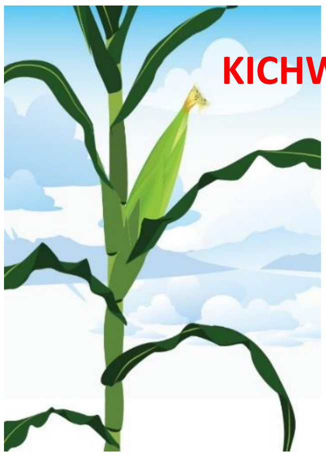

KINAHITAJI MIFUKO MINGI YA MBOLEA YA TOP DRESSING

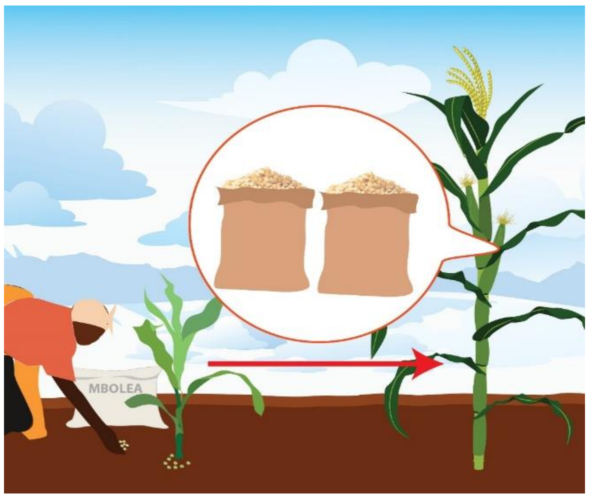

CE-B-Session 6

AINA YA MAHINDI –Ya Kwanza

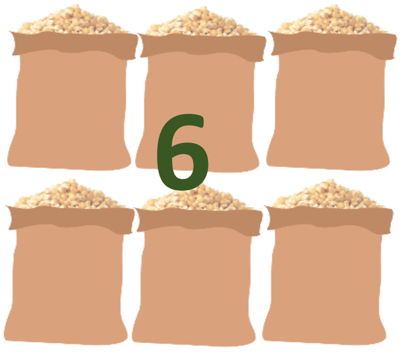

MAZAO GUNIA 6 AU CHINI KWA EKARI MOJA

MBEGU
NZITO-NZITO

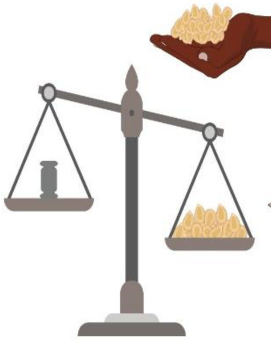

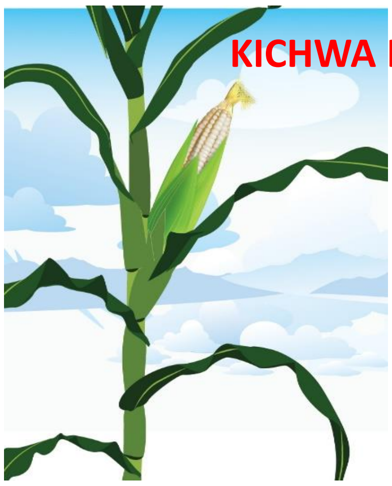

KICHWA KIKO WAZI

KINAHITAJI MIFUKO MINGI YA MBOLEA YA TOP DRESSING

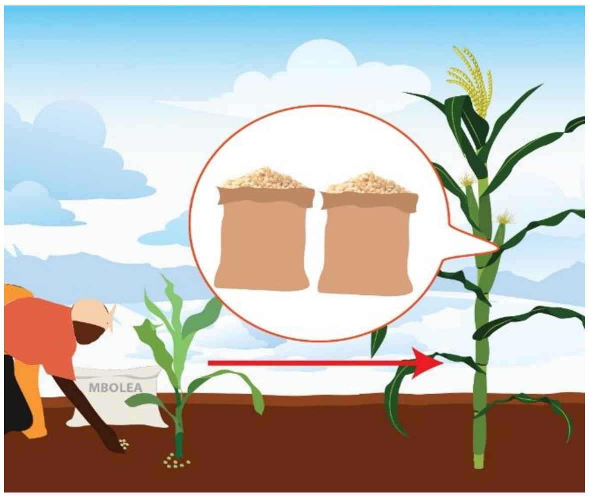

AINA YA MAHINDI –Ya Pili

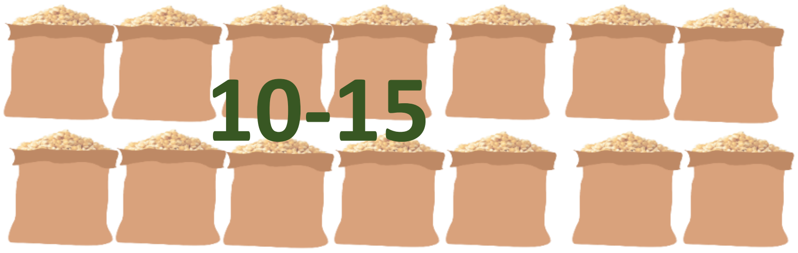

MAZAO GUNIA 10 HADI 15 KWA EKARI MOJA

MBEGU
NYEPESI-NYEPESI

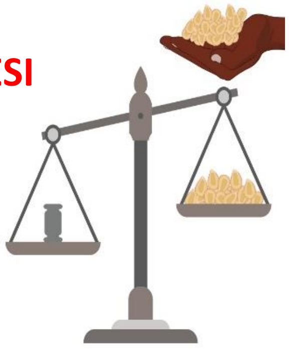

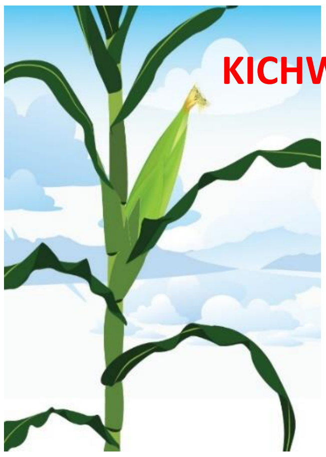

KICHWA KIMEFUNGA

KINATOSHEKA NA MIFUKO CHACHE YA MBOLEA YA TOP DRESSING

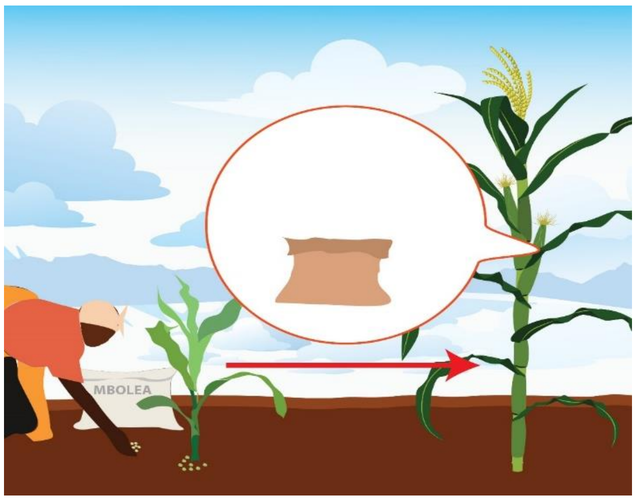

AINA YA MAHINDI –Ya Kwanza

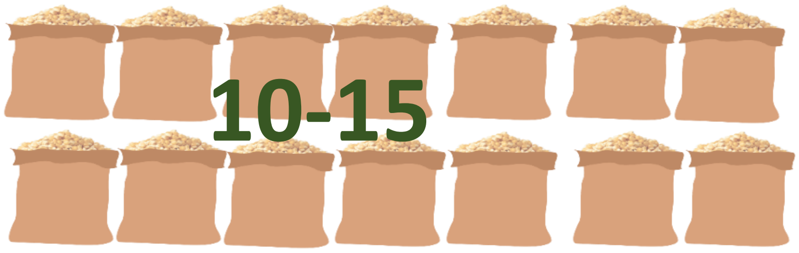

MAZAO GUNIA 10 HADI 15
KWA EKARI MOJA

MBEGU
NYEPESI-NYEPESI

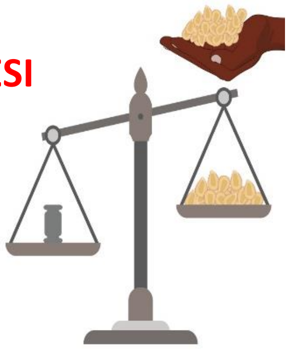

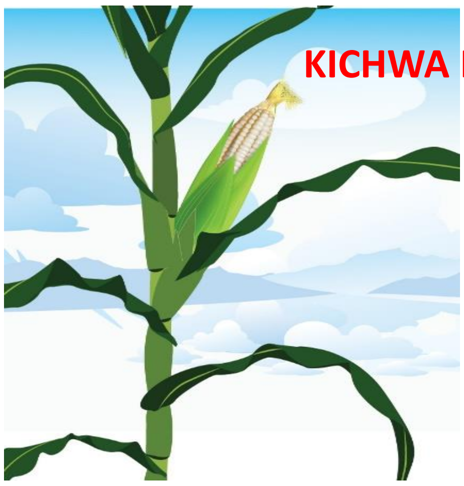

KICHWA KIKO WAZI

KINATOSHEKA NA MIFUKO CHACHE YA MBOLEA YA TOP DRESSING

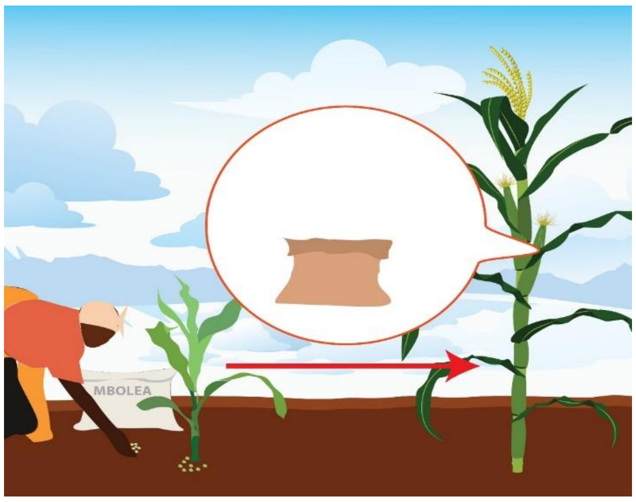

AINA YA MAHINDI –Ya Pili

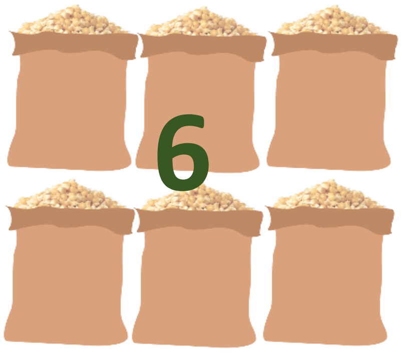

MAZAO GUNIA 6 AU CHINI KWA
EKARI MOJA

MBEGU
NZITO-NZITO

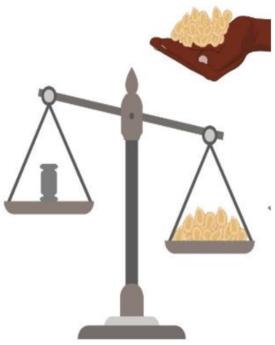

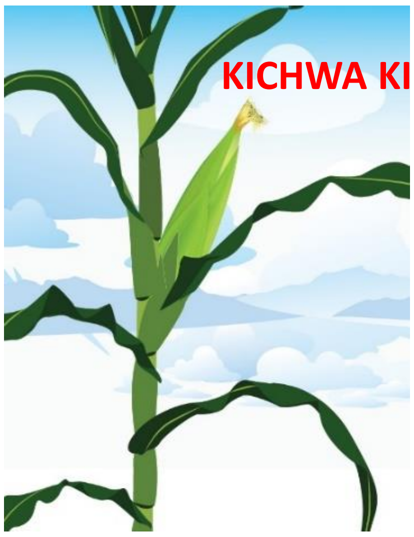

KICHWA KIMEFUNGA

KINAHITAJI MIFUKO MINGI YA MBOLEA YA TOP DRESSING

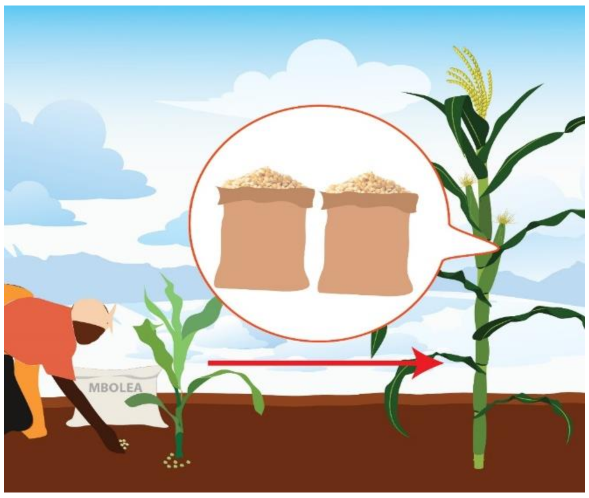

CE-B-Session 8

AINA YA MAHINDI –Ya Kwanza

AINA YA MAHINDI –Ya Pili

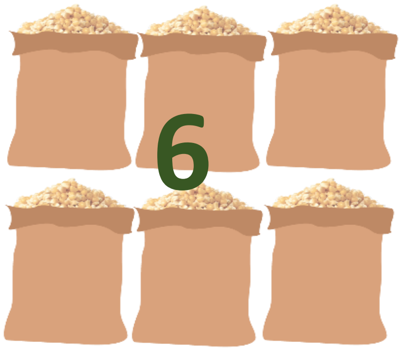

MAZAO GUNIA 6 AU CHINI KWA EKARI MOJA

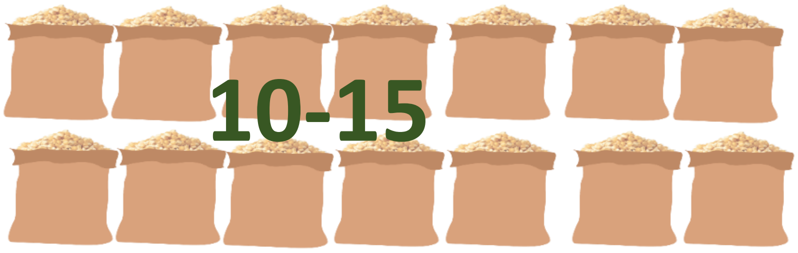

MAZAO GUNIA 10 HADI 15 KWA EKARI MOJA

MBEGU NZITO-NZITO

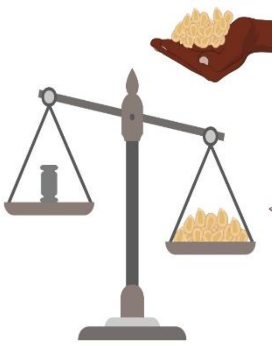

MBEGU NYEPESI-NYEPESI

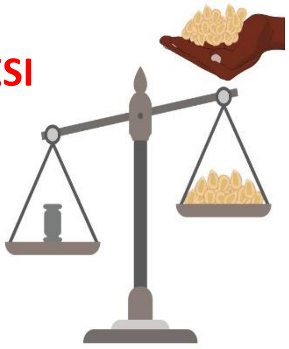

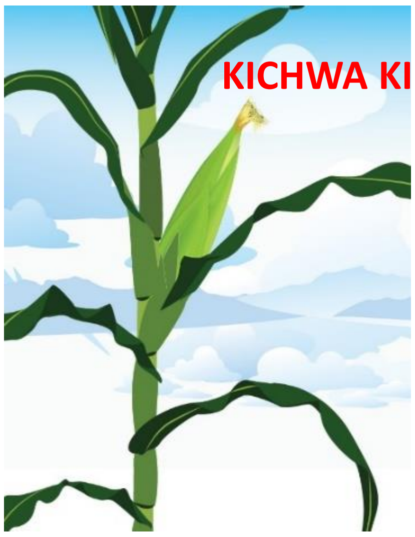

KICHWA KIMEFUNGA

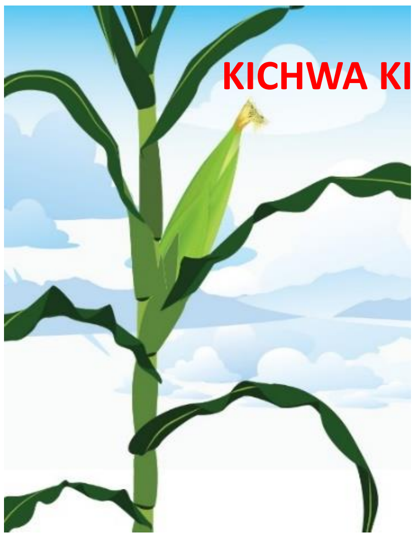

KICHWA KIMEFUNGA

KINAHITAJI MIFUKO MINGI YA MBOLEA YA TOP DRESSING

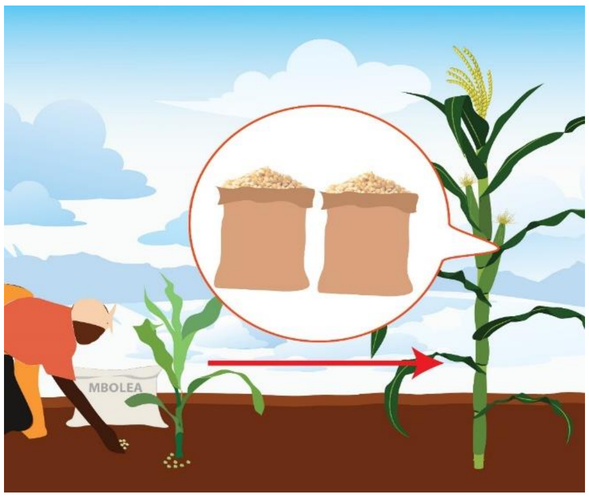

KINATOSHEKA NA MIFUKO CHACHE YA MBOLEA YA TOP DRESSING

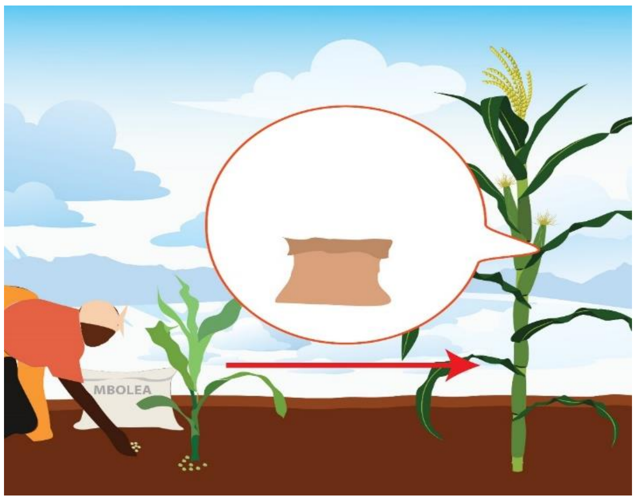

AINA YA MAHINDI –Ya Kwanza

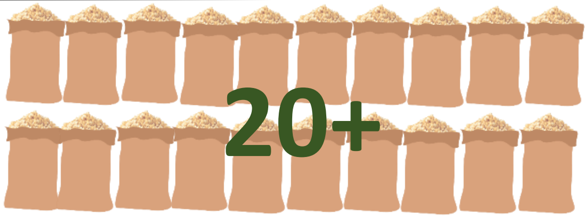

INATOA MAZAO
GUNIA 20 NA ZAIDI
KWA EKARI MOJA

MBEGU
NYEPESI-NYEPESI

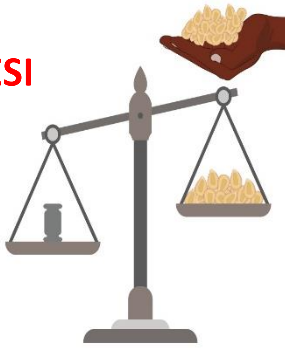

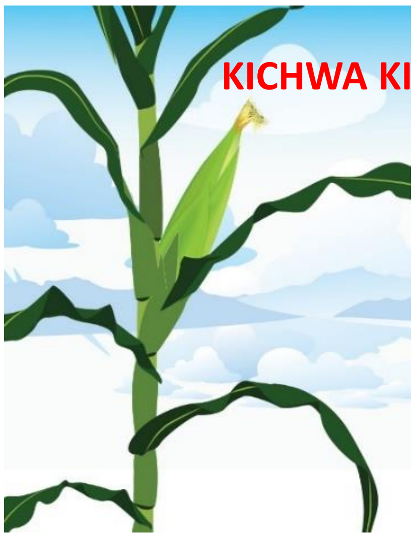

KICHWA KIMEFUNGA

KINATOSHEKA NA MIFUKO CHACHE YA MBOLEA YA TOP DRESSING

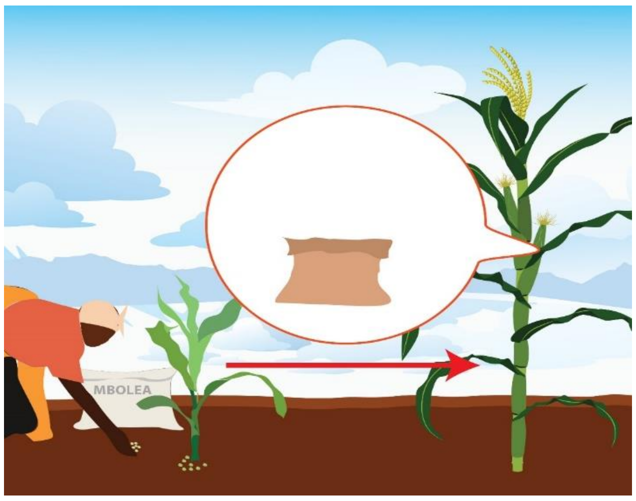

AINA YA MAHINDI –Ya Pili

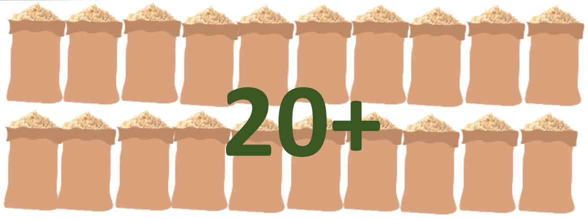

INATOA MAZAO
GUNIA 20 NA ZAIDI
KWA EKARI MOJA

MBEGU
NZITO-NZITO

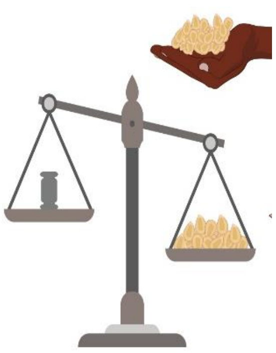

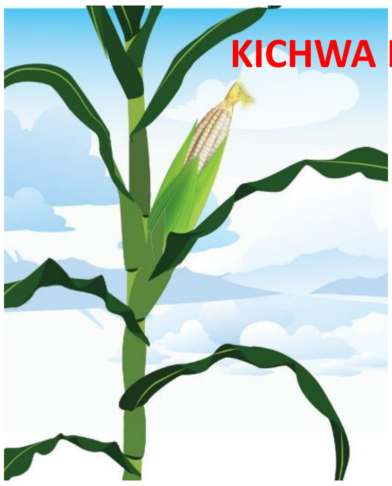

KICHWA KIKO WAZI

KINAHITAJI MIFUKO MINGI YA MBOLEA YA TOP DRESSING

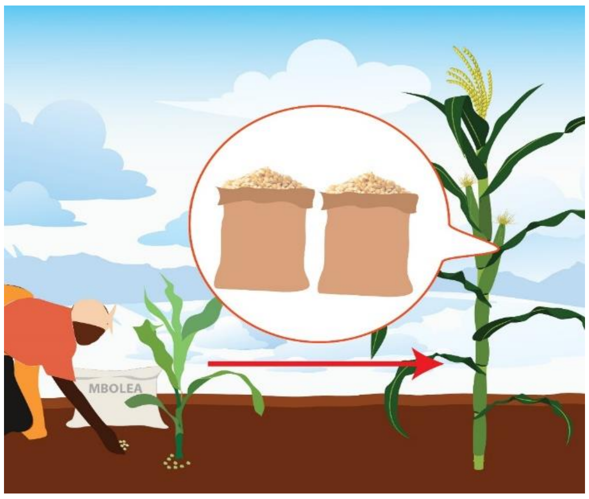

AINA YA MAHINDI –Ya Kwanza	AINA YA MAHINDI –Ya Pili
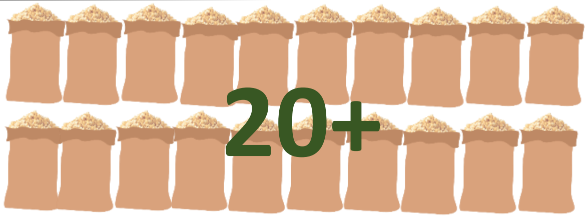 INATOA MAZAO GUNIA 20 NA ZAIDI KWA EKARI MOJA	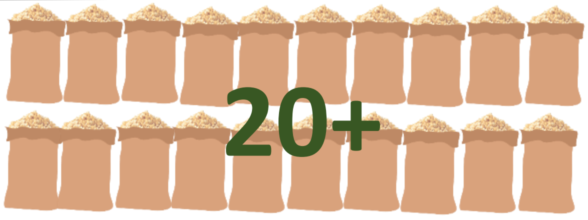 INATOA MAZAO GUNIA 20 NA ZAIDI KWA EKARI MOJA
MBEGU NZITO-NZITO 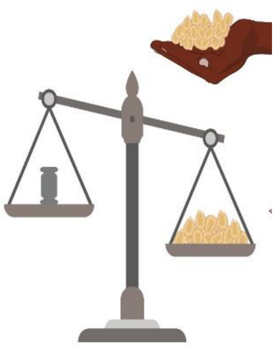	MBEGU NYEPESI-NYEPESI 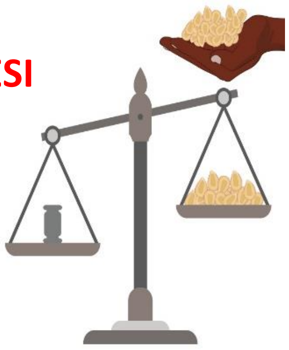
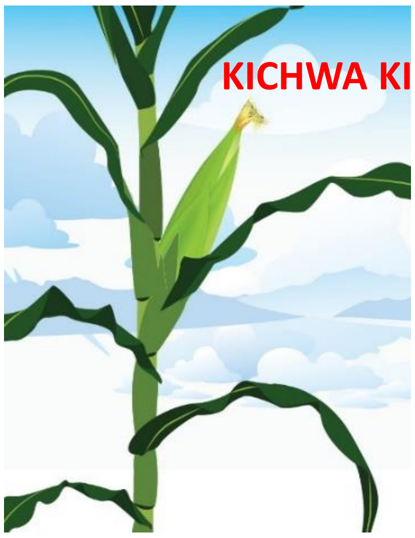 KICHWA KIMEFUNGA	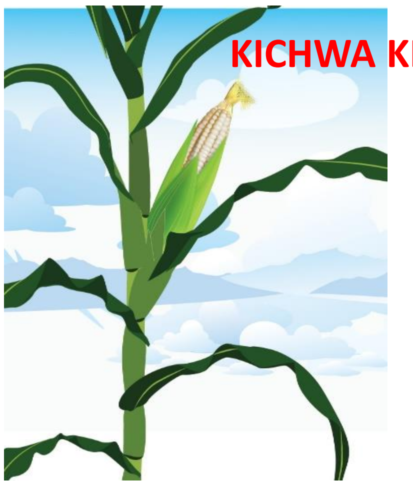 KICHWA KIKO WAZI
KINATOSHEKA NA MIFUKO CHACHE YA MBOLEA YA TOP DRESSING 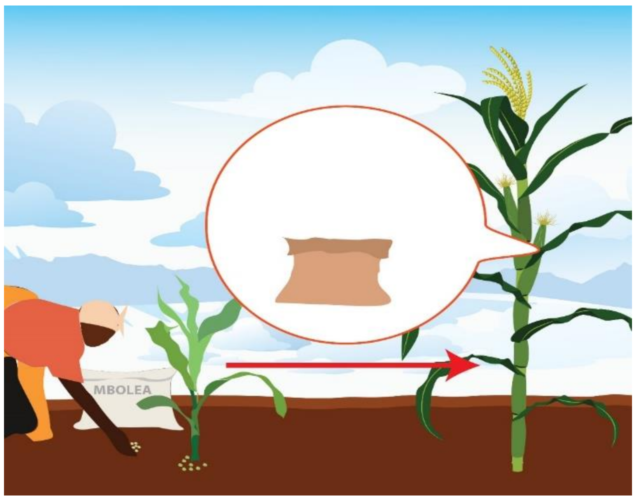	KINAHITAJI MIFUKO MINGI YA MBOLEA YA TOP DRESSING 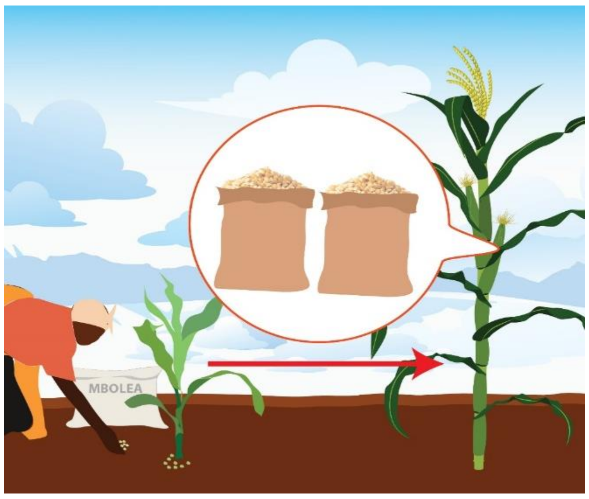

AINA YA MAHINDI –Ya Kwanza

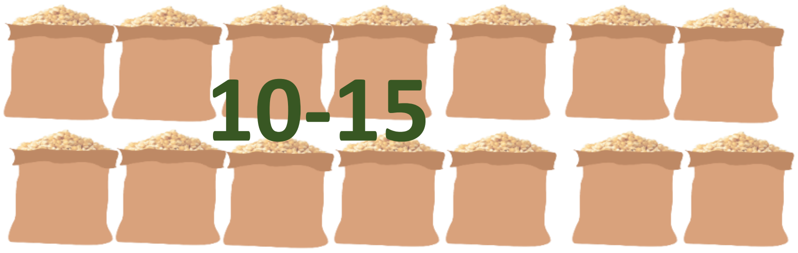

MAZAO GUNIA 10 HADI 15
KWA EKARI MOJA

MBEGU
NYEPESI-NYEPESI

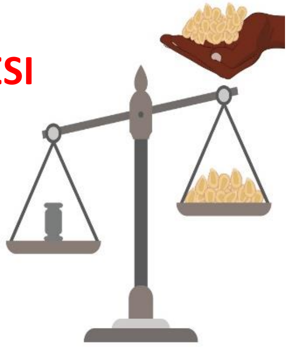

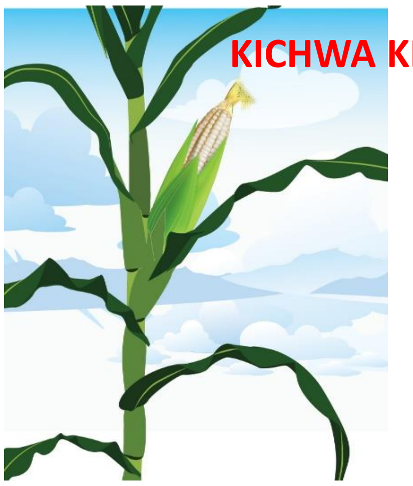

KICHWA KIKO WAZI

KINAHITAJI MIFUKO MINGI YA MBOLEA YA TOP DRESSING

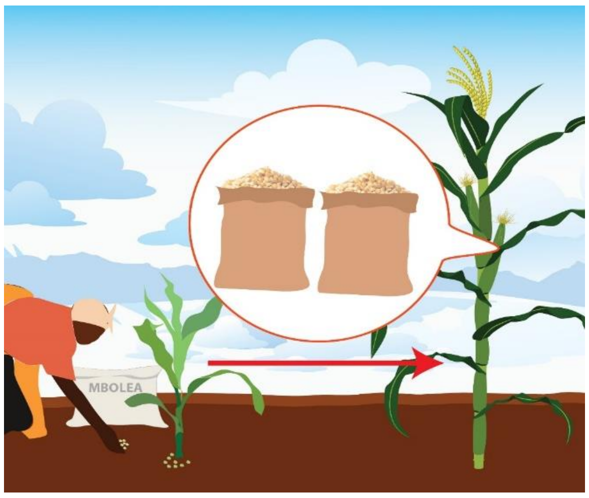

AINA YA MAHINDI –Ya Pili

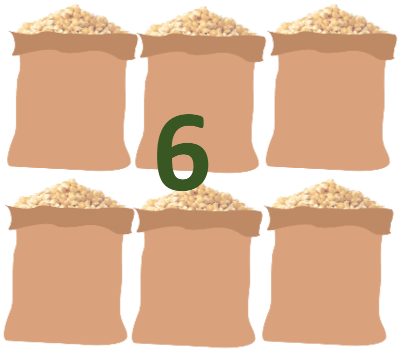

MAZAO GUNIA 6 AU CHINI KWA
EKARI MOJA

MBEGU
NZITO-NZITO

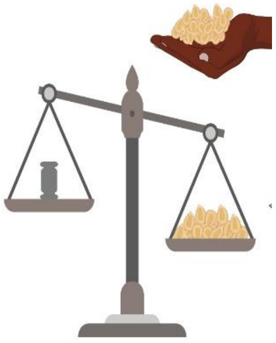

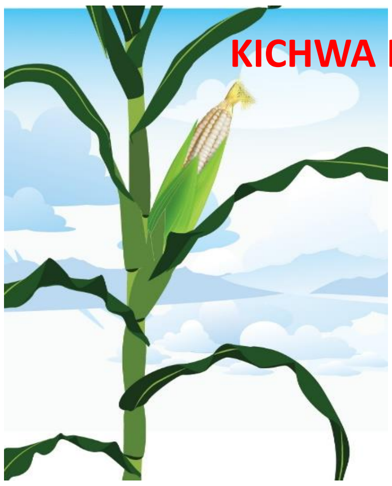

KICHWA KIKO WAZI

KINATOSHEKA NA MIFUKO CHACHE YA MBOLEA YA TOP DRESSING

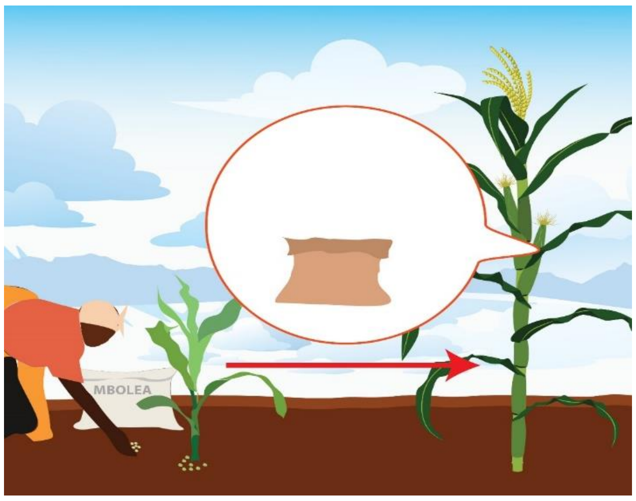

CE-B-Session 11

AINA YA MAHINDI –Ya Kwanza	AINA YA MAHINDI –Ya Pili
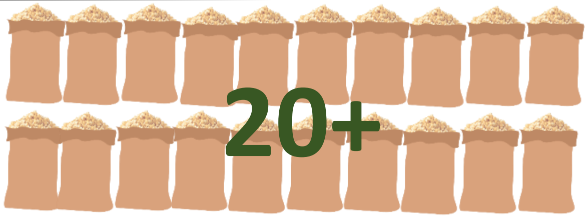 20+ INATOA MAZAO GUNIA 20 NA ZAIDI KWA EKARI MOJA	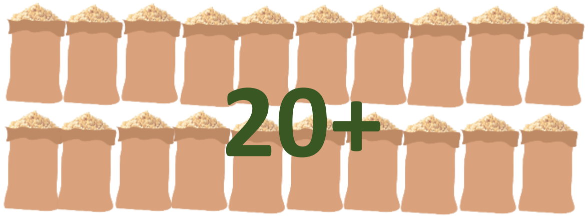 20+ INATOA MAZAO GUNIA 20 NA ZAIDI KWA EKARI MOJA
MBEGU NZITO-NZITO 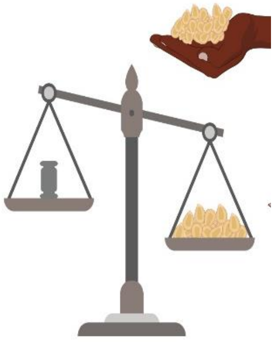	MBEGU NYEPESI-NYEPESI 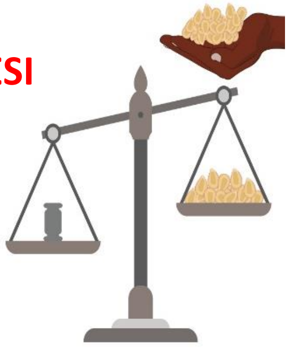
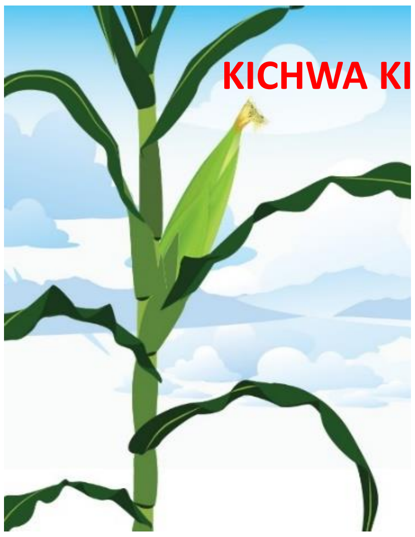 KICHWA KIMEFUNGA	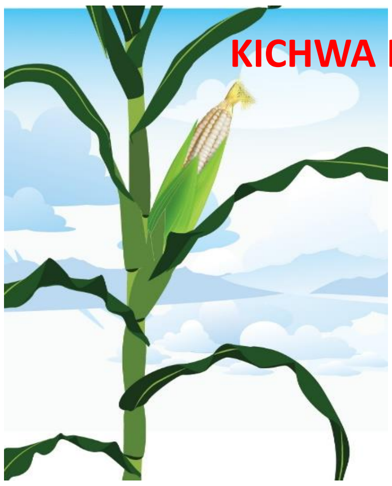 KICHWA KIKO WAZI
KINAHITAJI MIFUKO MINGI YA MBOLEA YA TOP DRESSING 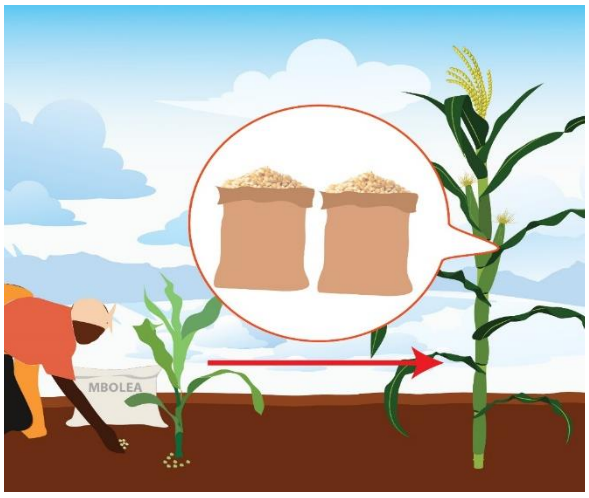 MBOLEA	KINATOSHEKA NA MIFUKO CHACHE YA MBOLEA YA TOP DRESSING 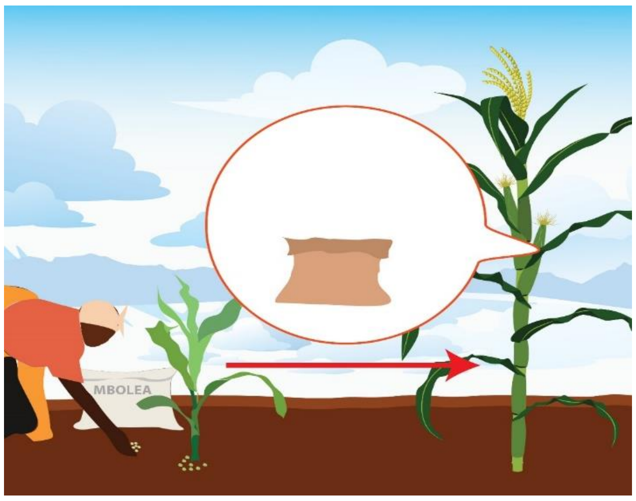 MBOLEA
